# Uncertainty-aware quantitative analysis of high-throughput live cell migration data

**DOI:** 10.1371/journal.pcbi.1014472

**Published:** 2026-07-13

**Authors:** Simo Kitanovski, Shannon Conroy, Justin Sonneck, Lukas Claas, Madeleine Dorsch, Sebastian Urban, Jianxu Chen, Markus Kaiser, Barbara M. Grüner, Daniel Hoffmann

**Affiliations:** 1 Bioinformatics and Computational Biophysics, University of Duisburg-Essen, Essen, Germany; 2 West German Cancer Center, Department of Medical Oncology, Cell Plasticity and Metastasis, University Duisburg-Essen, Essen, Germany; 3 Leibniz-Institut für Analytische Wissenschaften - ISAS - e.V., Dortmund, Germany; 4 Faculty of Computer Science, Ruhr-Universität Bochum, Bochum, Germany; 5 Chemical Biology, University of Duisburg-Essen, Essen, Germany; 6 Center for Medical Biotechnology, University of Duisburg-Essen, Essen, Germany; 7 German Cancer Consortium (DKTK), Partner site University Hospital Essen, Essen, Germany; 8 Center for Computational Sciences and Simulation, University of Duisburg-Essen, Essen, Germany; Lehigh University, UNITED STATES OF AMERICA

## Abstract

Cell migration is a fundamental biological process essential for embryonal development, immune function, and cancer metastasis, with migration velocity representing a key parameter of this behaviour. Today, cell migration velocity can be measured in high-throughput assays that generate complex, hierarchically structured datasets with technical noise, batch effects, and biological variability, introducing significant uncertainty in velocity estimates. Current statistical approaches often fail to rigorously quantify this uncertainty, limiting reproducibility and comparisons across independent experimental datasets. Here, we present *cellmig*, a specialized computational tool that addresses this challenge. It implements established Bayesian hierarchical modeling within an accessible workflow tailored for high-throughput live cell migration assays, to separate biological signals from technical variation while explicitly quantifying uncertainty in migration velocity. *cellmig* provides a robust framework for analyzing cell migration assays, including dose-response studies and large-scale screens with multiple biological and technical replicates. By modeling biological variability (e.g., compound-dependent effects) and technical confounders (e.g., batch variability) within a unified Bayesian framework, *cellmig* estimates condition-specific effects on cell velocity with probabilistic uncertainty intervals, avoiding common pitfalls associated with null-hypothesis testing. Through exhaustive benchmarking against commonly used approaches in the field, we demonstrate that *cellmig* achieves improved sensitivity in detecting subtle migration effects and enhanced robustness against technical variability. Additionally, its generative models enable simulation of migration velocities under various assumptions, aiding experimental planning. We validated *cellmig* through a tiered strategy: (1) benchmarking on two independent experimental datasets and (2) deployment on a large-scale high-throughput screen that discovered new chemical biology. Our results demonstrate that *cellmig* can detect subtle dose-dependent velocity changes, maintain robustness against systematic variability and batch effects, and facilitate reliable integration of multi-experiment datasets. In summary, *cellmig* enhances reproducibility, reliability, and biological insight in high-throughput migration studies, facilitating quantitative inter-dataset comparisons. *cellmig* is implemented as an open-source R package and is freely available on Bioconductor (https://bioconductor.org/packages/cellmig).

## Author summary

Cell migration velocity varies across biological processes–from immune responses to cancer metastasis. Yet accurate measurement remains challenging due to inherent cell-to-cell variability and systematic differences between biological conditions. High-throughput migration assays can track thousands of cells simultaneously under diverse conditions, but they generate complex nested datasets spanning multiple experimental runs with technical and biological replicates. A key challenge is to quantify uncertainty in these multi-experiment datasets, and to reliably detect migration differences. To address this, we developed *cellmig*: an open-source computational tool that implements probabilistic models. Our approach analyzes cell velocity while rigorously accounting for data hierarchy, batch effects, and noise. It provides robust estimates with uncertainty intervals and even simulates data to optimize experimental design. We demonstrate *cellmig*’s capabilities using multi-experiment cancer cell migration data. *cellmig* extracts reliable biological knowledge from cell migration assays and in this way can provide essential information for cancer biology, immunology, and drug development.

## Introduction

Cell migration is essential for many vital biological functions, for instance division or differentiation, and it impacts pathological processes including cancer metastasis [1–4]. Diverse experimental methods have been developed aimed at quantifying different facets of cellular migration, including velocity, path linearity, average turning angle, time persistence or directional bias [5]. These methods depend on a variety of variables, such as the cell type, exposure to chemical or physical stimuli and cell-cell interactions [6].

The most common method for studying live cell migration involves tracking cells with time-lapse microscopy followed by the acquisition of images at fixed time intervals over several hours [6,7]. A high-throughput version of this method facilitates the analysis of cell velocity across dozens of wells in multi-well plates via automated imaging and batch processing, enabling the extraction of quantitative velocities and the detection of changes following perturbations such as drug treatments [8–10]. However, these workflows generate considerable technical noise (e.g., imaging artifacts, imprecise pipetting) and biological variability (e.g., cell-to-cell heterogeneity), and therefore technical and biological replicates are necessary, leading to large, hierarchically structured datasets: cells are nested within technical replicates that are nested within biological replicates [11,12].

Current statistical analyses of such data rely on qualitative approaches (e.g., S1 Fig and 4 from [13]) or inadequate statistical methods, e.g., the nonparametric Wilcoxon or Kruskal-Wallis rank-sum tests [12,14–17]. These approaches usually ignore the hierarchical structure of the data and fail to explicitly quantify uncertainty arising from technical or biological variability, reducing reproducibility.

While Bayesian hierarchical models offer a rigorous alternative, their effective application requires advanced statistical training. Although general-purpose frameworks exist for model specification and fitting, such as *brms* [18], they still require users to specify complex model formulas that account for all relevant sources of variability and the nested structure of their experimental design, perform model comparison to verify the structure is appropriate, diagnose convergence issues, and interpret high-dimensional posterior outputs. Consequently, these methodological challenges remain unresolved for most experimental biologists, who lack formal training in advanced statistics, forcing reliance on simpler but inadequate methods.

To address this gap, we present *cellmig*, an R package that makes rigorous statistical analysis accessible for cell migration studies. *cellmig* automatically handles the complexity of high-throughput migration data—accounting for variation between individual cells, wells, and experimental plates—and returns treatment effect estimates with clear uncertainty measures. The package also includes simulation functionality to help researchers determine how many replicates are needed before running costly experiments. *cellmig* is designed for single-cell velocity data with hierarchical structure (cells nested within wells within plates) and control treatments for batch correction. It is not suitable for assays producing well-level summaries (e.g., wound closure gap area), population-level metrics (e.g., optical flow, collective migration), or experimental designs without replicate structure. This focused scope on statistical inference from tracked velocities enables rigorous uncertainty quantification tailored to high-throughput live cell migration data. By integrating these features into an accessible tool, *cellmig* enables quantitative research and enhances reproducibility. After benchmarking on two independent experiments (about 30,000 cells from 20 biological conditions), we applied *cellmig* to a high-throughput discovery experiment (about 80,000 cells from 80 biological conditions), uncovering new chemical biology while validating statistical robustness.

## Materials and methods

**Fig 1:**
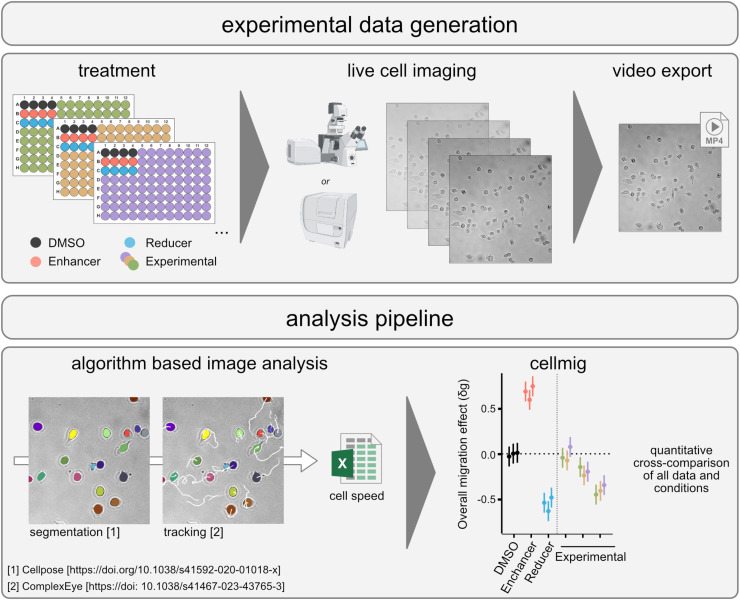
(Top) Live-cell imaging data is collected from multiple 96-well plates, each treated with matching controls (vehicle control, reducer, enhancer of migration) and unique experimental compounds in independent biological experiments. Still-frame images are captured at set intervals and stitched into a single video per well. (Bottom) To quantify single-cell migration velocity, videos undergo segmentation and tracking. The average velocity of each cell is calculated and exported as a spreadsheet for all experiments. *cellmig* enables quantification and cross-comparison of conditions using matching controls across plates. Parts of this figure were created in BioRender. Grüner, B. (2026) https://BioRender.com/rpf419l.

**Fig 2:**
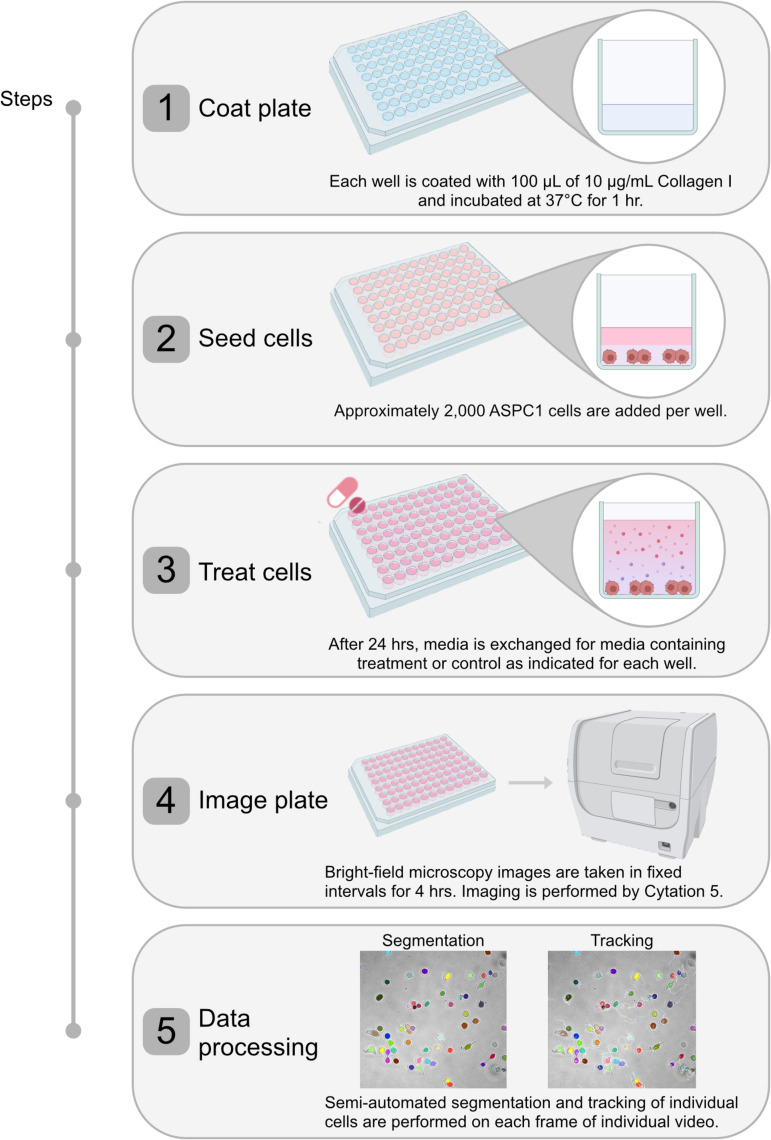
Workflow for live cell imaging and single cell tracking of ASPC1 cells. Multi-well plates were first coated with 100 µL per well of 10 µg/mL collagen I and incubated at 37 °C for 1 hr to support cell attachment. ASPC1 cells were then seeded at a density of approximately 2,000 cells per well and allowed to adhere under standard conditions. After 24 hrs, the culture medium was exchanged for fresh medium containing either the indicated treatment or control. Following treatment for 24 hrs, plates were transferred to a Cytation 5 imaging system for bright-field live cell imaging. Images were taken at fixed time intervals over a period of 4 hrs. The image sequences were processed using a semi-automated analysis pipeline in which individual cells were segmented and tracked across consecutive frames of each video. This workflow enables frame-by-frame quantification of single-cell migration in response to the indicated experimental conditions. Parts of this figure were created in BioRender. Grüner, B. (2026) https://BioRender.com/rpf419l.

We integrate high-throughput live cell imaging with Bayesian statistical modeling to quantify cell migration velocity under uncertainty. To ensure clarity, this section is organized to first describe the hierarchical data structure, followed by the *cellmig* statistical framework, and concludes with experimental protocols. The overall study design is summarized in Fig 1, while the detailed analytical workflow is depicted in Fig 2. We note that the hierarchical Bayesian framework presented here follows established statistical principles [18,19]. Our contribution lies in tailoring this framework to cell migration data structures and providing an accessible implementation for experimental biologists. This scope focuses on statistical inference from quality controlled and processed cell migration data rather than image-processing uncertainty; specifically, *cellmig* does not perform image processing and does not quantify uncertainty arising from segmentation, tracking, or other upstream image analysis and quality control steps. Consequently, users must ensure input data have undergone rigorous quality control (which is standard according to good scientific practice), as valid statistical inference relies on the accuracy of these upstream measurements. This design ensures compatibility with diverse tracking workflows while providing rigorous error estimation for downstream statistical analysis.

### Cell migration data

To validate *cellmig*, we adopted a tiered experimental strategy comprising a benchmarking phase (Dataset 1) and an application phase (Dataset 2). Dataset 1 combines two independent experiments (A and B) designed to test the model’s ability to handle technical variability. By conducting experiments A and B in independent batches with slightly different imaging frame rates, we introduced systematic batch effects, which the model corrects using shared control treatments on each plate. Dataset 2 serves as an independent, large-scale chemical screen to assess scalability and discover new biological insights. Detailed experimental protocols, including cell culture, compound preparation, and imaging parameters, are provided in the subsequent *Experimental methods* sections below. The following subsections detail the hierarchical structure of the processed data tables derived from these experiments.

#### Dataset 1: ASPC1 cells treated across 20 compound-dose combinations in two experiments.

Dataset 1 is represented by a table with 29,503 rows (individual ASPC1 pancreatic cancer cells) and multiple columns representing cell-specific features, including:

- mean cell velocity $y_{i}\in {\mathbb{R}}^{+}$
- well identifier, indexed as w[i]∈{1,2,…,338}
- plate identifier, indexed as p[w]∈{1,2,…,8}
- wells on plates p∈{1,2,3} (generated as part of experiment A) and p∈{4,5,6,7,8} (generated as part of experiment B) were each treated with the same sets of 13 treatments; in total, there were 20 distinct treatments, indexed by t∈{1,2,…,20}, with only three treatments common to both plate groups
- each treatment represents a combination of a chemical compound and its administered concentration, applied across 4 wells per plate (referred to as technical replicates)

We observed between 10 and 275 cells per well (mean = 87 cells). Cell velocities ranged from 0.04 to 4 µm/min (mean velocity = 0.76 µm/min). Velocities on plates p∈{1,2,3} were systematically lower compared to those on p∈{4,5,6,7,8}, attributable to slight differences in imaging configurations used for the two plate groups. This batch effect is corrected by our model, as described in the following sections, which was developed and benchmarked based on Dataset 1 and applied based on Dataset 2.

#### Dataset 2: Large-scale screen of ASPC1 cells treated across 80 compound-dose combinations.

Dataset 2 is represented by a table with 84,122 rows (individual ASPC1 cells) and multiple columns representing cell-specific features, including:

- mean cell velocity $y_{i}\in {\mathbb{R}}^{+}$
- well identifier, indexed as w[i]∈{1,2,…,1109}
- plate identifier, indexed as p[w]∈{1,2,…,18}
- each treatment represents a combination of a chemical compound and its administered concentration, applied across 3–4 wells per plate (referred to as technical replicates)

We observed between 3 and 303 cells per well (mean = 76 cells). Cell velocities ranged from 0.04 to 3.25 µm/min (mean velocity = 0.65 µm/min). Mean velocities per plate varied from 0.55 to 0.85 µm/min.

### Statistical modeling of cell migration velocity

We developed a Bayesian hierarchical regression model (*M*) to estimate treatment effects on cell migration velocity while accounting for experimental structure (technical/biological replicates, plate effects) and pooling information across groups.

This Bayesian approach is advantageous because it allows use of: (i) prior information (e.g., knowledge of effect magnitudes), (ii) explicit modeling of the experiment-specific structure (e.g., nested biological and technical replicates) and (iii) partial pooling of information across groups [19].

#### Model structure.

The observed migration velocity $y_{i}$ for cell *i* was modeled using a gamma likelihood. The gamma distribution was chosen for the likelihood due to its flexibility in modeling positive, right-skewed data:


$$\begin{array}{c} p(y_{i}|M)=Gamma(\kappa_{w[i]},\lambda_{w[i]}) \end{array}$$ (1)

where *w*[*i*] is the index of well of cell *i*, and $\kappa_{w[i]}\in {\mathbb{R}}^{+}$ and $\lambda_{w[i]}\in {\mathbb{R}}^{+}$ are the shape and rate parameters of that well. The mean ($\mu_{w[i]}\in {\mathbb{R}}^{+}$) of the gamma distribution is the ratio of $\kappa_{w[i]}$ to $\lambda_{w[i]}$, which allows reparameterization of the likelihood in terms of $\kappa_{w[i]}$ and $\mu_{w[i]}$ as:


$$\begin{array}{c} p(y_{i}|M)=Gamma(\kappa_{w[i]},\frac{\kappa_{w[i]}}{\mu_{w[i]}}) \end{array}$$ (2)

This parameterization expresses the gamma likelihood in terms of mean (μ) and shape (κ) rather than the canonical shape-rate form, facilitating direct interpretation of treatment effects on the natural velocity scale. By modeling all cells within a well through shared parameters $\mu_{w}$ and $\kappa_{w}$, information is pooled across cells to produce stable well-level estimates even when individual wells contain varying numbers of tracked cells.

**Well-level model: Accounting for technical replicates and batch effects:** Each well *w* belongs to a plate p∈{1,2,…,P} (biological replicate) and is treated by a treatment t∈{1,2,…,T}. Multiple wells per plate may receive the same treatment (technical replicates, indexed as *pt*[*w*]). The log-transformed means of wells receiving the same treatment are modeled hierarchically, by treating the corresponding coefficients ($\log(\mu_{pt[w]})$) as random samples drawn from normal distributions:


$$\begin{array}{c} \log(\mu_{pt[w]})\sim Normal(\gamma_{pt},\sigma_{\text{tech}}), \end{array}$$ (3)

where $\gamma_{pt}$ is mean effect of treatment *t* on plate *p*; and $\sigma_{\text{tech}}$ captures the variabili*t*y between technical replicates. However, we know that cell migration assays are affected by plate-specific batch effects that can systematically bias the migration velocity across all wells on the plates. To correct for plate-specific batch effects, we include an offset term $\alpha_{p}$, defined as the mean migration velocity (on log-scale) of a control treatment (e.g., dimethyl sulfoxide (DMSO)) on plate *p*, effectively normalizing batch effects across plates:


$$\begin{array}{c} \left\{ \begin{array}{cc} \log(\mu_{pt[w]})\sim Normal(\alpha_{p},\sigma_{\text{tech}}), & \text{if}tisthecontroltreatment\}\\ \log(\mu_{pt[w]})\sim Normal(\alpha_{p}+\gamma_{pt},\sigma_{\text{tech}}), & \text{otherwise} \end{array}\right. \end{array}$$ (4)

where the treatment effect $\gamma_{pt}$ captures deviations from this baseline for treatment *t* on plate *p*; and $\sigma_{\text{tech}}$ captures the variability between technical replicates. This structure pools information across technical replicates, producing more stable estimates than analyzing each well independently while still allowing for well-to-well variation.

**Plate-level model: Accounting for biological replicates:** Wells treated with the same treatment (*t*) will have similar cell migration velocities (similar $\gamma_{pt}$ coefficients) after accounting for batch effects. Hence, the model treats $\gamma_{pt}$ as random samples drawn from normal distributions:


$$\begin{array}{c} \gamma_{pt}\sim Normal(\delta_{t},\sigma_{\text{bio}}) \end{array}$$ (5)

where $\delta_{t}$ is the overall (across plates) effects of treatment *t* on cell migration; and $\sigma_{\text{bio}}$ is the standard deviation between biological replicates. The hierarchical structure enables sharing of information across plates while preserving treatment-specific estimates.

**Treatment-level model: partial pooling across treatments:** High-throughput cell migration screens enable researchers to characterize treatment effects of hundreds or even thousands of compounds simultaneously. In such high-dimensional scenarios, the peril of false positive effects increases due to multiple testing. *cellmig* implements a hierarchical layer that treats the overall treatment effects ($\delta_{t}$) as draws from an overarching population of effects:


$$\begin{array}{c} \delta_{t}\sim Normal(0,\sigma_{\delta }) \end{array}$$ (6)

where $\sigma_{\delta }$ is the standard deviation between $\delta_{t}$ estimates. This hierarchical structure enables partial pooling (shrinkage) of information across treatments [19]. Parameters with high uncertainty (e.g., due to low cell counts or high variability) are pulled more strongly toward the population mean (zero effect), while well-estimated parameters remain data-driven.

This shrinkage mechanism inherently regularizes the model against false positives in large-scale screening. By stabilizing estimates for weak signals, it improves the reliability of ranking treatments by their effects ($\delta_{t}$). Thereby, the model automatically controls the amount of pooling that is applied to the treatment-specific parameters, ensuring that prioritization decisions are driven by robust evidence rather than noise.

**Gamma distribution shape parameter modeling:** We observed a linear relationship between the means and variances of the gamma-distributed velocities across wells on a log–log scale (S2A Fig), as expected from the analytic mean ($\mu_{w}=\kappa_{w}/\lambda_{w}$) and variance ($\tau_{w}=\kappa_{w}/\lambda_{w}^{2}$) of the Gamma distribution. Across wells, the variances were on average approximately five-fold smaller than the means (S2A Fig), indicating stable measurements and controlled experimental conditions with a small coefficient of variation (CV):


$$\begin{array}{c} {CV}_{w}=\frac{\sqrt{\tau_{w}}}{\mu_{w}}\ll 1. \end{array}$$ (7)

Notably, the Gamma shape parameter satisfies $\kappa_{w}=\mu_{w}^{2}/\tau_{w}=1/{CV}_{w}^{2}$, i.e., it is the inverse squared coefficient of variation. The empirical distribution of the inverse squared CV in Dataset 1 was approximately normal on a logarithmic scale (S2B Fig). Hence, we assumed that values $\log(\kappa_{w})$ are also approximately normally distributed, and treated them as random samples drawn from a normal distribution with mean $\mu_{\kappa }$ and standard deviation $\sigma_{\kappa }$:


$$\begin{array}{c} \log(\kappa_{w})\sim Normal(\mu_{\kappa },\sigma_{\kappa }). \end{array}$$ (8)

#### Quantifying treatment effects.

For treatments *i* and *j*, the contrast $\rho_{ij}=\delta_{i}-\delta_{j}$ represents the log-fold change in migration velocity. If ρ< 0 and the 95% Highest Density Intervals (HDIs) of ρ lie mostly or completely below 0 (0 = null effect), we have strong evidence of reduced cell velocity in *i* relative to *j*. On the other hand, if ρ and the 95% HDI of ρ lie mostly or completely above 0, we have strong evidence of increased cell velocity in *i* compared to *j*. Distributions with the 95% HDIs more or less centered around 0 indicate that there is no evidence for a clear change in the cell velocity. Note that unclear evidence is not equivalent to no change, because for a treatment group with ρ≈ 0 we may also have a wide 95% HDI, including possibilities for positive or negative change. *cellmig* outputs mean and 95% HDI of ρ, including the probability of differential effect on migration (π):


$$\begin{array}{c} \pi =2\cdot \max\left(\int_{-\infty }^{0}p(\rho )d\rho ,\int_{0}^{\infty }p(\rho )d\rho \right)-1 \end{array}$$ (9)

Here, π≈1 indicates strong evidence for a difference in cell migration velocity between the treatments, while π≈0 suggests negligible evidence.

For easier interpretation, *cellmig* exponentiates the log-fold-changes described by $\delta_{t}$, $\gamma_{pt}$, and $\rho_{ij}$, yielding fold-changes $\delta_{t}^{\prime }$, $\gamma_{pt}^{\prime }$, and $\rho_{ij}^{\prime }$, respectively. Throughout the Results, we present effects on the natural scale (fold-change), where the null effect equals 1 (no change).

#### Model priors.

Our Bayesian framework requires prior distributions for location parameters ($\alpha_{p}$, $\mu_{\kappa }$) and scale parameters ($\sigma_{\delta }$, $\sigma_{\text{tech}}$, $\sigma_{\text{bio}}$, $\sigma_{\kappa }$). We adopted a dual strategy: literature-informed priors for location parameters to ensure biological plausibility across cell types, and regularizing priors for scale parameters to handle variance components where literature is scarce. We validated these choices through prior predictive checks and prior sensitivity analysis, confirming that the priors generate realistic data without unduly constraining posterior inferences (see *Validation via prior predictive checks* and *Validation via prior sensitivity analysis* below). The following subsections detail the justification for each prior choice.

**Location parameters:** $\alpha_{p}$ and $\mu_{\kappa }$: Location parameters define the baseline velocity and Gamma distribution shape. To ensure *cellmig* accommodates diverse cell types—from slow epithelial cells to fast immune cells—we elicited priors using three data sources: the World Cell Race study [20], high-throughput neutrophil data [9], and our experimental datasets.

The parameter $\alpha_{p}$ represents the baseline migration velocity (log-scale) of control treatments (e.g., DMSO) on plate *p*. The World Cell Race study [20] reported mean velocities for 54 cell types ranging from 0.1 to 0.9 µm/min (log-scale means: -2.3 to -0.1), with maximum individual velocities up to 5.2 µm/min (log-scale: 1.65). Combining these sources with our empirical data from Dataset 1 (range of mean well velocities 0.3–1.63 µm/min; log-scale means: -1.2 – + 0.3), we assigned:


$$\begin{array}{cc} \alpha_{p} & \sim Normal(-0.5,1.0) \end{array}$$ (10)

This prior places 99% of density between -3 and 2 on the log-scale (0.05 to 7.4 µm/min), accommodating both slow and fast migration.

We validated this prior choice against control-treated neutrophil data [9]. Neutrophils have velocities an order of magnitude higher than tumor cells. While neutrophil velocities fall in the right tail of our prior, they remain within high-density regions (S2C Fig), confirming the prior accommodates faster cell types without being overly diffuse.

The parameter $\mu_{\kappa }$ defines the population mean of $\log(\kappa_{w})$, where $\kappa_{w}=1/{\text{CV}}_{w}^{2}$ controls the skewness of the velocity distribution. To assign a prior on $\mu_{\kappa }$ we examined the means and standard deviations of 54 cell types reported in [20], observing that standard deviations were typically an order of magnitude smaller than means. From Fig 1D of that study, we extracted approximate means and standard deviations for ten cell types and computed $\log(1/{\text{CV}}^{2})$, yielding values around 3.3 on the log-scale. These values exceeded our empirical observations from Dataset 1 (mean = 1.55, standard deviation (SD) = 0.4) and Dataset 2 (mean = 1.52, SD = 0.45), likely reflecting lower measurement variability in the low-throughput World Cell Race study compared to our standardized high-throughput assay. Second, we computed well-specific $\log(1/{\text{CV}}^{2})$ values for high-throughput control-treated neutrophils [9], which yielded values centered around 0 on the log-scale with SD = 0.4, i.e., somewhat lower than our empirical observations from Datasets 1 and 2. To ensure the model accommodates the variability inherent to both low-throughput assays and large-scale high-throughput screens, we centered the prior at 1.5 with an inflated standard deviation (SD = 1.0):


$$\begin{array}{cc} \mu_{\kappa } & \sim Normal(1.5,1.0). \end{array}$$ (11)

This choice encompasses estimates from the World Cell Race study, neutrophil data, and our empirical datasets (S2B Fig):

**Scale parameters:** $\sigma_{\delta }$, $\sigma_{\text{tech}}$, $\sigma_{\text{bio}}$, $\sigma_{\kappa }$: For variance components, we employed regularizing Half-Normal priors [19]. Comprehensive literature on variance components across high-throughput platforms is lacking; thus, regularizing priors constrain parameters to plausible ranges while allowing data to dominate inference. This approach also facilitates efficient sampling in Stan.

The hyperparameter $\sigma_{\delta }$ controls shrinkage of treatment effects $\delta_{t}$ toward zero (Equation 6): small values pull treatment effects toward zero (appropriate when most compounds have negligible effects), while larger values allow more treatment-specific variation. To justify our prior choice for $\sigma_{\delta }$, we considered the expected magnitude of treatment effects in typical screens. If we assume $\delta_{t}=1$ and ignore the technical and biological variability in Equations 4 and 5, then from Equation 4 it follows that $\mu_{pt[w]}=\exp(\alpha_{p})\cdot \exp(1)$, i.e., the mean cell velocity increases by a factor of exp(1)=2.71 over the baseline cell migration velocity of the plate. However, if $\delta_{t}=2$, then the mean cell velocity would increase by a staggering factor of exp(2)=7.38 over the baseline migration. Using the control treatments in our experiments, we observed $\delta_{t}$=-0.53 (95% HDI=[-0.46, -0.60]) for the compound CK666 at 40 µM, and $\delta_{t}$=0.55 (95% HDI=[0.48, 0.62]) for fluvastatin at 10 µM, which are associated with severe reduction and increase in the cell migration velocity relative to DMSO (control treatment), respectively. Hence, for most treatments we expected $|\delta_{t}|<1$, which implied that we need a prior distribution that assigns high density to values around 0, and low density to values larger than 1 and smaller than −1. In fact, even if CK666 or fluvastatin were chosen as the the control treatment, $\left|\delta_{t}\right|$ barely crosses the magnitude 1. Hence, for most treatments in a typical screen we expected $|\delta_{t}|<1$, as the majority of compounds are expected to have negligible effects on migration. This sparsity assumption constrains the plausible range for $\sigma_{\delta }$. Since $\delta_{t}$ values are drawn from a Normal distribution with standard deviation $\sigma_{\delta }$, most $\delta_{t}$ values (approximately 95%) will fall within roughly two standard deviations of zero ($\pm 2\cdot \sigma_{\delta }$). To ensure most treatment effects remain within our observed range of $|\delta_{t}|<1$, we need $\sigma_{\delta }$ around 0.5 (since 2×0.5=1). To allow for occasional strong effects up to $|\delta_{t}|\approx 2$ for potent compounds, we need $\sigma_{\delta }$ up to 1.0 (since 2×1.0=2). Therefore, $\sigma_{\delta }$ should be approximately 0.5–1.0 to balance the expectation that most compounds have negligible effects while remaining flexible enough to detect genuinely strong effects. We therefore assigned a regularizing half-normal prior:


$$\begin{array}{cc} \sigma_{\delta } & \sim {Normal}^{\text{+}}(0,1) \end{array}$$ (12)

This prior places 95% of density below approximately 2, with median around 0.67, concentrating density in the 0.5–1.0 range that matches our empirical observations while allowing larger values for screens with more heterogeneous treatment effects. This ensures shrinkage is strong enough to control false positives without being so restrictive that it masks genuine large effects (e.g., from newly synthesized compounds with stronger effects).

We applied similar regularizing priors to the remaining scale parameters, capturing technical, biological, and Gamma shape variability. These capture technical (well-to-well), biological (plate-to-plate), and Gamma shape variability, respectively. The magnitude of our observed treatment effects constrains these variance components: since we reliably detected effects as small as $|\delta_{t}|\approx 0.5$ (e.g., CK666, fluvastatin) with narrow uncertainty intervals, $\sigma_{\text{tech}}$ and $\sigma_{\text{bio}}$ must be substantially smaller than typical effect sizes. Empirical estimates from our datasets ranged from 0.1 to 0.45 on the log-scale, consistent with this constraint and falling well within the prior’s high-density region (95% below 2).


$$\begin{array}{cc} \sigma_{\text{tech}} & \sim {Normal}^{\text{+}}(0,1)\\ \sigma_{\text{bio}} & \sim {Normal}^{\text{+}}(0,1)\\ \sigma_{\kappa } & \sim {Normal}^{\text{+}}(0,1) \end{array}$$ (13)

**Validation via prior predictive checks:** To confirm these priors generate biologically plausible data, we performed prior predictive checks (S3 Fig). We compared our default configuration (all scale parameter SD = 1) against wider priors (SD = 3). While posterior estimates remained stable under both configurations, the wider priors generated implausible velocities: over 5% of simulated velocities exceeded 100 µm/min, compared to only 1% under the default priors. Consequently, we retained the default priors (SD = 1) to ensure realistic data generation.

**Validation via prior sensitivity analysis:** To assess whether our prior choices influence posterior inferences, we compared the default *cellmig* model (all scale parameter SD = 1: $\sigma_{\text{bio}}$=1, $\sigma_{\text{tech}}$=1, $\sigma_{\delta }$=1) against a model with wider priors (all scale parameter SD = 3: $\sigma_{\text{bio}}$=3, $\sigma_{\text{tech}}$=3, $\sigma_{\delta }$=3). Both models were fit to Dataset 1 using identical MCMC settings (4 chains, 2,000 iterations, 1,000 warm-up). Posterior estimates for all parameters were nearly identical between the two configurations (S4A Fig): overall treatment effects ($\delta_{t}$), plate-specific effects ($\delta_{tp}$), plate intercepts ($\alpha_{p}$), and variance components ($\sigma_{\text{bio}}$, $\sigma_{\text{tech}}$, $\sigma_{\delta }$) showed differences in posterior means of less than 0.01 on the log-scale. All parameters achieved convergence (Rhat ≈ 1.00) in both models. This demonstrates that the priors are appropriately regularizing—they constrain parameters to plausible ranges without influencing posterior estimates, allowing the data to dominate inference. Consequently, we retained the default priors (SD = 1) for all analyses, as they provide adequate regularization while generating biologically plausible data (see prior predictive checks above).

#### Experiment planning by simulation.

Our Bayesian model (*M*) is generative and can simulate cell velocities using the same error model (Equation 1). In this framework, the values of $\kappa_{w[i]}$ and $\lambda_{w[i]}$ are drawn from their priors, which are themselves informed by parameters in subsequent layers of the model. This simulation process is critical for optimizing experimental design, particularly in determining the optimal number of (i) cells, (ii) technical replicates, and (iii) biological replicates. These factors directly affect the uncertainty in estimating treatment effects ($\delta_{t}$) and their differences ($\rho_{ij}$). The model can operate in two modes: *fully* generative, where all free parameters are sampled from their priors, or *partially* generative, where certain parameters are fixed by the user (e.g., to align with estimates from prior experiments).

Both modes support experimental design but serve distinct purposes. The *fully generative* mode (function gen_full()) draws all parameters from priors, serving as a prior predictive check to validate model generalizability across diverse biological contexts (S3 Fig). The *partially generative* mode (function gen_partial()) allows users to fix specific parameters (e.g., effect sizes, variance components) based on prior data while sampling others from priors. This reflects standard practice in power analysis, where researchers specify anticipated effect sizes to estimate sample size requirements. This flexibility allows users to tailor simulations to their available knowledge: incorporating specific estimates from pilot studies when planning similar experiments, or using broader assumptions when designing novel studies where less prior information is available. We note that the power analysis results presented in the Results section reflect the specific noise structure of our imaging assay. For experimental setups with different variability profiles (e.g., different microscopy systems or cell lines), users should estimate variance components from a small pilot study and use the gen_partial() function to generate power curves tailored to their specific context.

#### Visualization and prioritization of treatment effects.

To facilitate decision-making across varying screen sizes, the *cellmig* function get_pairs() automatically generates appropriate visualizations based on the number of treatments. For modestly sized experiments (treatments < 100), the function produces heatmaps comparing $\rho^{\prime }$ and π across all pairwise conditions, enabling comprehensive inspection of treatment relationships (Fig 5). For large-scale screens involving hundreds or thousands of conditions, where heatmaps become impractical, get_pairs() generates Bayesian volcano plots combining effect size ($\rho^{\prime }$) and support (π) (S5 Fig). Similar to the use of posterior means and b-values in metabolomic screening [21], *cellmig* utilizes $\rho^{\prime }$ to represent biological magnitude and π to extract maximum information from the posterior distribution regarding evidence strength. By balancing effect size against variability, this framework aligns with established high-throughput screening practices for prioritizing promising candidates for validation [22,23], particularly in large-scale settings where traditional visualization becomes impractical.

### Experimental data generation and image processing

#### Cell line and cultures.

ASPC1 cells (American Type Culture Collection (ATCC) Catalog number (Cat#) CRL-1682, Research Resource Identifier (RRID):CVCL_0152, sex: female) were cultured under humidified atmosphere at 37 °C and 5% CO_2_ in Dulbecco’s Modified Eagle’s Medium (DMEM) high glucose, pyruvate (Gibco, Cat # 41966052) supplemented with 10% Fetal bovine serum (FBS) (VWR/Biowest, Cat #S1600-500), 1% non-essential amino acids (ThermoFisher, Cat #11140035), 50 U/ml penicillin and 50 μ g/ml streptomycin (ThermoFisher, Cat #10378016). Identity was authenticated by Short Tandem Repeats (STR) analysis. Testing to exclude mycoplasma contamination was performed regularly.

#### Chemicals.

The following commercially available compounds were used at concentrations as indicated: fluvastatin (abcam, Cat# ab120651, Chemical Abstracts Service Number (CAS No) 93957-55-2), pravastatin (Sigma-Aldrich, Cat# 524403, CAS No 81131-70-6), pitavastatin (Sigma Aldrich, Cat# SML2473, CAS No 147526-32-7), benproperine (Hycultech, Cat# HY114657A, CAS No 19428-14-9), haloperidol (Sigma Aldrich, Cat# H1512, CAS No 52-86-8), CK666 (Sigma Aldrich, Cat# SML0006, CAS No 442633-00-3), CK869 (Sigma Aldrich, Cat# C9124, CAS No 388592-44-7). All compounds were dissolved in DMSO (AppliChem, Cat# A3672, CAS No 67-68-5). The final concentration of DMSO for all compounds and vehicle control was at maximum 0.1% and serially diluted down in lower concentrations, except for pravastatin, where it was 1%, indicated by “DMSO high” (included as additional experimental vehicle control).

For the application screen, to assess structure activity relationships 15 derivatives of the “parent” compound pimozide were synthesized (S6 Fig and S1 Table) and used at the indicated concentrations. All compounds were dissolved in DMSO (AppliChem, Cat# A3672, CAS No 67-68-5). The final concentration of DMSO for all compounds and vehicle control was at maximum 0.1% and serially diluted down in lower concentrations.

#### Assessment of *in vitro* viability.

ASPC1 cells were seeded in triplicate per condition on 96-well plates ($8\times 10^{3}$ to $20\times 10^{3}$ cells/well, depending on the time point). Compounds were added to ASPC1 cells at indicated concentrations. For the setup experiments A and B cell viability was assessed using the PrestoBlue cell viability assay (Invitrogen, Cat #A13262) per manufacturer’s instructions after 0, 24, 48 and 72 hours (S7 Fig). For the application data set, viability was assessed at 0 and 48 hours, at the start and endpoint of the live-cell imaging experiment (S8 and S9 Figs). Absorbance at 570 and 600 nm was measured with a Tecan Spark 10M plate reader (Tecan Trading AG, RRID:SCR_021897).

#### Live-cell imaging.

96-well plates were coated with Collagen I (Sigma Aldrich, Cat #C8919, CAS No 9007-34-5) for 1 hour at 37 °C and 5% CO_2_ to promote cell attachment and migration behavior. Following incubation, the wells were washed once with Phosphate Buffered Saline (PBS). Cells were seeded at 2,000 cells per well in DMEM and allowed to adhere overnight under standard culture condition (37 °C and 5% CO_2_). On the following day, cells were treated with either DMSO vehicle control or the indicated compounds and incubated for 48 hours prior to imaging. For each plate, four technical replicates per condition were imaged.

Time-lapse brightfield imaging was performed using a cell imaging multimode reader (Cytation 5, Agilent Technologies, RRID:SCR_019732). Each plate was imaged with the 20× objective for 4 hours at 37 °C and 5% CO_2_ with a fixed number of kinetic reads/looping times as indicated. Individual images were aligned and stitched into .mp4 files using Gen5 software (RRID:SCR_017317). Quality-control procedures were performed prior to downstream analysis. Videos were excluded if the automated alignment by Gen5 software failed, if substantial stage drift or focus instability was observed, or if non-cellular debris was incorrectly identified as migrating cells. Only wells passing all quality-control criteria were included in subsequent migration analyses.

#### Computational segmentation and tracking.

The tracking workflow was established as previously described [9]. The generated movies, exported as .mp4 files, underwent automated segmentation using the cellular segmentation tool Cellpose [24]. To adapt to the domain-specific characteristics of the ASPC1 cell line, a pretrained Cellpose model was fine-tuned using representative samples. Subsequent single-cell tracking was carried out using a classical tracking algorithm based on the Earth Mover’s Distance [25]. The tracking data contained unique tracking labels for each cell within the segmented images of the time-lapse sequence. Only cells in at least 10 consecutive frames were analyzed.

For each well, approximately 50–200 cells were measured in this way. Distances in micrometers were obtained from pixel displacements using the pixel size (0.348 µm/pixel) and combined with the acquisition interval of one frame every 4 minutes to calculate migration velocity in µm/min.

To ensure the reliability of velocity measurements, we quantified detection and linking errors using an Acyclic Oriented Graphs Matching (AOGM)-inspired framework [26]. We manually curated a representative subset of nine movies (20 frames each) and compared automated outputs against corrected annotations. This analysis identified 153 false-positive detections (fp), 174 false-negative detections (fn), 1 object-splitting operation (sc), 88 edge-deletion operations (de), and 220 edge-addition operations (ae) (S2 Table). Here, fp and fn represent spurious and missed cell detections, respectively; de and ae correspond to redundant/incorrect and missing temporal links, respectively; and sc indicates instances where detected objects required separation. The mean relative error across all error types was below 3.69% (S2 Table). Representative visual examples of segmentation masks and track overlays from the curated movies are provided in S10 Fig.

We further performed a sensitivity analysis to evaluate the impact of tracking artifacts on downstream velocity estimates. Comparing automated tracks (354 tracks, mean velocity = 0.441 µm/min) against manually corrected tracks (360 tracks; mean velocity = 0.438 µm/min); difference due to recovery of false-negative detections revealed a negligible difference in mean velocity (difference = 0.003, 95% confidence interval [-0.028, 0.034]; Welch Two Sample t-test, *p* = 0.85). This indicates that the observed level of tracking artifacts does not substantially affect migration readouts.

### Software availability

*cellmig* is implemented as an open-source R package and is freely available on Bioconductor (https://bioconductor.org/packages/cellmig). The stable release version requires R (version ≥4.5). Key dependencies include *rstan* (≥2.26) for model fitting, and *ggplot2* and *patchwork* for visualization and result presentation. All analyses in this manuscript were performed using *cellmig* version 1.3.4 on R 4.5.1, *rstan* 2.32.7, *ggplot2* 3.5.0, and *patchwork* 1.2.0.

The development version of *cellmig*, including the latest features, is available on GitHub at https://github.com/snaketron/cellmig. Users are able to report bugs, request features, or ask questions through the Bioconductor Support Site or the GitHub issue tracker. This enables direct communication between users and developers to improve the tool.

A comprehensive vignette reproducing the analysis of Dataset 1 and Dataset 2 is available at https://github.com/snaketron/cellmig_paper_data, with additional vignettes for analysis and simulation on Bioconductor. All resources include executable code, expected outputs, and workflow guidance to ensure reproducibility.

### Software used to illustrate figures

Parts of Figs 1 and 2 were created in BioRender. Grüner, B. (2026) https://BioRender.com/rpf419l. GraphPad Prism was used to generate graphs in S1, S7, S8, and S9 Figs. Affinity Designer was used for final editing and layout design of Figs 1 and 2, as well as S1, S7, S8, and S9 Figs. The remaining figures were generated with *ggplot2* and assembled with *patchwork*.

## Results

### Experimental design and computational workflow

To evaluate the performance of *cellmig* in quantifying treatment effects on cell migration velocity, additionally taking into account multiple experimental variables such as technical/biological replicates, and plate variability, we conducted two independent high-throughput experiments (A and B from Dataset 1) using pancreatic cancer cells ASPC1 (Fig 1). This cell line was chosen for its robust migratory activity in live-cell assays [13]. Experimental setups A and B comprised three and five biological replicates, respectively. Each well of the 96-well plate was treated with one of eight compounds at specified concentrations. Each condition was tested in four technical replicates per plate and in at least three independent experiments. While experiments A and B tested largely distinct compound sets, both included the vehicle-only control (DMSO) on all plates at the highest DMSO concentration of the compound-treated conditions. Additional controls included a migration enhancer (fluvastatin 10 µM) and reducer (ARP2/3 complex inhibitor CK666 40 µM). Both enhancer and reducer were included to implement upper and lower baselines to be used for cross-plate and cross-experiment comparison.

In experiment A, experimental compounds included three statins: pitavastatin, fluvastatin, and pravastatin, each tested at 2.5, 5, and 10 µM, to allow for direct dose-response comparison. In this experiment, pitavastatin has a low solubility in DMSO. To therefore account for the higher concentrations of DMSO, “DMSO high” was included as an additional vehicle control setting. Experiment B included ARP2/3 inhibitors and antipsychotics, two classes of compounds reported to reduce cellular migration due to altered actin cytoskeleton dynamics [27]: ARP2/3 inhibitors, CK869, CK666 [28] and benoproperine [29]. These compounds were administered at 5 and 10 µM, each. For all tested compounds and concentrations, cell viability assays were performed to exclude potential bias on migration effects due to cell death (S7 Fig).

The subsequent computational analysis included movie generation, data quality control, cell segmentation, and cell tracking, as described in the Methods section. Together, experiments A and B generated two structured datasets representative of high-throughput cell migration assays. The datasets were exported as a spreadsheet, describing the observed velocity of *n* cells (rows) and their experimental features (chemical compound, dose, plate identifier, well identifier), which was used as main input by *cellmig* (version 1.3.4) to fit a hierarchical Bayesian model implemented in Stan [30]. Using default parameters, *cellmig* performed model inference (S1 Text), including model validation based on posterior predictive checks at both the individual-cell level and the mean velocity per well level (S11 and S12 Figs, respectively). The posterior distributions of the model parameters were then used to quantify treatment effects, including their associated uncertainties, as detailed in the following sections.

### Overall treatment effects

**Fig 3:**
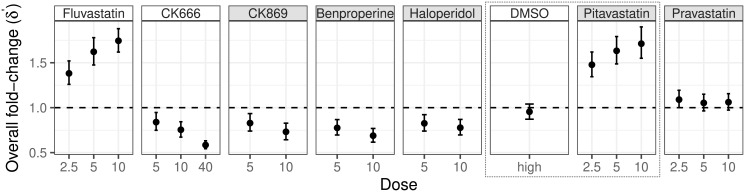
Overall treatment effects on cell migration velocity. Mean (dot) and 95% highest density interval (HDI; error bars) of the overall treatment effects ($\delta^{\prime }$), defined as combinations of compound and dose. Horizontal dashed line indicates the null effect ($\delta^{\prime }$=1, no change). Control compounds (DMSO, CK666, and fluvastatin) have white panels. The dotted gray rectangle encloses results for pitavastatin (at each concentration) and the corresponding control (“DMSO high”).

Dataset 1 was analyzed using *cellmig*, with DMSO as the reference for batch correction (Methods). The model reported overall treatment effects ($\delta_{t}$) as log-fold-changes relative to DMSO, which were exponentiated for interpretation (Fig 3). We refer to these exponentiated estimates as $\delta_{t}^{\prime }$ (fold-change). On the log-scale, the null effect (no change relative to DMSO) corresponds to $\delta_{t}=0$; on the natural fold-change scale, the null effect corresponds to $\delta_{t}^{\prime }=1$. Values greater than 1 indicate increased migration velocity relative to DMSO, while values less than 1 indicate decreased migration velocity.

As expected, high-dose fluvastatin (10 µM) and CK666 (40 µM) showed strong positive ($\delta_{t}^{\prime }$=1.73, 95% HDI [1.61, 1.86]) and negative ($\delta_{t}^{\prime }$=0.59, 95% HDI [0.55, 0.63]) effects, respectively. Dose-response relationships were evident for control compounds: fluvastatin effects decreased to $\delta_{t}^{\prime }$=1.60, 95% HDI [1.46, 1.75] and $\delta_{t}^{\prime }$=1.37, 95% HDI [1.25, 1.50] at 5 µM and 2.5 µM, respectively, while CK666 effects decreased to $\delta_{t}^{\prime }$=0.76, 95% HDI [0.68, 0.85] and $\delta_{t}^{\prime }$=0.85, 95% HDI [0.76, 0.94] at 10 µM and 5 µM, respectively. High-dose DMSO caused a negligible velocity reduction ($\delta_{t}^{\prime }$=0.95, 95% HDI [0.87, 1.03]) with 95% HDIs overlapping the null effect ($\delta^{\prime }$=1).

Among the compounds, pitavastatin (2.5 -10 µM) increased migration ($\delta_{t}^{\prime }$=1.46-1.68), whereas CK869 ($\delta_{t}^{\prime }$=0.74-0.83), benproperine ($\delta_{t}^{\prime }$=0.7-0.78), and haloperidol ($\delta_{t}^{\prime }$=0.78-0.83) reduced migration at 5 and 10 µM. We observed dose-dependent responses in these treatment groups as well. In contrast, pravastatin showed negligible effects across all doses ($\delta_{t}^{\prime }$=1.05-1.08) with 95% HDIs overlapping the null effect ($\delta^{\prime }$=1).

### Quantifying non-treatment contributions to variation

**Fig 4:**
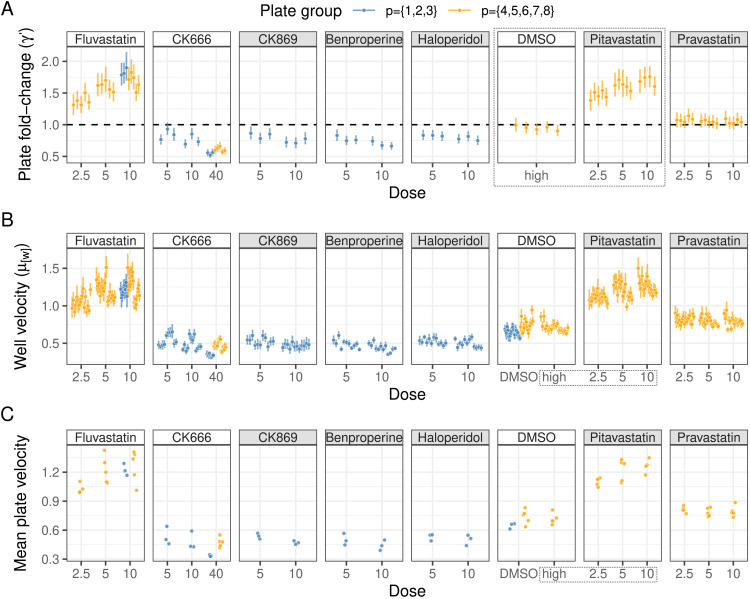
Observations and multilevel treatment effects on cell migration velocity. **(A)** Mean (dot) and 95% highest density interval (HDI; error bars) of the plate-specific treatment effects ($\gamma^{\prime }$). **(B)** Inferred mean (dot) and 95% HDI (error bars) of the cell migration velocity in individual wells ($\mu_{\left[w\right]}$) before correction for plate-effects. **(C)** Observed mean (over all cells) treatment-specific velocities on each plate. Blue and orange dots and error bars in panels A-C correspond to plates from two different plate groups. Horizontal dashed line indicates the null effect ($\gamma^{\prime }$=1, no change). Control compounds (DMSO, CK666, and fluvastatin) have white panels. The dotted gray rectangles mark pitavastatin (at each concentration) and the corresponding control (“DMSO high”).

*cellmig* further quantified plate-specific treatment effects ($\gamma_{pt}$ for treatment *t* on plate *p*; Fig 4A) relative to DMSO. As before, exponen*t*iating $\gamma_{pt}$ yielded interpretable fold-changes $\gamma_{pt}^{\prime }$ (vertical axis of Fig 4A). While $\gamma_{pt}^{\prime }$ values generally mirrored the overarching $\delta_{t}^{\prime }$ estimates, their variability across plates modeled by parameter $\sigma_{\text{bio}}$ (S13B Fig) accounted for biological heterogeneity in treatment responses. To assess batch correction efficacy, we compared $\gamma_{pt}^{\prime }$ distributions for shared treatments (e.g., fluvastatin at 10 µM) against raw plate-specific mean velocities (Fig 4C). Despite systematic baseline differences between experiments – cells in experiment A showed lower raw velocities than those in experiment B (Fig 4C: blue vs. orange dots) – $\gamma_{pt}^{\prime }$ coefficients for shared treatments converged to similar values after correction (Fig 4A: blue vs. orange dots). In fact, the model parameters $\alpha_{p}$ accurately captured these inter-experiment biases (S13A Fig): mean baseline velocities under DMSO were about 0.6-0.66 µm/min (experiment A) vs. 0.72-0.8 µm/min (experiment B).

Using the lowest-level model parameters $\mu_{pt[w]}$, representing uncorrected well-specific mean velocities, we quantified technical variability across replicates (Fig 4C). The distribution of $\mu_{pt[w]}$ estimates and their 95% HDIs revealed well-to-well noise, modeled by parameter $\sigma_{\text{tech}}$ (S13B Fig). This granular quantification enables not only accurate evaluation of treatment effects on migration velocities but it also facilitates the design of cell-migration experiments, as demonstrated below.

### Quantitative comparison of treatment effects

Our model quantifies treatment effects ($\delta_{t}$) relative to DMSO controls while enabling direct inter-treatment comparisons (e.g., between treatments *i* and *j*) through two key metrics: (1) fold change coefficients ($\rho_{ij}^{\prime })$; (2) probabilities ($\pi_{ij}$) quantifying evidence for differential treatment effects.

**Fig 5:**
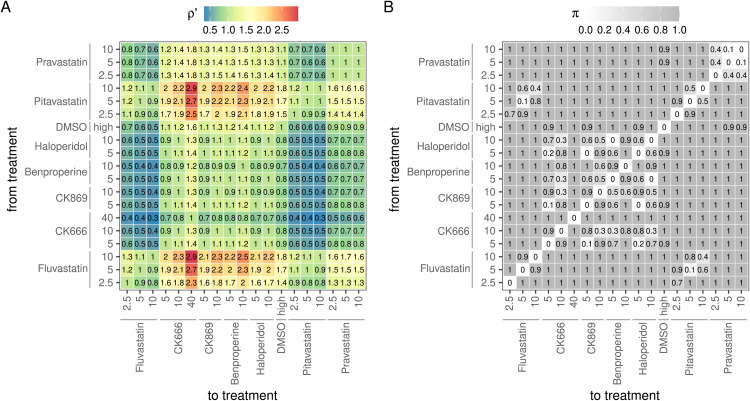
Differences in overall treatment effects. **(A)** Fold change (FC; described by parameter $\rho_{ij}^{\prime }$) between overall treatments effects ($\delta_{t}$) of row (*i*) vs. column (*j*) treatment groups. Tile colors and labels represent $\rho_{ij}^{\prime }$ values (rounded to nearest tenth). **(B)** Probability of differential treatment effect described by parameter $\pi_{ij}$. Tile colors and labels represent $\pi_{ij}$ values (rounded to nearest tenth).

Notably, pitavastatin (2.5 -10 µM) increased migration by $\rho^{\prime }$=1.75-2.42-fold compared to benproperine, CK869, and haloperidol (5–10 µM; red tiles, Fig 5A). These comparisons showed maximal probability (π=1; gray tiles, Fig 5B), indicating strong evidence for differential effects. In contrast, pravastatin (2.5 -10 µM) showed weaker increase ($\rho^{\prime }$=1.25-1.55-fold vs. suppressors; yellow tiles, Fig 5A), consistent with its negligible effect relative to DMSO. The velocity-suppressing compounds – benproperine, CK869, and haloperidol – showed comparable effects across doses ($\rho^{\prime }$=0.84-1.2-fold) with limited evidence for differential activity (π<1). As expected, comparisons of treatment groups with themselves yielded $\rho^{\prime }$ = 1 (no change) and π = 0, i.e., no difference in treatment effects (diagonal entries, Fig 5A–5B).

### Evaluating *cellmig* against commonly used approaches

Cell migration data are most often analyzed with nonparametric tests [9,12,14–17], such as the Wilcoxon rank-sum [31] for pairwise, and the Kruskal-Wallis [32] for multi-group comparisons. These methods represent the current standard practice in the field, despite being originally developed for simpler experimental designs. Given our 19 treatment groups (from Dataset 1), we benchmarked *cellmig* against two alternative approaches: (1) the Kruskal-Wallis test followed by Dunn’s post hoc z-scores and *p*-values for 171 pairwise comparisons, with Benjamini-Hochberg FDR correction. We refer to this entire workflow as the H-test; and (2) a simplified Bayesian model that ignores the nested experimental design and batch effects (S2 and S3 Texts).

#### Comparison with frequentist methods (H-test).

**Fig 6:**
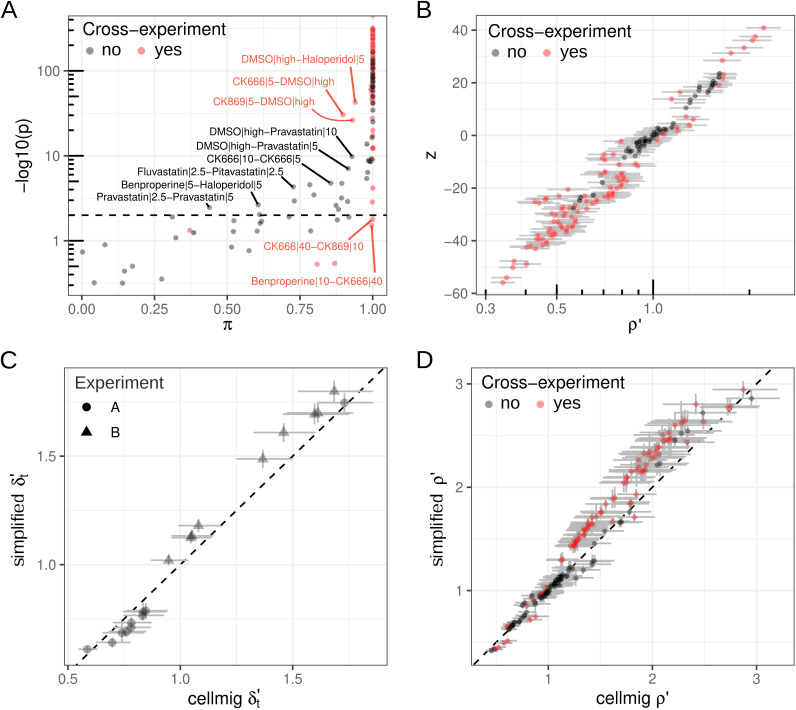
Benchmarking *cellmig* against the H-test and a simplified Bayesian model. (A) *cellmig* probability of differential effect (π) vs. H-test adjusted *p*-values ($-\log_{10}$) for 171 treatment pairs. Labeled points show diverging results. Dashed lines: π=0.99 and adjusted *p* = 0.01. (B) *cellmig* fold-change ($\rho^{\prime }$) vs. H-test *z*-scores. Black/red: intra-/inter-experiment pairs. $\rho^{\prime }$ on log_10_ scale. (C) *cellmig* vs. simplified model overall treatment effects ($\delta_{t}^{\prime }$) with 95% HDIs. Triangles/circles: treatments from Experiment A/B. Dashed line: identity. (D) *cellmig* vs. simplified model pairwise effects ($\rho^{\prime }$). Red dots above diagonal: inter-experiment pairs with inflated effects due to unmodeled batch effects in simplified model.

To mimic common workflows assuming minimal variability and negligible batch effects, we pooled cells by treatment for the H-test, inflating sample sizes but ignoring experimental hierarchy–an issue avoided by *cellmig*. This revealed systematic discrepancies between H-test outputs (*p*-values, *z*-scores) and *cellmig*’s probabilistic metrics (π, ρ) (Fig 6A–6B).

We note that the probability of differential effect (π) and frequentist *p*-values are fundamentally different quantities with different interpretations. Our benchmark is not intended to equate these metrics, but rather to compare the decision-making frameworks that researchers use in practice. In large-scale screens (with >100 treatments), experimental biologists must prioritize a subset of compounds for experimental validation. The current standard practice is to use frequentist methods with a *p*-value threshold (e.g., *p* < 0.05) to declare effects “significant.” Our Bayesian approach provides an alternative decision-making framework based on π. Here, we evaluate how each approach would guide experimental decisions–specifically, which treatments each method would prioritize for follow-up–and demonstrate that π-based decisions are more reliable in the presence of hierarchical structure and batch effects.

**Discordant calls between H-test and *cellmig*:** Two cross-experiment pairs (e.g., benproperine (10 µM) vs. CK666 (40 µM) showed non-significant adjusted *p*-values (*p* > 0.01) in H-test despite strong evidence from *cellmig* (π≥ 0.99) (Fig 6A, bottom-right). All involved batch effects between experiments A/B, where systematic velocity differences masked true treatment effects (Fig 4). Because the H-test pools cells across plates, it conflates biological treatment effects with plate-specific batch offsets. *cellmig*’s hierarchical structure separates these variance components, allowing detection of effects consistent across biological replicates despite batch shifts.

Meanwhile, seventeen pairs (e.g., haloperidol (5 µM) vs. DMSO (high); pravastatin (10 µM) vs. DMSO (high)) showed significant adjusted *p*-values (p≤ 0.01) but low π values (π< 0.99). While some of the significant differences based on the H-test arose from unmodeled batch effects (e.g., cross-experiment pairs), others involved treatment groups from the same experiment. These discrepancies stemmed from two factors: (1) Pooling cells amplified apparent statistical power (green crosses vs. black dots in S14 Fig), rendering small effect sizes significant–a known challenge in large migration datasets [16]; (2) Applying the H-test to individual plates revealed fluctuating *p*-values (e.g., on log10-scale 0.8-4.7 for CK666 (10 µM) vs. haloperidol (10 µM); 0.6-7.5 for fluvastatin (2.5 µM) vs. pitavastatin (5 µM)), reflecting non-negligible biological variability which is ignored by the H-test (S15 Fig). These inconsistent results, where some plates show significant *p*-values while others do not, highlight a key problem of the H-test, namely the interpretation of fluctuating *p*-values on individual plates.

In contrast, *cellmig* explicitly models biological variability, resulting in wider posterior distributions for overall treatment effects ($\delta_{t}^{\prime }$). For example: haloperidol (5 µM) vs. DMSO (high) had overlapping $\delta_{t}^{\prime }$ 95% HDIs: $\delta_{t}^{\prime }$=0.83, 95% HDI [0.75, 0.92] vs. $\delta_{t}^{\prime }$=0.95, 95% HDI [0.87, 1.03]. Hence, the resulting $\rho^{\prime }$=0.88, 95% HDI [0.77, 1.01] included 1 (the null effect) with π=0.94. We obtained similar results for pravastatin (10 µM) vs. DMSO (high).

**Result robustness and interpretability:** *cellmig* reports effect sizes ($\rho_{ij}^{\prime }$) with uncertainties and evidence probabilities ($\pi_{ij}$), while H-test relies on point estimates (*z*-scores, *p*-values). The *z*-score’s sensitivity to sample size amplifies trivial differences in large datasets (Fig 6B) and yields inconsistent interpretations across experiments with varying sample sizes. This fundamentally limits biological insight, as identical effect magnitudes may show divergent statistical significance. *cellmig* avoids this pitfall: its effect sizes remain stable across sample sizes, with the availability of more data improving precision rather than inflating significance.

#### Comparison with simplified Bayesian model.

To explicitly validate the necessity of modeling the full nested experimental structure and batch effects, we compared *cellmig*’s hierarchical model against a simplified Bayesian counterpart that ignored both the nested experimental design (cells within wells within plates) and plate-specific batch effects. While both models yielded similar posterior means for overall treatment effects ($\delta_{t}^{\prime }$), the uncertainty estimates diverged substantially (Fig 6C). The simplified model produced artificially narrow 95% HDIs due to pseudoreplication (pooling all cells receiving the same treatment) and the inability to account for plate-to-plate variability. Furthermore, we observed systematic shifts in treatment effects corresponding to experiments A and B, reflecting unmodeled batch effects (most triangles/circles in Fig 6C shifted above/below the diagonal).

The underestimation of uncertainty propagated to pairwise comparisons (Fig 6D), generating spurious differences between treatment groups. The simplified model identified 30 additional treatment pairs with high probability (π≥ 0.99) of differential effect compared to *cellmig*’s hierarchical model, the majority of which (27) occurred between treatments from the same experiment (S16 Fig). For example, the simplified model detected a significant difference between pravastatin doses (5 µM vs. 2.5 µM: $\rho^{\prime }$ = 0.95, 95% HDI = [0.93, 0.98], π = 1), despite the hierarchical model finding low evidence for differential effects ($\rho^{\prime }$ = 0.97, 95% HDI = [0.86, 1.07], π = 0.44). Additionally, unmodeled batch effects created systematic discrepancies in effect magnitude between the two models, visible as red dots above the diagonal in Fig 6D (inter-experiment comparisons). This highlights the importance of plate-specific intercepts ($\alpha_{p}$) to correct for systematic batch effects, thereby separating technical bias from biological signal.

Leave-One-Out Cross-Validation (LOO-CV) assessed the predictive performance of both models by estimating their ability to generalize to unseen data [33]. We compared the Expected Log Pointwise Predictive Density (ELPD), where higher values indicate better predictive accuracy, and the LOO Information Criterion (LOOIC = −2· ELPD), where lower values are preferred. The hierarchical model achieved an ELPD_LOO_ of -6113.9 (standard error (SE) = 146.7), compared to -6972.5 (SE = 145.9) for the simplified model. This corresponds to an ELPD difference of 858.6 (SE = 45.9) in favor of the hierarchical model, and a lower LOOIC (12227.9, SE = 293.4 vs. 13945.0, SE = 291.9). Although the hierarchical model required more effective parameters (*p*_loo_ = 504.8 vs. 40.2) to account for the nested structure, the substantial gain in predictive accuracy indicates the added complexity is warranted. These results demonstrate that accounting for data hierarchy and batch structure is critical to avoid false positives and ensure reliable uncertainty quantification in high-throughput migration assays.

### Experiment design by numerical simulation

Using parameter values similar to those inferred from our experimental data, including a synthetic vector of effect sizes ($\delta_{t}^{\prime }$) with values range from 0.5 to 2.0, we simulated cell velocity datasets while systematically varying three experimental dimensions: (1) *N*_cells_ = 25, 50, or 100 cells per well; (2) *N*_tech_ = 3, 6, 9, or 12 technical replicates; and (3) *N*_bio_ = 3, 6, 9, or 12 biological replicates per experiment. For each configuration (*N*_cells_, *N*_tech_, *N*_bio_), we generated 300 synthetic datasets and analyzed them using *cellmig* (S4 Text). We then evaluated the widths (*W*s) of the resulting 95% HDIs for $\delta_{t}^{\prime }$ and evaluated their rate of overlap with synthetic effect sizes but not the null effect (true positive rate, TPR). Reliability of the *W* and TPR estimates was assessed by bootstrapping.

#### Biological replicates dominate uncertainty reduction.

**Fig 7:**
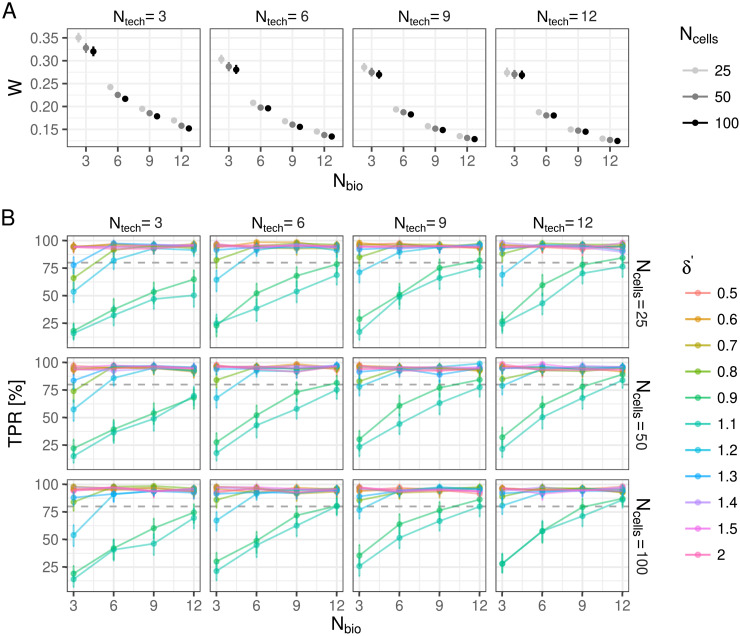
Power analysis. **(A)** Mean widths (*W*) of 95% Highest Density Intervals (HDIs) for effect sizes ($\delta_{t}^{\prime }$, fold changes) for different experimental configurations. Dots are mean *W* of 1,000 bootstraps. Error bars are 95% HDIs of bootstrapped ***W*.** Panels correspond to technical replicates (*N*_tech_). Colors represent cells per well (*N*_cells_), and x-axes show biological replicates (*N*_bio_). **(B)** True positive rates (TPR, %) for effect sizes ($\delta_{t}^{\prime }$). Lines connect TPR values (color-coded by $\delta_{t}^{\prime }$) across *N*_bio_. Grid columns = *N*_tech_, rows = *N*_cells_. Dots are mean TPR values of 1,000 bootstraps, error bars are 95% HDIs of bootstrapped *TPR* values. Horizontal dashed lines indicate 80% TPR. Simulations used values of $\sigma_{\text{bio}}$ and $\sigma_{\text{tech}}$ inferred from Dataset 1. Users should adjust these parameters to reflect the noise structure of their own experimental setups.

First we investigated the impact of experimental configuration (*N*_cells_, *N*_tech_, *N*_bio_) on *W* (Fig 7A). While increasing any of these three sample size dimension reduced *W*, the most substantial reduction occurred with increasing biological replicates (*N*_bio_).

Specifically, setting *N*_bio_ to 3, 6, 9, and 12, was associated with consistently smaller *W* (Fig 7A) from *W* = 0.35 to *W* = 0.12, i.e., increasing *N*_bio_ to 6, 9, and 12 reduced *W* by factors of about 1.45, 1.8, and 2.1, respectively, relative to *N*_bio_ = 3 (S17A Fig). Increasing *N*_tech_ shifted *W* to smaller values (panels in Fig 7A), however, we saw diminishing returns: *W* was reduced by a factor of 1.25 for *N*_tech_ = 12 vs. *N*_tech_ = 3 (S17B Fig). Cell counts per well had negligible impact, with *N*_cells_ = 50 and 100 reducing *W* by less than 1.1-fold versus *N*_cells_ = 25 (S17C Fig).

In summary, experimental designs should therefore prioritize biological replicates for minimizing uncertainty in $\delta_{t}^{\prime }$. Technical replicates showed limited impact in these simulations, consistent with our assumption of low technical variability based on empirical data. We note that *cellmig* enables power analysis under diverse experimental conditions (e.g., high variability regimes), where technical replicates may have greater influence on *W*. Consequently, while biological replicates dominated uncertainty reduction in our specific dataset, the relative importance of technical vs. biological replication may shift in assays with higher technical noise. *cellmig* allows users to explore these trade-offs by adjusting variance parameters to match their specific experimental conditions.

#### Controlling the number of true positives.

A narrow uncertainty interval is only valuable if it actually contains the true effect size. We quantified whether an uncertainty interval contains the true effect size (here synthetic fold-changes $\delta_{t}^{\prime }$), but not the null effect ($\delta_{t}^{\prime }$=1). If it does, we call this a true positive. Otherwise, if the uncertainty interval contains the null effect, we call this a false negative. From 300 simulations performed with the same experimental configuration we computed the TPR (Fig 7B).

Large effects (e.g., migration enhancing $\delta_{t}^{\prime }$=2, 1.5 and 1.4; or migration reducing $\delta_{t}^{\prime }$ = 0.5, 0.6 and 0.7) achieved high TPR ≥90% across all designs. Smaller effects (e.g., $\delta_{t}^{\prime }$ = 0.8 or $\delta_{t}^{\prime }$ = 1.3 and 1.2) required at least 6 biological replicates for TPR ≥80%. For the smallest effects in our simulation ($\delta_{t}^{\prime }=0.9$ or 1.1), maximum number of biological replication (*N*_bio_ = 12) combined with at least 6 technical replicates yielded TPR close to 80%. This demonstrates that detecting subtle treatment effects ($\delta_{t}^{\prime }$ close to 1) requires substantial biological replication.

#### Uncertainty interval calibration.

A critical requirement for uncertainty-aware methods is that reported intervals must be statistically calibrated, containing the true effect at the expected rate (e.g., 95% for a 95% HDI). We reused the simulated data with the same true effect sizes to assess whether *cellmig*’s 95% HDIs contained the true simulated effect. The observed coverage was 95.1% across all simulations and experimental configurations (varying *N*_cells_, *N*_tech_, *N*_bio_). This confirms *cellmig* produces well-calibrated uncertainty estimates.

### Computational requirements

Computational resources required for *cellmig* analyses are determined by two factors: (1) the experimental configuration (number of treatments, biological replicates, and technical replicates) and (2) the No-U-Turn sampler (NUTS) configuration in Stan (number of iterations and chains). We characterized the impact of experimental configuration through systematic simulations using *cellmig*’s *partially* generative mode, while NUTS configuration effects follow directly from the number of iterations and chains. We simulated velocity datasets with *N*_cells_ = 80 cells per well (similar to the mean number of cells per well in Dataset 1), while systematically varying three experimental dimensions: (1) *N*_tech_ = 3, 6, 9, or 12 technical replicates; (2) *N*_bio_ = 3, 6, 9, or 12 biological replicates per experiment; and (3) *T* = 25, 50, or 100 treatments (drugs). For each configuration (*N*_tech_, *N*_bio_, *T*), we generated 3 synthetic datasets and analyzed them using *cellmig* (total of 144 model fits) using the same parameters used for the analysis of Dataset 1 (S1 Text). Benchmarks reflect a single Markov chain run on an AMD EPYC 75F3 workstation. Since we recommend running at least two chains in practice to assess convergence, users should note that parallel execution doubles RAM requirements but does not increase elapsed time.

In our hierarchical design, the total number of model parameters scales approximately as $T\times N_{\text{bio}}\times N_{\text{tech}}$, where well-level parameters dominate the parameter count. Specifically, for screens with comparable size as our experimental datasets (25–50 treatments, 3–6 biological replicates, 3–6 technical replicates, containing up to 1,800 wells with approximately 144,000 cells), model fitting completed in 3–40 minutes with peak RAM usage below 7 GB (S18 Fig). For reference, Dataset 1 contained 20 treatment groups, 8 biological replicates, and 4 technical replicates (338 wells, 29,503 cells), and completed in approximately 15 minutes. Dataset 2 contained 80 treatment groups, 18 biological replicates, and 3–4 technical replicates (1,109 wells, 84,122 cells), and completed in approximately one hour. Large-scale screens beyond our experimental datasets (100 treatments, 12 biological replicates, 12 technical replicates, containing up to 14,400 wells with over 1 million cells) required approximately 8–9 hours and up to 55 GB RAM. Increasing *T* from 25 to 100 treatments increased elapsed time by factors of 5–6 and RAM by factors of 4, while increasing *N*_bio_ from 3 to 12 increased elapsed time by factors of 10–15 and RAM by factors of 8–10 (S18 Fig). Technical replicates showed similar scaling, with *N*_tech_ = 12 vs. *N*_tech_ = 3 increasing elapsed time by factors of 10–15 and RAM by factors of 8–10.

For NUTS configuration, elapsed time scales proportionally with the number of iterations, while peak RAM increases proportionally with iterations (to store posterior samples) and multiplicatively with the number of chains; parallel execution increases RAM but not elapsed time. In summary, *cellmig* is computationally accessible on standard personal computers for typical experimental designs. Most screens with 25–50 treatments and 3–6 replicates complete within 40 minutes using less than 7 GB RAM per chain. For larger screens, we recommend access to a server or high-memory workstation.

### Application of *cellmig* in chemical biology

To validate *cellmig* in an independent application that demonstrates its superior performance and discovers new chemical biology, we set out to compare 15 newly synthesized chemical derivatives of the “parent” compound pimozide for their effect on cell migration. Previously, pimozide, an anti-psychotic drug used in treatment of schizophrenia, was discovered to reduce migration of pancreatic cancer cells via binding to Actin-related protein 2/3 complex subunit 2 (ARPC2), a subunit of the Actin Related Protein 2/3 (ARP2/3) complex [34], similar to the ARP2/3 inhibitors and antipsychotics in our setup experiment B (Dataset 1). Another study attributed its effect on cell migration to the inhibition of dopamine receptor D2 (DRD2) [35]. The aim was to test whether pimozide’s effect on cell migration can be quantitatively analyzed with *cellmig*. Furthermore, we wanted to test whether changes in the chemical structure of pimozide enhance or decrease its effect on cell migration and more importantly, which residues are required to maintain its activity.

This large-scale screen comprised approximately 80,000 cells across 80 compound-dose combinations and in at least three independent biological replicates (S1 Fig). The model used priors established during benchmarking on Dataset 1 (Methods), which proved consistent with Dataset 2 through posterior-prior agreement (S19A–S19B Fig). Furthermore, it accurately captured between-plate batch effects (S19B Fig) and demonstrated validity by accurately reproducing the observed data (posterior predictive checks; S19C Fig).

**Fig 8:**
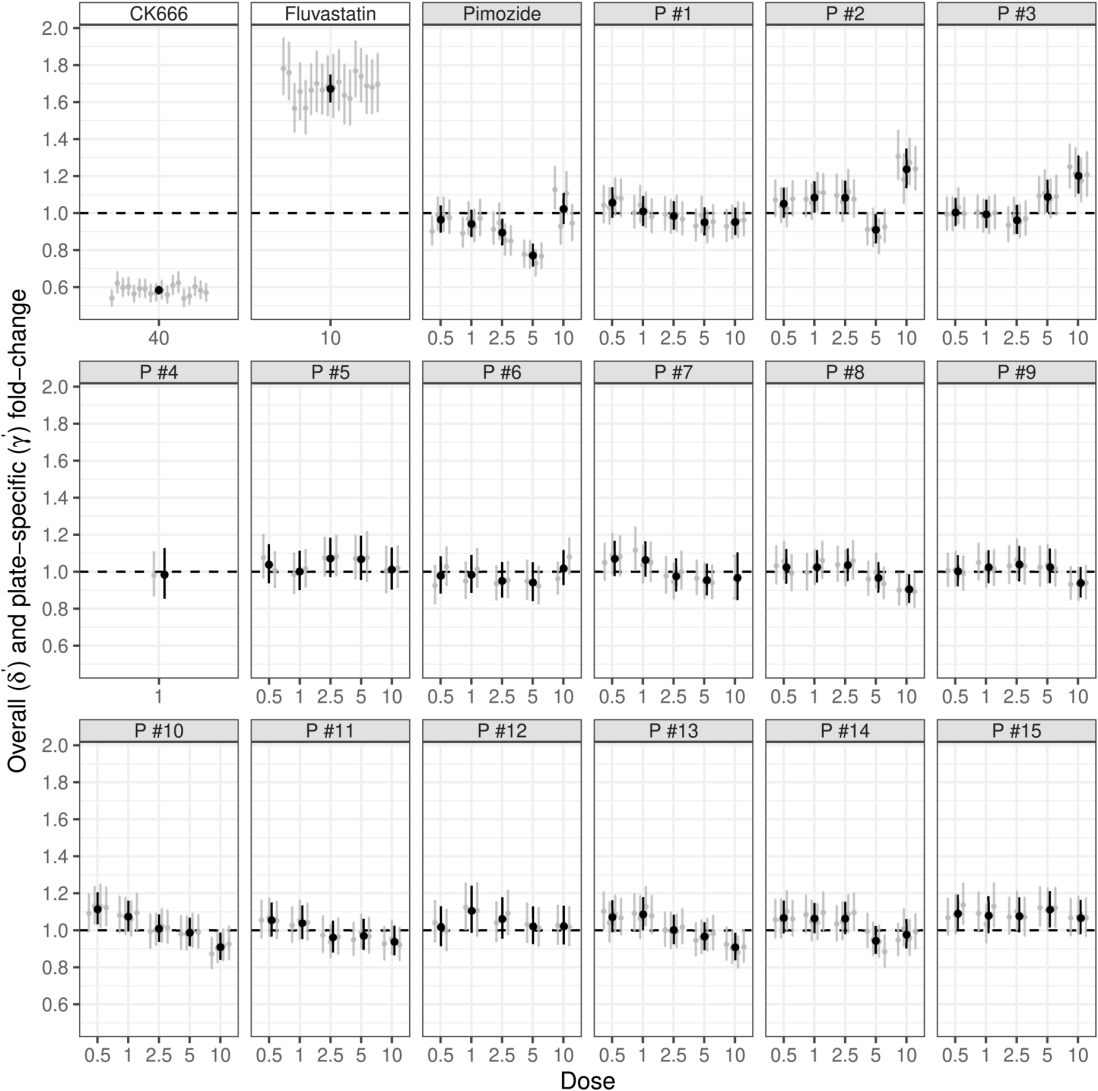
Dose–response analysis of overall ($\delta^{\prime }$) and plate-specific ($\gamma^{\prime }$) treatment effects on cell migration velocity for compounds (panels) in Dataset 2 (application screen). Means (dots) and 95% highest density intervals (HDIs; error bars) are shown for overall treatment effects ($\delta^{\prime }$, black) and plate-specific treatment effects ($\gamma^{\prime }$, gray), defined as combinations of compound and dose. The horizontal dashed line indicates the null effect. Control compounds (CK666 and fluvastatin) are shown in white panels.

The model quantified overall ($\delta_{t}^{\prime }$) and plate-specific ($\gamma_{pt}^{\prime }$) velocity effects as fold-changes relative to DMSO controls, with 95% HDIs capturing the uncertainty in these estimates (Fig 8). Control compounds showed expected responses: CK666 at 40 µM reduced migration velocity ($\delta_{t}^{\prime }$=0.58, 95% HDI [0.56, 0.61]), while fluvastatin at 10 µM enhanced migration velocity ($\delta_{t}^{\prime }$=1.67, 95% HDI [1.6, 1.75]). These velocity effect sizes overlap the $\delta_{t}^{\prime }$ values for CK666 and fluvastatin from independent experiments in Dataset 1 (Fig 3), demonstrating consistent biological responses across experimental batches.

The parent compound pimozide showed a non-monotonic dose-response relationship: no detectable velocity effect at low (0.51; $\delta_{t}^{\prime }$=0.96 [0.89, 1.04] and 0.94 [0.87, 1.02]) or high (10 µM; $\delta_{t}^{\prime }$=1.02 [0.94, 1.11]) doses (95% HDIs overlapping $\delta^{\prime }$=1), but reduced migration velocity at intermediate doses (2.5 µM: $\delta_{t}^{\prime }$=0.9 [0.83, 0.97]; 5 µM: $\delta_{t}^{\prime }$=0.77 [0.71, 0.84]). This analysis revealed that indeed pimozide has a dose-dependent effect on cell migration, but only up to 5 µM. At 10 µM, we did not observe any reduction effects, which is due to this dose inducing proliferation arrest and most likely cell death (S8 Fig), further reiterating the importance of considering compound effects in cell viability as these might impact their effect on migration.

Pimozide derivatives demonstrated diverse velocity response patterns. P#15 showed modest velocity increases across multiple doses: 0.5 µM ($\delta_{t}^{\prime }$=1.09, 95% HDI [1.00, 1.19]), 1 µM ($\delta_{t}^{\prime }$=1.08, 95% HDI [0.98, 1.18]), 2.5 µM ($\delta_{t}^{\prime }$=1.08, 95% HDI [0.99, 1.18]), 5 µM ($\delta_{t}^{\prime }$=1.11, 95% HDI [1.02, 1.21]), and 10 µM ($\delta_{t}^{\prime }$=1.07, 95% HDI [0.98, 1.16]). However, most HDIs approached the null effect ($\delta_{t}^{\prime }$=1), indicating effects were relatively small.

Several compounds showed non-monotonic velocity responses: P#2 increased velocity at 1 µM ($\delta_{t}^{\prime }$=1.08, 95% HDI [1.00, 1.17]), 2.5 µM ($\delta_{t}^{\prime }$=1.08, 95% HDI [1.00, 1.18]), and 10 µM ($\delta_{t}^{\prime }$=1.24, 95% HDI [1.13, 1.35]) but decreased velocity at 5 µM ($\delta_{t}^{\prime }$=0.91, 95% HDI [0.84, 1.00]). For some of these effects the HDIs approached the null effect ($\delta_{t}^{\prime }$=1). P#10 increased velocity at 0.5 µM ($\delta_{t}^{\prime }$=1.11, 95% HDI [1.03, 1.21]) but decreased at 10 µM ($\delta_{t}^{\prime }$=0.91, 95% HDI [0.84, 0.99]), showing negligible effects at intermediate doses. P#13 increased velocity at 1 µM ($\delta_{t}^{\prime }$=1.09, 95% HDI [1.00, 1.19]) but decreased at 10 µM ($\delta_{t}^{\prime }$=0.91, 95% HDI [0.84, 0.99]), with both effects having HDIs approaching $\delta_{t}^{\prime }$=1. P#3 increased velocity only at high doses: 5 µM ($\delta_{t}^{\prime }$=1.09, 95% HDI [1.00, 1.18]) and 10 µM ($\delta_{t}^{\prime }$=1.20, 95% HDI [1.11, 1.31]), showing negligible effects at low and intermediate doses.

Importantly, the majority of derivatives showed no detectable velocity effects at any tested dose. Nine compounds (P#1, P#5, P#6, P#7, P#9, P#11, P#12, P#14, and P#4 at its tested concentration) consistently showed HDIs overlapping the null effect ($\delta^{\prime }$=1) across all concentrations. Additionally, P#8, showed isolated effect exclusively at 10 µM ($\delta_{t}^{\prime }$=0.91, 95% HDI [0.83, 0.99]).

Overall, the direct structure-activity relationship (SAR) analysis showed that each modification to the parent compound’s chemical residues reduced its ability to inhibit migration, underscoring the structural complexity of this molecule. Although a full chemical characterization lies outside the scope of this study, we demonstrate that *cellmig* can resolve subtle and intricate modulation patterns with quantified uncertainty. As shown in our benchmarking (Dataset 1), such patterns may be missed or misinterpreted by standard approaches that do not account for hierarchical structure and batch effects.

## Discussion

We have developed and validated *cellmig*, a specialized software tool that makes rigorous hierarchical Bayesian analysis accessible for quantifying treatment-specific effects on cell migration velocity in high-throughput imaging assays. Our approach addresses key limitations of current methods by modeling hierarchical experimental designs and integrating sources of technical and biological variation into a unified Bayesian framework, implemented in the free, open-source R package *cellmig*, with extensive vignettes for interdisciplinary researchers.

Existing analytical pipelines in the cell migration field commonly rely on non-parametric tests or descriptive summaries (e.g., S1 Fig) that fail to account for replicate structure, batch effects, or uncertainty propagation. Such methods risk overinterpreting single experiments, conflating technical noise with biological signals, and producing irreproducible results. In contrast, *cellmig* employs a Bayesian hierarchical model to deliver probabilistic estimates of condition-specific effects while explicitly accommodating nested data structures (e.g., plates, wells, cells). This enables statistically robust comparisons across conditions with quantified uncertainty, reducing susceptibility to batch artifacts and improving reproducibility.

We acknowledge that the underlying hierarchical Bayesian framework is well-established and could theoretically be implemented using general-purpose packages like *brms* [18]. However, such tools require users to specify complex model formulas, diagnose convergence, and interpret posterior distributions—tasks that present significant barriers for experimental biologists. *cellmig* removes these barriers by providing a purpose-built interface with sensible defaults, automated diagnostics, and domain-specific visualizations (S5 Text). By encapsulating this expertise, *cellmig* enables researchers to focus on biological interpretation rather than statistical implementation.

Benchmarking against the commonly used H-test (Kruskal-Wallis with Dunn’s post hoc test) demonstrated *cellmig*’s enhanced robustness in resolving discordant calls. By accounting for batch effects and hierarchical structure, *cellmig* reduced likely false discoveries arising from inflated sample sizes and unmodeled biological variability, while recovering treatment effects masked by systematic technical noise. This provides more reliable and interpretable results for downstream prioritization.

The reproducibility of our findings underscores the robustness and resolution of the method. Compounds targeting ARP2/3 complex-mediated actin nucleation – CK666 and CK869 [36] – consistently reduced migratory capacity in a dose-dependent manner. Similarly, benproperine, an inhibitor of the ARPC2 subunit of the ARP2/3 complex, demonstrated a comparable dose-dependent reduction in migration, in line with prior observations [29]. Haloperidol, an antipsychotic drug previously shown to inhibit glioblastoma cell migration [37], also reduced migration in our screen, though to a lesser extent than the other compounds.

We previously reported that fluvastatin enhances pancreatic cancer cell migration by inducing a mesenchymal phenotype [13]. Using *cellmig*, we not only confirmed its potent migration-inducing effect but also demonstrated a clear dose-dependent response mirrored by its analog, pitavastatin – both belonging to the same class of synthetic statins. In contrast, pravastatin, from the class of natural or fungal-derived statins, showed no effect on migration, suggesting that migration modulation may involve additional factors beyond HMG-CoA reductase inhibition.

Most importantly, *cellmig* enabled for the first time the direct and quantitative comparison of two completely independent experimental datasets (Dataset 1 and 2), allowing for a rigorous assessment of compound-specific effects on migration. This capability was demonstrated by consistent velocity effects of control compounds (CK666 and fluvastatin) across both datasets. We further applied *cellmig* in the field of chemical biology to analyze novel pimozide derivatives in Dataset 2, which comprised approximately 80,000 cells across 80 compound-dose combinations. The model successfully quantified subtle and complex modulation patterns, including consistent (e.g., P#15) and non-monotonic dose responses (e.g., P#2, P#10, P#13). While the chemical biology interpretation of this structure-activity relationship analysis lies beyond the scope of this study, the Bayesian framework proved particularly valuable here, as it quantified uncertainty in effect estimates (via 95% HDIs) and prevented overinterpretation of small-magnitude changes where HDIs approached the null value.

Another practical feature of *cellmig* is its built-in simulation capacity. While prior predictive sampling is standard in Bayesian analysis [19], *cellmig* integrates these capabilities into simple functions tailored for both model validation (via *fully* generative simulation) and experimental design optimization (via *partially* generative simulation). This flexibility allows users to balance specificity (using pilot data for power analysis) and generalizability (using vague priors for novel contexts). This feature is particularly valuable for resource-limited studies or large-scale screens, where balancing replicate numbers, batch configurations, and inferential precision is critical.

*cellmig* is well-suited for large-scale chemical screens where researchers must prioritize dozens to thousands of compounds for experimental validation. In such high-dimensional settings, we recommend a decision workflow that considers effect size ($\delta_{t}^{\prime }$ or $\rho^{\prime }$ for pairwise comparisons) and support (π) jointly, implemented through heatmaps for modest screens (<100 treatments, Fig 5) or Bayesian volcano plots for large-scale screens (S5 Fig). Specifically, we suggest examining: (1) the posterior mean fold-change ($\delta_{t}^{\prime }$ or $\rho^{\prime }$) to assess biological effect magnitudes; (2) the 95% HDIs to evaluate effect uncertainty and whether the interval excludes the null effect ($\delta_{t}^{\prime }=1$ or $\rho^{\prime }=1$); and (3) the probability π to quantify evidence strength. By balancing effect size against uncertainty, this framework complements established high-throughput screening metrics such as the Strictly Standardized Mean Difference (SSMD) [22] and Z-factors [23] for prioritizing promising candidates for validation. However, unlike these metrics, which rely on point estimates, *cellmig*’s Bayesian framework propagates uncertainty from the cell level through to the treatment effect, offering a probabilistic basis for ranking treatments that is robust to the nested structure of experimental data.

However, our approach has limitations. First, *cellmig* assumes data adhere to a predefined hierarchical structure (S20 Fig), which, while common in high-throughput imaging, may not fit alternative designs (e.g., simpler studies without technical replicates; or more complex studies including unmodeled confounders). Second, the current implementation focuses on mean migration velocity from single-cell tracking data and is not designed for wound-closure assays or collective-migration workflows. Third, the current implementation focuses on mean migration velocity; while the framework is extensible to other phenotypes (e.g., persistence, directionality), such adaptations require additional methodological development. Fourth, *cellmig* quantifies uncertainty at the statistical inference level (biological/technical variability, batch effects) but does not model measurement uncertainty from upstream segmentation and tracking. This is a deliberate design choice: treating velocities as fixed inputs ensures *cellmig* remains accessible to researchers using diverse imaging workflows and tools, enabling immediate impact without requiring raw image access or prohibitive computational resources. While explicitly modeling image-processing uncertainty would represent a more comprehensive approach, it would substantially increase complexity and limit adoption. Future work may integrate measurement uncertainty models for users requiring this additional layer of rigor.

In conclusion, *cellmig* addresses a critical gap in the analysis of live-cell migration assays. By combining hierarchical Bayesian modeling with tools for simulation and uncertainty quantification, it enhances the rigor, interpretability, and cross-study comparability of high-throughput migration data – advancements essential for both mechanistic research and translational applications such as drug discovery. Our application to a large-scale chemical screen demonstrates its particular value in characterizing complex dose-response relationships and structural dependencies in migration-modulating compounds.

## Supporting information

S1 TextBayesian model inference procedure.MCMC sampling parameters (chains, iterations, warm-up), convergence diagnostics ($\hat{R}$, *N*_eff_), and posterior predictive checks used to validate model fit against observed data.(PDF)

S2 Text Stan code. Stan code for the hierarchical Bayesian model used by default in *cellmig*, as well as the simplified model used for benchmarking purposes.
(PDF)

S3 Text Model definitions. Defines key variables, indices, and mathematical specifications for both the hierarchical and simplified Bayesian models, including likelihood functions, hierarchical structures, and prior distributions.
(PDF)

S4 Text Simulation-based experimental design optimization. Describes the simulation-based framework for experimental design optimization. Includes methodology for generating 300 synthetic datasets per configuration across varying numbers of cells, technical replicates, and biological replicates, along with metrics for uncertainty and statistical power.
(PDF)

S5 Text Functional reference for *cellmig.* Provides a functional reference for the *cellmig* R package (version ≥1.3.4). Organizes exported functions into categories (model fitting, posterior predictive checks, generative simulation, visualization) and includes R code snippets demonstrating typical usage workflows.
(PDF)

S1 Fig Visualization of high-throughput live cell migration data. Single-cell migration velocity (y-axis; µm/min) across plates (panels) in Dataset 2 (application screen). In each panel, wells show compound and dose treatments, with individual cells depicted as dots within violin plots to visualize distribution density. Red horizontal lines indicate well-specific means, with error bars representing ± Standard Error of the Mean (SEM). The dashed black line corresponds to the plate-specific DMSO control mean, used as a baseline for comparison across treatments within plates.
(PDF)

S2 FigRelationship between well-specific cell velocity means and variances, and prior distribution validation.(A) Log–log relationship between variance (y-axis) and mean (x-axis) of well-specific cell velocities in Dataset 1 (left) and Dataset 2 (right). (B) Probability distribution of the empirically observed inverse squared coefficient of variation ($\kappa_{w}=\mu_{w}^{2}/\tau_{w}$) for well-specific cell velocities (Dataset 1: solid red; Dataset 2: dashed red). The black line shows the prior distribution of $\mu_{\kappa }$ ($\mu_{\kappa }\sim Normal(\mu =1.5,\sigma =1.0)$), i.e., the mean of the population of $\log(\kappa_{w})$ ($\log(\kappa_{w})\sim Normal(\mu_{\kappa },\sigma_{\kappa })$). Purple dots indicate inverse squared coefficient of variation in 24 wells with control-treated neutrophils from [9]. The x-axis shows $\kappa_{w}$ on a log10 scale. (C) Probability distributions of log-transformed cell velocities for individual cells (orange) and well-specific means (blue); solid lines indicate Dataset 1 and dashed lines indicate Dataset 2. The black line shows the normal prior distribution for $\alpha_{p}$ ($\alpha_{p}\sim Normal(\mu =-0.5,\sigma =1.0)$). Purple dots indicate mean velocities in 24 wells with control-treated neutrophils from [9]. Generated with *ggplot2* [38] and *patchwork* [39].(PDF)

S3 FigPrior predictive distribution of cell velocities under different prior configurations.Simulated and observed cell velocities (*v*) for 29,503 cells are plotted on the y-axis (simulated) and x-axis (observed in Dataset 1), respectively, with both axes on a log10 scale. Orange contours represent the two-dimensional density of the data. Dark gray horizontal lines indicate the minimum and maximum of observed velocity in Dataset 1. Red labels in each panel shows the percentage of simulated velocities exceeding 100 µm/min (biologically implausible range). (A) Default prior configuration (SD = 1 prior on $\sigma_{\text{bio}},\sigma_{\text{tech}},\sigma_{\delta }\sim {Normal}^{\text{+}}(0,1)$). (B) Increased variability between technical replicates (SD = 3 prior on $\sigma_{\text{tech}}\sim {Normal}^{\text{+}}(0,3)$). (C) Increased variability between biological replicates (SD = 3 prior on $\sigma_{\text{bio}}\sim {Normal}^{\text{+}}(0,3)$). (D) Increased treatment effect variability (SD = 3 prior on $\sigma_{\delta }\sim {Normal}^{\text{+}}(0,3)$). (E) All three scale parameters get wider priors (SD = 3 prior on $\sigma_{\text{bio}},\sigma_{\text{tech}},\sigma_{\delta }\sim {Normal}^{\text{+}}(0,3)$). Panels B–E show that wider priors generate implausible velocities, supporting the default priors in (A). Generated with *ggplot2* and *patchwork*.(PNG)

S4 FigPrior sensitivity analysis.(A) Hierarchical *cellmig* model. Scatter plots faceted by parameter comparing posterior means (points) and 95% Highest Density Intervals (error bars) between default priors ($\sigma_{\text{bio}},\sigma_{\text{tech}},\sigma_{\delta }\sim {Normal}^{\text{+}}(0,1)$, x-axis) and wide priors ($\sigma_{\text{bio}},\sigma_{\text{tech}},\sigma_{\delta }\sim {Normal}^{\text{+}}(0,3)$, y-axis), with the dashed diagonal line representing identity. Parameters include treatment effects ($\delta_{t}$, $\delta_{tp}$), plate intercepts ($\alpha_{p}$), well-specific means (μ) and shape parameters (κ), and variance components. (B) Simplified Bayesian model. Comparison of posterior means and 95% HDIs between default prior ($\sigma_{\delta }\sim {Normal}^{\text{+}}(0,1)$, x-axis) and wide prior ($\sigma_{\delta }\sim {Normal}^{\text{+}}(0,3)$, y-axis) priors for treatment effects ($\delta_{t}$), intercept (α), means (μ), shape parameters (κ), and variance ($\sigma_{\delta }$). Close agreement indicates posterior inferences are robust to prior specification.(PDF)

S5 FigBayesian volcano plots comparing pairwise treatment effects.(A) Dataset 1: Differences in overall treatment effects ($\delta_{t}^{\prime }$) between treatment pairs. Dots represent the mean fold-change ($\rho^{\prime }$, x-axis) with 95% HDI error bars. The y-axis represents the probability (π) of differential effect on migration. Top-priority hits are located in the upper corners: top-left ($\rho^{\prime }<$1, π≥0.99) indicates strong migration suppressors; top-right ($\rho^{\prime }>$1, π≥0.99) indicates strong migration enhancers. Dots near the center ($\rho^{\prime }\approx$1) or bottom (π≈0) represent negligible or uncertain effects. (B) Dataset 2: Same visualization as in (A).(PDF)

S6 Fig Chemical structures of 15 derivatives synthesized from the parent compound pimozide. Corresponding chemical names are listed in S1 Table. The figure was created in ChemDraw Prime (Version 25.0.2.14).
(PDF)

S7 Fig Cell viability assays for Dataset 1. Cell viability assay for (A) fluvastatin (B) CK666 (C) pravastatin (D) pitavastatin (E) haloperidol (F) CK869, and (G) benproperine in ASPC1 cells, tested at indicated concentrations and timepoints. DMSO at the concentration present in the highest compound concentration was used as vehicle only control. Symbols represent mean values, with error bars indicating ± SEM calculated from three independent experiments.
(PNG)

S8 Fig Cell viability assay for pimozide. Cell viability assay for pimozide in ASPC1 cells, tested at indicated concentrations and timepoints. Symbols represent mean values, with error bars indicating ± SEM calculated from three independent experiments.
(PDF)

S9 Fig Cell viability assay for 15 pimozide derivatives. Cell viability assay for 15 pimozide derivatives (panels) in ASPC1 cells, tested at indicated concentrations and timepoints. Symbols represent mean values, with error bars indicating ± SEM calculated from three independent experiments. Chemical names are provided in S1 Table.
(PDF)

S10 Fig Cell segmentation and tracking outputs. Representative outputs from the cell segmentation and tracking pipeline for nine movies (M1-M9). For each indicated field/condition, images are displayed in four columns: raw (R), segmentation (S), raw and segmentation (R + S), and raw, segmentation and tracking (R + S + T) views. The R column shows the original bright-field microscopy image used for analysis. The S column shows the identified cell objects after image segmentation. The R + S column shows the segmented cells overlaid with raw image. The R + S + T column overlays segmentation and tracking results onto the original image, providing a visual summary of cell identification and trajectories. Scale bar = 50 µm. Images were visualized with Napari. Affinity Designer was used for final editing and layout design.
(PDF)

S11 Fig Posterior predictive check of cell velocities. Each panel represents a specific chemical compound applied to wells on individual plates. Panel labels indicate the compound name and the corresponding plate identifier. Within each panel, black violins represent the distribution of observed cell velocities (y-axis), with individual cells shown as black dots. Pink violins show the model’s predicted distribution of cell velocities for the same wells. The x-axis denotes the treatment dose applied to the cells in each well. The close overlap between black and pink violins indicates strong agreement between observed and predicted velocities, suggesting that the model accurately retrodicts the observed data. Generated with *ggplot2*.
(PDF)

S12 Fig Posterior predictive check of mean well velocities. Each dot represents a well, with its observed and predicted mean velocity plotted on the x- and y-axes, respectively. Gray vertical error bars indicate the 95% highest density intervals (HDIs) of the predicted mean velocities. A black dotted diagonal line marks the identity line (x = y); dots lying close to this line indicate good agreement between observed and predicted values, suggesting that the model can retrodict the observed data well. Generated with *ggplot2*.
(PDF)

S13 FigMeans and 95% HDIs of several model *M* parameters inferred based on Dataset 1.(A) $\alpha_{p}$ (left panel) defined as the mean migration velocity (on log-scale) of the control treatment DMSO on each plate, and the exponentiated version (right panel), defined as the mean migration velocity (µm/min). Blue and orange dots and error bars correspond to plates from two different plate groups; (B) $\sigma_{\text{bio}}$ defined as the standard deviation of treatment effects between biological replicates (plates), $\sigma_{\text{tech}}$ defined as the standard deviation of treatment effects between technical replicates (wells on plate), and $\sigma_{\delta }$ defined as the standard deviation of the population of overall treatment effects. (C) mean ($\mu_{\kappa }$) and standard deviation ($\sigma_{\kappa }$) of the normal distribution of well-specific $\log(\kappa_{w})$ parameters.(PDF)

S14 FigStatistical comparison of cell velocity between pairs of treatment groups analyzed on the same plate.Black dots represent comparisons based on cell velocity data from individual plates: y-axis shows the $-\log_{10}$ *p*-values obtained from a post hoc analysis using the Dunn’s test Benjamini-Hochberg FDR correction; x-axis shows the pairs of compared treatment groups. Green crosses represent the same statistical comparisons based on pooled cell velocities across plates. The horizontal dashed line represents *p* = 0.05.(PDF)

S15 FigDiscordant treatment pairs identified between the H-test and *cellmig.*(A) Nine treatment pairs where the H-test indicates significance (adjusted *p*-value < 0.01) but *cellmig* indicates low probability (π<0.99) of differential effect velocity, likely due to unmodeled variability. (B) Two pairs where the H-test indicates non-significance but *cellmig* indicates strong evidence, likely due to batch correction. Dots are cell velocities in each treatment group (x and y). Cell velocities are shown as pooled across plates from experiment A and B (left panels), or within individual plates (middle panels). Mean plate-specific treatment effects and overall treatment effects and their 95% HDIs are shown as gray/red dots and error bars (right panels). Larger dots are group medians.(PDF)

S16 FigComparison of treatment effect detections between *cellmig* and the simplified Bayesian model.Tiles represent pairwise treatment comparisons colored by agreement on the probability of differential effect ($\pi_{ij}$). Orange: both methods detect a difference (π≥0.99). Black: neither method detects a difference (π<0.99). Blue: simplified model detects a difference (π≥0.99) but *cellmig* does not. Diagonal entries are omitted; the matrix is symmetric.(PDF)

S17 FigFold change in 95% HDI length (*W*) of $\delta_{t}^{\prime }$ between pairs of experimental configurations.(A) Fold change in *W* between *N*_bio_ = 3 vs. *N*_bio_ = 6, 9, and 12 for specific combinations of *N*_tech_ and *N*_cells_. (B) Fold change in *W* between *N*_tech_ = 3 vs. *N*_tech_ = 6, 9, and 12 for specific combinations of *N*_bio_ and *N*_cells_. (C) Fold change in *W* between *N*_cells_ = 25 vs. *N*_cells_ = 50 and 100 for specific combinations of *N*_bio_ and *N*_tech_. Dots are mean fold changes of 1,000 bootstraps, error bars are 95% HDIs of bootstrapped fold changes.(PDF)

S18 Fig Computational requirements benchmarking. (A) Elapsed time (hours) and (B) peak random access memory (RAM) usage (gigabytes) for *cellmig* model fitting across varying experimental configurations. Each panel corresponds to a specific combination of biological replicates (*N*_bio_, rows) and technical replicates (*N*_tech_, columns). The number of treatment groups (*N*_group_) was varied from 25 to 100. Gray lines connect results from the same synthetic dataset (*n* = 3 per configuration), while points represent individual model fits. Benchmarks reflect a single Markov chain run on an AMD EPYC 75F3 workstation with *N*_cells_ = 80 cells per well.
(PDF)

S19 FigMeans and 95% HDIs of model *M* parameters inferred based on Dataset 2 and model predictions.(A) Means and 95% HDIs of $\sigma_{\text{bio}}$ defined as the standard deviation of treatment effects between biological replicates (plates), $\sigma_{\text{tech}}$ defined as the standard deviation of treatment effects between technical replicates (wells on plate), and $\sigma_{\delta }$ defined as the standard deviation of the population of overall treatment effects ($\delta_{t}$). Also shown are the mean ($\mu_{\kappa }$) and standard deviation ($\sigma_{\kappa }$) of the normal distribution of well-specific $\log(\kappa_{w})$ parameters. (B) Means and 95% HDIs of $\alpha_{p}$ (left panel) defined as the mean migration velocity (on log-scale) of the control treatment DMSO on each plate, and the exponentiated version (right panel), defined as the mean migration velocity (µm/min). (C) Posterior predictive check of mean well velocities in Dataset 2. Each dot represents a well, with its observed and predicted mean velocities plotted on the x- and y-axes, respectively. Gray vertical error bars indicate the 95% highest density intervals (HDIs) of the predicted mean velocities. A black dotted diagonal line marks the identity line (x = y); dots lying close to this line indicate good agreement between observed and predicted values, suggesting that the model can retrodict the observed data well. Generated with *ggplot2* and *patchwork*.(PDF)

S20 Fig Experimental design. A number of cells are seeded in well *w*, position on 96-well plate *p*. At least 4 wells on a plate are treated with treatment group *t* (chemical compound administered at a specific concentration).
(PDF)

S1 Table Chemical compounds. Identifiers (IDs) and names of chemical compounds.
(PDF)

S2 Table Tracking-error summary based on manual frame correction. Error counts were derived from a representative subset of 9 movies (20 frames each; 180 frames total). Automated tracking results were compared against manually corrected annotations after applying a minimum track-duration filter (≥10 frames per track). The table reports acyclic oriented graphs matching (AOGM)-inspired error counts following the implementation in MMV_H4Tracks (https://github.com/MMV-Lab/mmv_h4tracks), including false-positive detections (fp), false-negative detections (fn), object-splitting operations (sc), edge-deletion operations (de), edge-addition operations (ae), and total number of cell instances (N). For the corresponding sensitivity analysis of the downstream migration readout, 354 tracks were identified before correction and 360 after. The difference in average velocity before and after correction was negligible (difference = 0.003; 95% confidence interval = -0.028, 0.034; p-value = 0.85, Welch Two Sample t-test). Statistics across movies include Average (AVG), Standard Deviation (STD), and Average Error Percentage with Standard Error (SE).
(PDF)

S3 TablePrior hyperparameters for gen_full() and gen_partial().Each prior is specified as a normal distribution N(M,SD) where *M* denotes the mean (_M) and *SD* denotes the standard deviation (_SD). Superscript + indicates half-normal priors (truncated at zero) for scale parameters.(PDF)
