# Supplementary material for: Uncertainty-aware quantitative analysis of high-throughput live cell migration data
Source: PLoS Comput Biol. 2026 Jul 13;22(7):e1014472. doi: 10.1371/journal.pcbi.1014472 (PMC13387618; doi:10.1371/journal.pcbi.1014472)
Supplement: S1 Text — MCMC sampling parameters (chains, iterations, warm-up), convergence diagnostics (R^, Neff), and posterior predictive checks used to validate model fit against observed data. (PDF) [file pcbi.1014472.s001.pdf]

Supplementary information

Model inference and checks

Model inference in *cellmig* (version 1.3.4) was performed using the No-U-Turn sampler in the *rstan* R package (version 2.32.7), with four Markov chains run for 2,000 iterations each, including 1,000 warm-up iterations.

To validate the model, we performed posterior predictive checks to confirm that simulated data were consistent with observed data. For each fitted model, we generated replicated datasets (y^{rep}) by simulating cell velocities from the posterior predictive distribution:

$$p(y^{\text{rep}}|y) = \int p(y^{\text{rep}}|\theta)p(\theta|y)d\theta \tag{1}$$

where θ represents all model parameters and y denotes the full observed dataset. For each posterior sample s, we draw parameter estimates $\theta^{(s)} \sim p(\theta|y)$, which are then used to generate replicated data $y^{\text{rep}(s)}$. This yields a posterior predictive distribution for each observed data point. We assessed whether the observed data points fell within the 95% highest density interval (HDI) of the corresponding posterior predictive distribution.

Specifically, we compared the distribution of simulated velocities (y_i^{rep}) against the observed velocities (y_i) for each cell i to assess whether the Gamma likelihood captures the skewness and heavy tails of individual cell velocities (Supplementary Figure S11). Additionally, we compared the observed mean velocity per well ($\hat{y}_w$) with the inferred well-level mean parameter (μ_w) to evaluate whether the hierarchical structure accurately reflects the observed data (Supplementary Figure S12). Close agreement between y_i^{rep} and y_i, and between $\hat{y}_w$ and μ_w, indicates that the model is well-calibrated and that the resulting HDIs are reliable. Conversely, discrepancies would suggest model misspecification, such as an inappropriate likelihood choice or unmodeled sources of variation.

We evaluated sampling convergence using the potential scale reduction factor ($\hat{R}$), the effective sample size (N_{eff}), and diagnostic information provided by *rstan* (e.g., divergence warnings during MCMC sampling). After having passed these tests, the posterior distributions of the model parameters were used to quantify differences in treatment effects including their uncertainties.
