# Supplementary material for: Uncertainty-aware quantitative analysis of high-throughput live cell migration data
Source: PLoS Comput Biol. 2026 Jul 13;22(7):e1014472. doi: 10.1371/journal.pcbi.1014472 (PMC13387618; doi:10.1371/journal.pcbi.1014472)
Supplement: S2 Text — Stan code for the hierarchical Bayesian model used by default in cellmig, as well as the simplified model used for benchmarking purposes. (PDF) [file pcbi.1014472.s002.pdf]

Supplementary information

Stan Models

Hierarchical Bayesian Model (*cellmig*)

The following Stan code implements the full hierarchical model with plate-specific batch correction, technical and biological variability, and partial pooling of treatment effects. This model is used by default in *cellmig*.

```
data {
  int<lower=0> N;                // number of cells
  int<lower=0> N_well;           // number of wells
  int<lower=0> N_plate;          // number of plates
  int<lower=0> N_plate_group;    // number of plate groups of wells
  int<lower=0> N_group;          // number groups
  vector[N] y;                   // cell velocity for N cells
  int well_id [N];               // well ID
  int plate_id [N_well];         // plate ID
  int plate_group_id [N_well];   // plate group ID
  int group_id [N_plate_group]; // group ID: treatment x dose
  int offset [N_well];           // offset = 1 (use for batch correction)
  // priors
  real prior_alpha_p_M;          // prior mean of alpha_p
  real prior_alpha_p_SD;         // prior SD of alpha_p
  real prior_sigma_bio_M;        // prior mean of sigma_bio
  real prior_sigma_bio_SD;       // prior SD of sigma_bio
  real prior_sigma_tech_M;       // prior mean of sigma_tech
  real prior_sigma_tech_SD;      // prior SD of sigma_tech
  real prior_kappa_mu_M;         // prior mean of kappa_mu
  real prior_kappa_mu_SD;        // prior SD of kappa_mu
  real prior_kappa_sigma_M;      // prior mean of kappa_sigma
  real prior_kappa_sigma_SD;     // prior SD of kappa_sigma
  real prior_sigma_delta_M;      // prior mean of sigma_delta
  real prior_sigma_delta_SD;     // prior SD of sigma_delta
}

parameters {
  vector [N_plate] alpha_p;

  real <lower=0> sigma_bio;
  real <lower=0> sigma_tech;
  real <lower=0> sigma_delta;

  real kappa_mu;
  real <lower=0> kappa_sigma;

  vector [N_well] z_1;
  vector [N_plate_group] z_2;
  vector [N_well] z_3;
  vector [N_group] z_4;
}

transformed parameters {
  vector <lower=0> [N_well] kappa;
  vector<lower=0> [N_well] mu;
  vector [N_well] mu_well;
  vector [N_plate_group] delta_tp;
  vector [N_group] delta_t;
```

```

    delta_t = sigma_delta * z_4;
    delta_tp = delta_t[group_id] + sigma_bio * z_2;
    for(w in 1:N_well) {
      if(offset[w]==1) {
        mu_well[w] = alpha_p[plate_id[w]] + sigma_tech * z_1[w];
      } else {
        mu_well[w] = alpha_p[plate_id[w]] + delta_tp[plate_group_id[w]] + sigma_tech * z_1[w];
      }
    }
    mu = exp(mu_well);
    kappa = exp(kappa_mu + kappa_sigma * z_3);
  }

  model {
    alpha_p ~ normal(prior_alpha_p_M, prior_alpha_p_SD);
    kappa_mu ~ normal(prior_kappa_mu_M, prior_kappa_mu_SD);

    // scales
    sigma_bio ~ normal(prior_sigma_bio_M, prior_sigma_bio_SD);
    sigma_tech ~ normal(prior_sigma_tech_M, prior_sigma_tech_SD);
    kappa_sigma ~ normal(prior_kappa_sigma_M, prior_kappa_sigma_SD);
    sigma_delta ~ normal(prior_sigma_delta_M, prior_sigma_delta_SD);

    z_1 ~ std_normal();
    z_2 ~ std_normal();
    z_3 ~ std_normal();
    z_4 ~ std_normal();

    y ~ gamma(kappa[well_id], kappa[well_id] ./ mu[well_id]);
  }

  generated quantities {
    real y_hat_sample [N_well];
    real log_lik [N];

    y_hat_sample = gamma_rng(kappa, kappa ./ mu);
    for(i in 1:N) {
      log_lik[i] = gamma_lpdf(y[i] – kappa[well_id[i]], kappa[well_id[i]] ./ mu[well_id[i]]);
    }
  }

```

Listing 1: Hierarchical Stan model (cellmig)

Simplified Bayesian Model

The following Stan code implements a simplified model that ignores the nested experimental design and plate-specific batch effects. This model was used for benchmarking purposes only (see Results, “Comparison of *cellmig*’s hierarchical model vs. simplified counterpart”).

```
  data {
    int<lower=0> N;                // number of cells
    int<lower=0> N_group;          // number groups
    vector[N] y;                   // cell velocity for N cells
```

```
  int group_id [N];             // group ID
  int offset [N_group];          // offset = 1 (use for batch correction)
  // priors
  real prior_alpha_p_M;         // prior mean of alpha_p
  real prior_alpha_p_SD;        // prior SD of alpha_p
  real prior_kappa_M;           // prior mean of kappa_mu
  real prior_kappa_SD;          // prior SD of kappa_mu
  real prior_sigma_delta_M;     // prior mean of sigma_delta
  real prior_sigma_delta_SD;    // prior SD of sigma_delta
}

parameters {
  real alpha_p;
  vector <lower=0> [N_group] kappa_exp;
  real <lower=0> sigma_delta;
  vector [N_group] z_1;
  vector [N_group-1] z_4;
}

transformed parameters {
  vector<lower=0> [N_group] mu;
  vector [N_group] mu_exp;
  vector [N_group-1] delta_t;
  vector [N_group] kappa;

  delta_t = sigma_delta * z_4;
  // t=1 control
  for(t in 1:N_group) {
    if(t==1) {
      mu_exp[t] = alpha_p;
    } else {
      mu_exp[t] = alpha_p + delta_t[t-1];
    }
  }
  mu = exp(mu_exp);
  kappa = exp(kappa_exp);
}

model {
  alpha_p ~ normal(prior_alpha_p_M, prior_alpha_p_SD);
  kappa_exp ~ normal(prior_kappa_M, prior_kappa_SD);
  // scales
  sigma_delta ~ normal(prior_sigma_delta_M, prior_sigma_delta_SD);
  z_1 ~ std_normal();
  z_4 ~ std_normal();

  y ~ gamma(kappa[group_id], kappa[group_id] ./ mu[group_id]);
}

generated quantities {
  real y_hat_sample [N_group];
  real log_lik [N];
  y_hat_sample = gamma_rng(kappa, kappa ./ mu);
  for(i in 1:N) {
    log_lik[i] = gamma_lpdf(y[i] — kappa[group_id[i]], kappa[group_id[i]] ./
mu[group_id[i]]);
  }
}
```

Listing 2: simplified Stan model (benchmark)
