# Supplementary material for: Uncertainty-aware quantitative analysis of high-throughput live cell migration data
Source: PLoS Comput Biol. 2026 Jul 13;22(7):e1014472. doi: 10.1371/journal.pcbi.1014472 (PMC13387618; doi:10.1371/journal.pcbi.1014472)
Supplement: S3 Text — Defines key variables, indices, and mathematical specifications for both the hierarchical and simplified Bayesian models, including likelihood functions, hierarchical structures, and prior distributions. (PDF) [file pcbi.1014472.s003.pdf]

Supplementary information

Model Specifications

Key Variables and Indices

Before presenting the model specifications, we define the key variables and indices used throughout:

- $i = 1, \ldots, N$: individual cells
- $w = 1, \ldots, N_{\text{well}}$: wells
- $p = 1, \ldots, N_{\text{plate}}$: plates (biological replicates)
- $t = 1, \ldots, N_{\text{group}}$: treatment groups (compound $\times$ dose combinations)
- y_i: observed migration velocity for cell i
- μ_w: well-specific mean migration velocity (natural scale)
- κ_w: well-specific Gamma shape parameter
- α_p: plate-specific intercept (baseline velocity on log-scale)
- δ_t: overall treatment effect (log-fold-change relative to control)
- δ_{tp}: plate-specific treatment effect for treatment t on plate p
- σ_{tech}: standard deviation for technical variability (well-to-well)
- σ_{bio}: standard deviation for biological variability (plate-to-plate)
- σ_δ: standard deviation for treatment effect pooling
- σ_κ: standard deviation for Gamma shape parameter variability
- μ_κ: population mean of $\log(\kappa_w)$

Hierarchical Bayesian Model (*cellmig*)

Likelihood

$$y_i \sim \text{Gamma}(\kappa_{w[i]}, \kappa_{w[i]}/\mu_{w[i]}) \tag{1}$$

Well-level mean structure

$$\log(\mu_w) \sim \begin{cases} \text{Normal}(\alpha_{p[w]}, \sigma_{\text{tech}}), & \text{if control treatment} \\ \text{Normal}(\alpha_{p[w]} + \delta_{tp[w]}, \sigma_{\text{tech}}), & \text{otherwise} \end{cases} \tag{2}$$

Hierarchical treatment effects

$$\delta_{tp} \sim \text{Normal}(\delta_t, \sigma_{\text{bio}}) \tag{3}$$
$$\delta_t \sim \text{Normal}(0, \sigma_\delta) \tag{4}$$

Gamma shape parameter

$$\log(\kappa_w) \sim \text{Normal}(\mu_\kappa, \sigma_\kappa) \tag{5}$$

Plate-specific batch effects

$$\alpha_p \sim \text{Normal}(-0.5, 1.0) \tag{6}$$

Priors

$$\mu_\kappa \sim \text{Normal}(1.5, 1.0) \tag{7}$$
$$\sigma_{\text{bio}}, \sigma_{\text{tech}}, \sigma_\kappa, \sigma_\delta \sim \text{Normal}^+(0, 1) \tag{8}$$

Simplified Bayesian Model

Likelihood

$$y_i \sim \text{Gamma}(\kappa_{t[i]}, \kappa_{t[i]}/\mu_{t[i]}) \tag{9}$$

Group-level mean structure

$$\log(\mu_t) = \begin{cases} \alpha, & \text{if control treatment} \\ \alpha + \delta_t, & \text{otherwise} \end{cases} \tag{10}$$

Treatment effects

$$\delta_t \sim \text{Normal}(0, \sigma_\delta) \tag{11}$$

Gamma shape parameter

$$\log(\kappa_t) \sim \text{Normal}(1.5, 1.0) \tag{12}$$

Priors

$$\alpha \sim \text{Normal}(-0.5, 1.0) \tag{13}$$
$$\sigma_\delta \sim \text{Normal}^+(0, 1) \tag{14}$$
