# Supplementary material for: Uncertainty-aware quantitative analysis of high-throughput live cell migration data
Source: PLoS Comput Biol. 2026 Jul 13;22(7):e1014472. doi: 10.1371/journal.pcbi.1014472 (PMC13387618; doi:10.1371/journal.pcbi.1014472)
Supplement: S4 Text — Describes the simulation-based framework for experimental design optimization. Includes methodology for generating 300 synthetic datasets per configuration across varying numbers of cells, technical replicates, and biological replicates, along with metrics for uncertainty and statistical power. (PDF) [file pcbi.1014472.s004.pdf]

Supplementary information

Experiment design by numerical simulation

Based on our experimental data, we found that δ'_t values range from 0.5 (indicating a two-fold reduction in cell velocity for treatment group t relative to control) to 2 (indicating a two-fold increase). Using these realistic parameter values, we generated a synthetic vector of treatment effects spanning small to large magnitudes: δ'_t = 0.5, 0.6, 0.7, 0.8, 0.9, 1.0, 1.1, 1.2, 1.3, 1.4, 1.5, 2.0, where the treatment group with index $t = 6$ ($\delta'_t = 1.0$) served as the control (offset). Using δ'_t and additional realistic model parameters from our experiments (e.g., biological replicate variability: σ_{bio}=0.1; technical replicate variability: σ_{tech}=0.1; S13 Fig), we simulated cell velocity datasets while systematically varying three experimental dimensions: (1) N_{cells} = 25, 50, or 100 cells per well; (2) N_{tech} = 3, 6, 9, or 12 wells (technical replicates) per plate; and (3) N_{bio} = 3, 6, 9, or 12 plates (biological replicates) per experiment. For each configuration defined by the tuple (N_{cells}, N_{tech}, N_{bio}), we generated 300 synthetic datasets and analyzed them using *cellmig*.

We then evaluated the widths (Ws) of the resulting 95% HDIs for all δ'_t at a given experimental configuration, and evaluated their overlap status (O) with synthetic effect sizes. If the 95% HDI interval contains the true effect size (here synthetic fold-changes δ'_t), but not the null effect (δ'_t=1), we call this a true positive (O=1). Otherwise, we call this a false negative (O=0). From 300 simulations performed with the same experimental configuration we computed the true positive rate (percentage) as the mean of O: TPR = $\sum_{i=1}^{300}(O_i)/300 * 100$. Reliability of the W and TPR estimates was assessed by the following bootstrapping procedure:

1. For each experimental configuration we performed 1,000 bootstrap iterations

2. In each iteration, we sample 100 W and O values with replacement for a specific experimental configuration and computed their means

3. This gives us for each experimental configuration a distribution of 1,000 mean W and TPR values, that we characterize with its mean and 95% HDI.
