# Supplementary material for: Uncertainty-aware quantitative analysis of high-throughput live cell migration data
Source: PLoS Comput Biol. 2026 Jul 13;22(7):e1014472. doi: 10.1371/journal.pcbi.1014472 (PMC13387618; doi:10.1371/journal.pcbi.1014472)
Supplement: S5 Text — Provides a functional reference for the cellmig R package (version ≥1.3.4). Organizes exported functions into categories (model fitting, posterior predictive checks, generative simulation, visualization) and includes R code snippets demonstrating typical usage workflows. (PDF) [file pcbi.1014472.s005.pdf]

Supplementary information

cellmig functions

cellmig (version ≥1.3.4) is a Bioconductor package that implements Bayesian hierarchical modeling for cell migration velocity analysis. The package is available at https://bioconductor.org/packages/cellmig and provides a complete workflow for analyzing high-throughput cell migration data, from model fitting to visualization and simulation-based experimental design.

The package exports nine functions organized into four categories: (i) model fitting, (ii) posterior predictive checks, (iii) generative simulation, and (iv) visualization and comparison of treatment effects. In this section, we describe the main functions and their purposes. For each function, we provide a brief description followed by a representative R code snippet demonstrating typical usage. All code examples assume the package has been loaded via *cellmig* and that example data has been loaded via `data("d", package = "cellmig")`.

Additional documentation, including complete function reference manuals and extended vignettes, is available through the Bioconductor package page and by running `browseVignettes("cellmig")` in R.

Model fit

`cellmig()` This is the core function of the package. The primary argument, `x`, accepts a `data.frame` where rows represent individual cells and columns represent cell features. Required columns include: well ID, plate ID, treatment compound ID, treatment dose, a control indicator (to account for batch effects), and velocity (μm/min).

The function validates the input data, performs necessary formatting, and fits a hierarchical Bayesian model (implemented in Stan) to the migration velocity data. It computes parameter summaries from the posterior distributions and returns a structured list containing: (i) the fitted `rstan` model object, (ii) the processed input data, (iii) posterior summaries for all model parameters (means, medians, and 95% Highest Density Intervals), and (iv) MCMC control settings.

Key estimated parameters include: δ_t (overall treatment effects relative to control), γ_{pt} (plate-specific treatment effects), α_p (plate-specific batch effects), σ_{bio} (variability between biological replicates), σ_{tech} (variability between technical replicates), and σ_δ (variability between overall treatment effects) Users can configure MCMC sampling parameters and prior distributions through the `control` list argument.

```
# load cellmig
library(cellmig)
# use example cell migration dataset provided by cellmig
```

```
data("d", package = "cellmig")

fit ← cellmig(x = d,
              control = list(mcmc_warmup = 1000,
                             mcmc_steps = 2000,
                             mcmc_chains = 4,
                             mcmc_cores = 4))

# Access posterior summaries
fit$posteriors$delta_t
fit$posteriors$sigma_bio
fit$posteriors$sigma_tech
fit$posteriors$sigma_delta

```

Posterior Predictive Checks

The functions in this category facilitate posterior predictive checks (PPC) to assess model fit and validity. The primary input is the output returned by `cellmig()`, from which they extract posterior draws of simulated cell velocities and compare these against the observed velocities at the well and cell levels.

`get_ppc_means()` This function performs posterior predictive checks by comparing observed versus predicted mean migration velocities per well. It extracts posterior predictive samples from the fitted Stan model. The function returns a `data.frame` containing the observed and simulated mean well velocities, along with associated 95% Highest Density Intervals (HDIs) for the simulated values. Additionally, it generates a scatter plot where each point represents a well, with observed means on the x-axis and predicted means on the y-axis. Error bars represent the 95% HDI, and a diagonal reference line ($y = x$) indicates perfect agreement between observed and predicted values. Points clustered near the diagonal, with observed means falling within the predictive HDIs, indicate good model fit at the well level (S12 Fig).

```
# Generate PPC means plot
ppc_means ← get_ppc_means(fit)
print(ppc_means)

```

`get_ppc_violins()` This function performs posterior predictive checks by comparing observed versus predicted distributions of cell velocities. It extracts posterior predictive samples from the fitted Stan model and overlays them with observed data points, grouped by compound and plate. The resulting plot features dashed red violin plots representing the posterior predictive distributions, while overlaid black Sina plot points represent the observed cell velocities (S11 Fig).

```
# Generate PPC violins plot (faceted by chemical compound and plate)
```

```
ppc_violins ← get_ppc_violins(fit, wrap = TRUE)
print(ppc_violins)

```

Generative Functions

The functions in this category enable generative simulation of cell migration velocity data based on the hierarchical Bayesian model structure. Unlike the posterior predictive check functions, these do not require a fitted model object as input. Instead, they rely on a `control` list to specify experimental design parameters (e.g., number of replicates, cells per well) and model parameter values (either drawn from priors or fixed by the user). These functions are essential for prior predictive checks, power analysis, and optimizing experimental designs before data collection. Both functions return a `data.frame` containing simulated cell migration velocities structured identically to the input data expected by `cellmig()`.

The `gen_full()` and `gen_partial()` functions accept prior hyperparameters through the `control` list argument. These parameters define the prior distributions from which model parameters are drawn during simulation. Supplementary Table ?? summarizes each prior parameter, its role in the hierarchical model, and typical default values.

`gen_full()` This function simulates cell migration velocity data from a *fully generative* hierarchical Bayesian model, where all model parameters are drawn from their prior distributions. The primary input is a `control` list specifying experimental design parameters (number of biological replicates, technical replicates, cells per well, treatment groups) and prior distribution hyperparameters. The function generates data from the prior predictive distribution and returns a `data.frame` of simulated velocities. This is useful for exploring parameter space, validating model behavior under various assumptions, and generating synthetic datasets when no empirical data is available (y-axis of S3 Fig).

```
# Simulate data from fully generative model
sim_full ← gen_full(control = list(
                            N_biorep = 3,
                            N_techrep = 3,
                            N_cell = 50,
                            N_group = 5,
                            prior_alpha_p_M = -0.5,
                            prior_alpha_p_SD = 1.0,
                            prior_kappa_mu_M = 1.5,
                            prior_kappa_mu_SD = 1.0,
                            prior_kappa_sigma_M = 0.0,
                            prior_kappa_sigma_SD = 1.0,
                            prior_sigma_bio_M = 0.0,
                            prior_sigma_bio_SD = 1.0,
                            prior_sigma_tech_M = 0.0,
```

```
                                prior_sigma_tech_SD = 1.0,
                                prior_sigma_delta_M = 0.0,
                                prior_sigma_delta_SD = 1.0))

```

gen_partial() This function simulates cell migration velocity data from a *partially generative* hierarchical Bayesian model, where specific parameters (e.g., treatment effect sizes δ_t, variability parameters σ_{bio} and σ_{tech}) are fixed to user-specified values while remaining parameters are drawn from their prior distributions. The primary input is a `control` list that accepts user-defined δ vectors, σ_{bio}, σ_{tech}, and offset treatment index, alongside experimental design parameters. The function returns a `data.frame` of simulated velocities with known ground truth parameters. This is particularly useful for power analysis, experimental design optimization, and model validation (comparing inferred versus true parameter values), allowing users to determine the number of replicates needed to detect effects of a given magnitude.

```
    # Simulate data from partially generative model
    sim_partial <- gen_partial(control = list(
                          N_biorep = 6,
                          N_techrep = 3,
                          N_cell = 50,
                          delta = c(-0.2, -0.1, 0, 0.1, 0.2),
                          sigma_bio = 0.1,
                          sigma_tech = 0.05,
                          offset = 3,
                          prior_alpha_p_M = -0.5,
                          prior_alpha_p_SD = 1.0,
                          prior_kappa_mu_M = 1.5,
                          prior_kappa_mu_SD = 1.0,
                          prior_kappa_sigma_M = 0.0,
                          prior_kappa_sigma_SD = 0.3))

```

Visual Inspection and Comparison of Treatment Effects

The functions in this category enable visualization and statistical comparison of treatment effects based on posterior distributions from the fitted model. Both functions require the output from `cellmig()` as their primary input, from which they extract posterior samples of treatment effect parameters (δ_t). These functions compute pairwise differences between treatment groups and quantify the probability of differential effects. They return structured lists containing both numerical summaries (data frames with effect sizes, credible intervals, and probability values) and visualization objects (heatmaps or violin plots).

get_pairs() This function compares overall treatment effects between all pairs of treatment groups by computing differences in their posterior distributions.

The primary input is the fitted model object from `cellmig()`, specified via the `x` argument. For each pair of treatments (i, j), the function extracts posterior samples of δ_i and δ_j, computes the difference $\rho_{ij} = \delta_i - \delta_j$ (log-fold change), and calculates π_{ij} (probability of differential effect, where $\pi \approx 1$ indicates strong evidence for a difference). The function returns a list containing: (i) a `data.frame` with pairwise comparisons (including mean ρ, 95% HDI bounds, and π values); (ii) two heatmap visualizations showing ρ_{ij} and π_{ij} values, respectively; and (iii) a volcano plot visualization showing mean ρ_{ij} (95% HDIs as error bars) vs. π_{ij}. Setting `exponentiate = TRUE` converts log-fold changes (ρ_{ij}) to fold-changes ρ'_{ij} for easier interpretation.

```
# Compare all treatment pairs
pairs ← get_pairs(x = fit, exponentiate = FALSE)

# Access results and visualizations
head(pairs$summary)
pairs$plot_rho     # Heatmap of log-fold changes
pairs$plot_pi      # Heatmap of probabilities
pairs$plot_volcano # Heatmap of probabilities

```

get_violins() This function generates violin plots comparing posterior distributions of treatment effect differences for specific treatment group comparisons. The primary input is the fitted model object from `cellmig()` (`x` argument), along with user-specified target treatment group (`to_group`) and one or more comparison groups (`from_groups`). The function extracts posterior samples of treatment-level means, computes differences between specified groups (ρ), and produces a violin plot illustrating these posterior distributions. It returns a list containing: (i) a `data.frame` with ρ statistics and π values, and (ii) a `ggplot2` plot object. This function is useful for focused comparisons of specific treatments against a reference condition. Setting `exponentiate = TRUE` converts log-fold changes to fold-changes for easier interpretation.

```
# Generate violin plots for specific comparisons
violins ← get_violins(x = fit, from_groups = c("A", "B"),
                  to_group = "C", exponentiate = FALSE)

# Access results and plot
violins$ds    # Data frame with statistics
violins$plot  # ggplot2 violin plot

```
