# Supplementary figures and images for: Uncertainty-aware quantitative analysis of high-throughput live cell migration data
Source: PLoS Comput Biol. 2026 Jul 13;22(7):e1014472. doi: 10.1371/journal.pcbi.1014472 (PMC13387618; doi:10.1371/journal.pcbi.1014472)

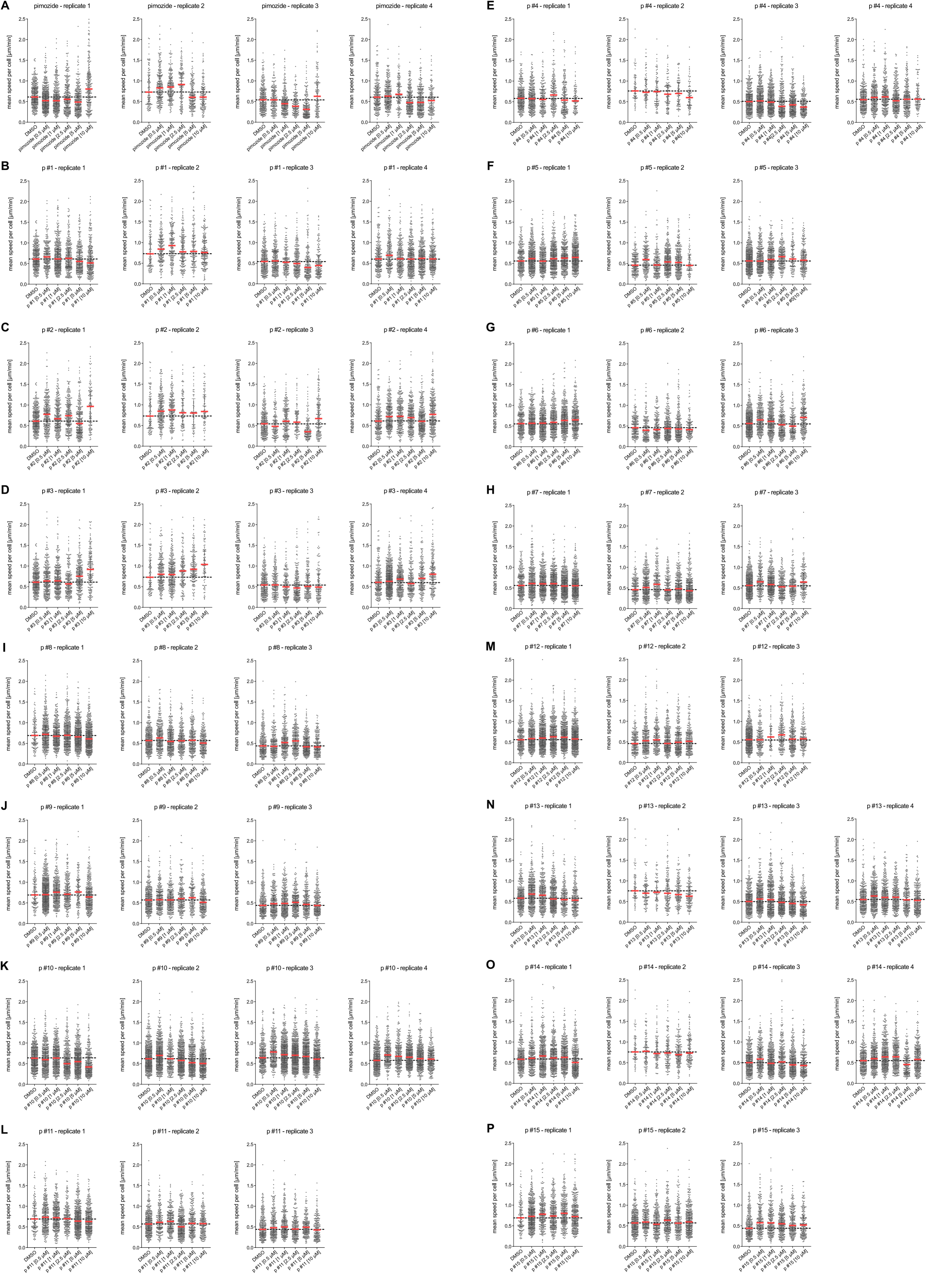

Supplement: S1 Fig — Single-cell migration velocity (y-axis; µm/min) across plates (panels) in Dataset 2 (application screen). In each panel, wells show compound and dose treatments, with individual cells depicted as dots within violin plots to visualize distribution density. Red horizontal lines indicate well-specific means, with error bars representing ± Standard Error of the Mean (SEM). The dashed black line corresponds to the plate-specific DMSO control mean, used as a baseline for comparison across treatments within plates. (PDF) [file pcbi.1014472.s006.pdf]

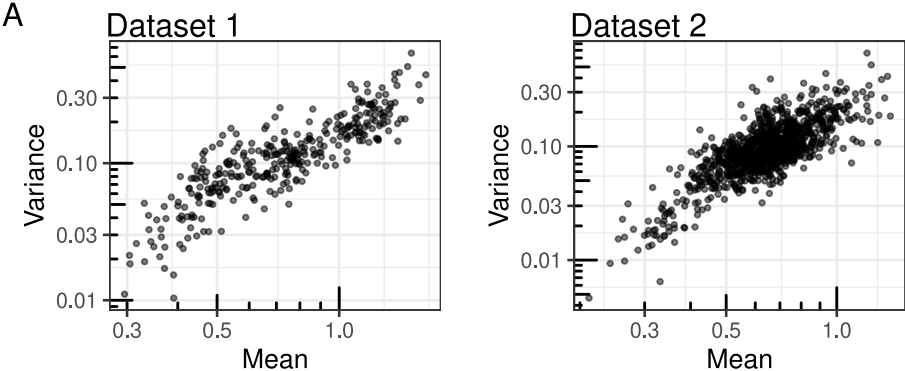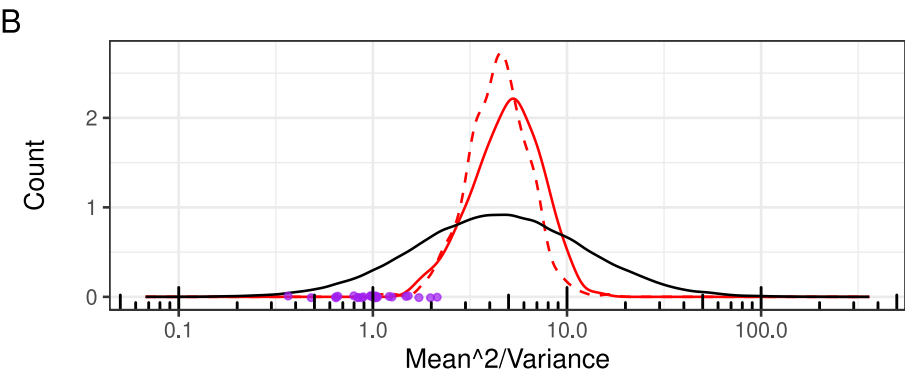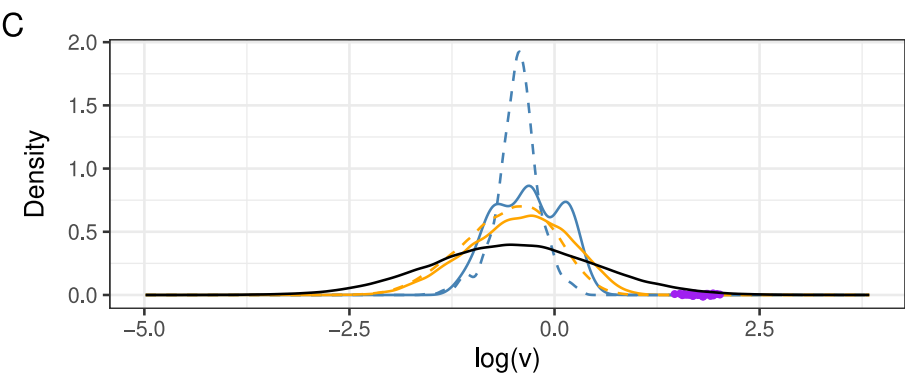

Supplement: S2 Fig — (A) Log–log relationship between variance (y-axis) and mean (x-axis) of well-specific cell velocities in Dataset 1 (left) and Dataset 2 (right). (B) Probability distribution of the empirically observed inverse squared coefficient of variation (κw=μw2/τw) for well-specific cell velocities (Dataset 1: solid red; Dataset 2: dashed red). The black line shows the prior distribution of μκ (μκ~Normal(μ=1.5,σ=1.0)), i.e., the mean of the population of log(κw) (log(κw)~Normal(μκ,σκ)). Purple dots indicate inverse squared coefficient of variation in 24 wells with control-treated neutrophils from [9]. The x-axis shows κw on a log10 scale. (C) Probability distributions of log-transformed cell velocities for individual cells (orange) and well-specific means (blue); solid lines indicate Dataset 1 and dashed lines indicate Dataset 2. The black line shows the normal prior distribution for αp (αp~Normal(μ=−0.5,σ=1.0)). Purple dots indicate mean velocities in 24 wells with control-treated neutrophils from [9]. Generated with ggplot2 [38] and patchwork [39]. (PDF) [file pcbi.1014472.s007.pdf]

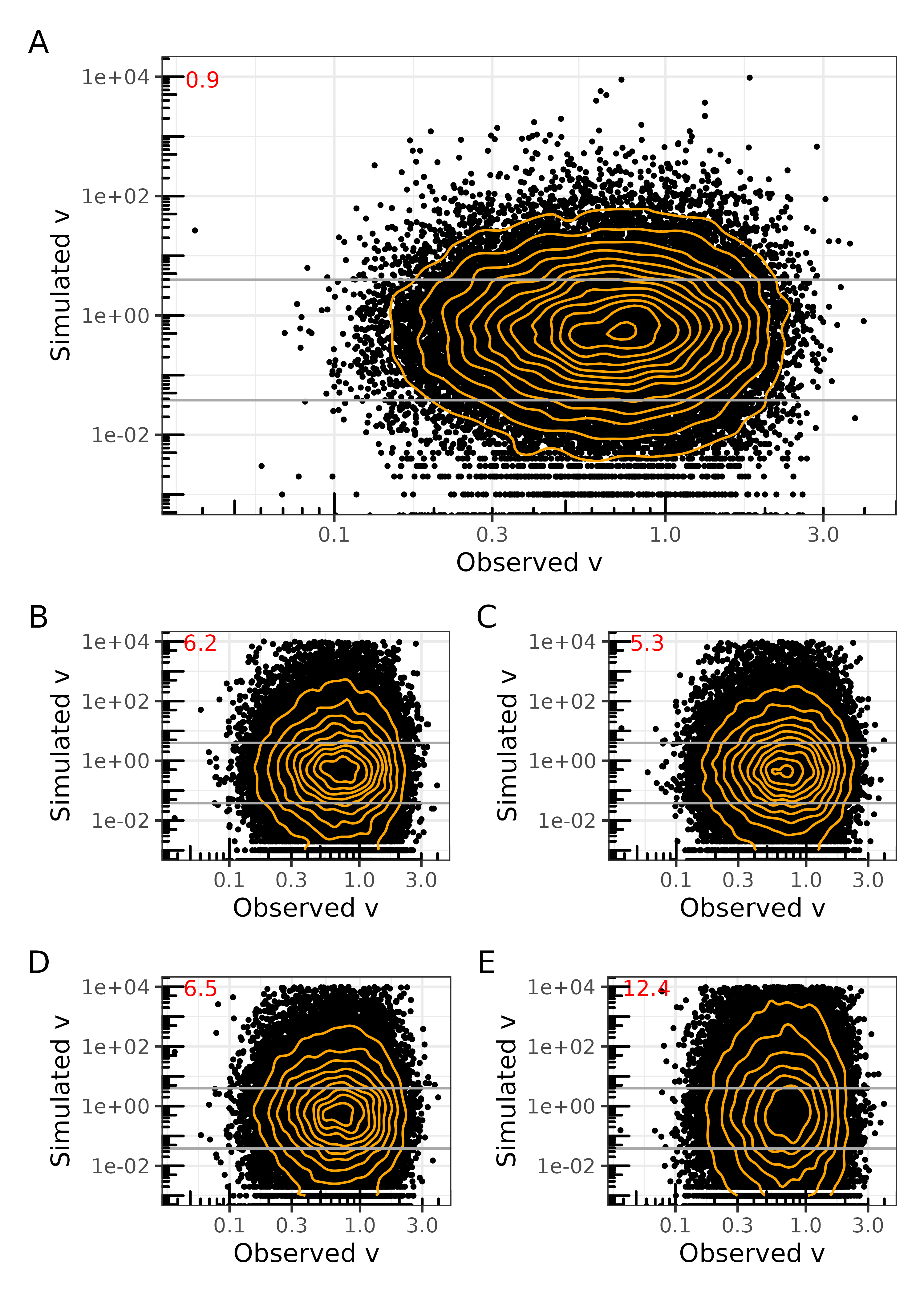

Supplement: S3 Fig — Simulated and observed cell velocities (v) for 29,503 cells are plotted on the y-axis (simulated) and x-axis (observed in Dataset 1), respectively, with both axes on a log10 scale. Orange contours represent the two-dimensional density of the data. Dark gray horizontal lines indicate the minimum and maximum of observed velocity in Dataset 1. Red labels in each panel shows the percentage of simulated velocities exceeding 100 µm/min (biologically implausible range). (A) Default prior configuration (SD = 1 prior on σbio,σtech,σδ~Normal+(0,1)). (B) Increased variability between technical replicates (SD = 3 prior on σtech~Normal+(0,3)). (C) Increased variability between biological replicates (SD = 3 prior on σbio~Normal+(0,3)). (D) Increased treatment effect variability (SD = 3 prior on σδ~Normal+(0,3)). (E) All three scale parameters get wider priors (SD = 3 prior on σbio,σtech,σδ~Normal+(0,3)). Panels B–E show that wider priors generate implausible velocities, supporting the default priors in (A). Generated with ggplot2 and patchwork. (PNG) [file pcbi.1014472.s008.png]

A

Dataset 1

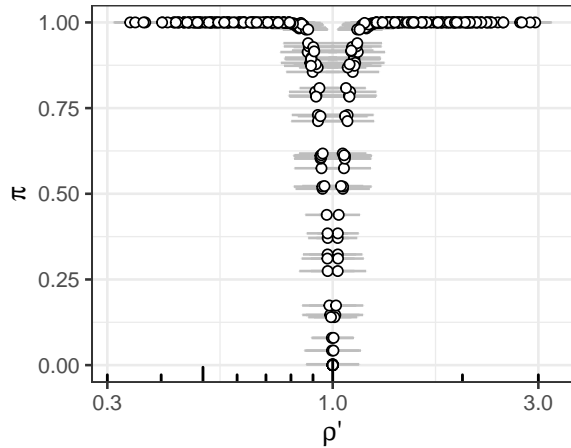

B

Dataset 2

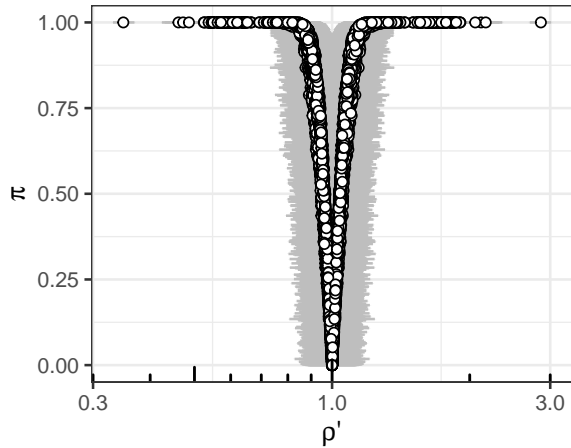

Supplement: S5 Fig — (A) Dataset 1: Differences in overall treatment effects (δt′) between treatment pairs. Dots represent the mean fold-change (ρ′, x-axis) with 95% HDI error bars. The y-axis represents the probability (π) of differential effect on migration. Top-priority hits are located in the upper corners: top-left (ρ′<1, π≥0.99) indicates strong migration suppressors; top-right (ρ′>1, π≥0.99) indicates strong migration enhancers. Dots near the center (ρ′≈1) or bottom (π≈0) represent negligible or uncertain effects. (B) Dataset 2: Same visualization as in (A). (PDF) [file pcbi.1014472.s010.pdf]

# Pimozide Derivatives

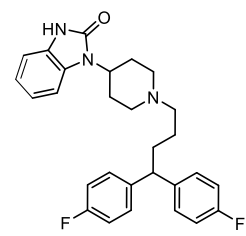

Pimozide

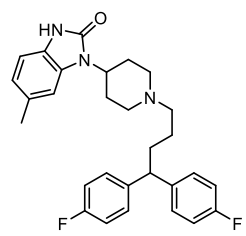

P #1

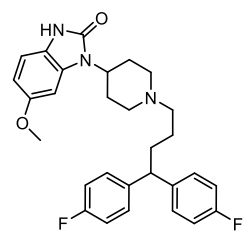

P #2

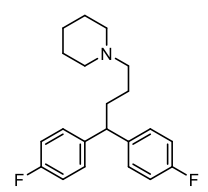

P #3

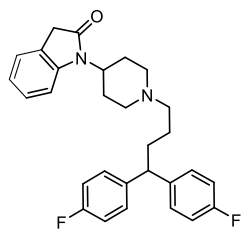

P #4

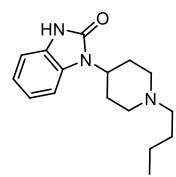

P #5

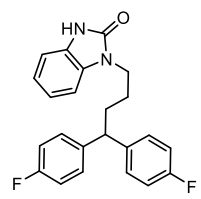

P #6

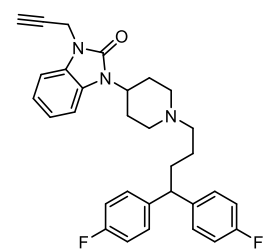

P #7

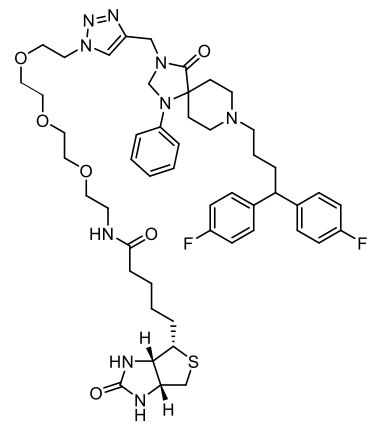

P #8

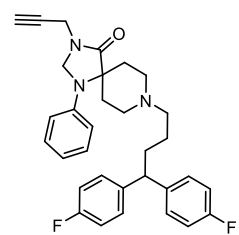

P #9

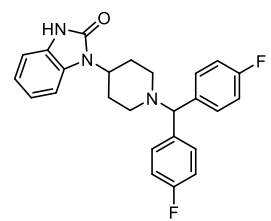

P #10

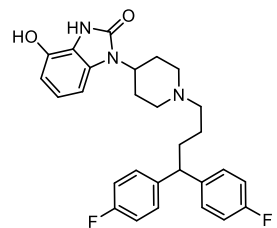

P #11

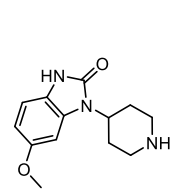

P #12

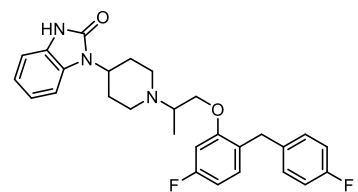

P #13

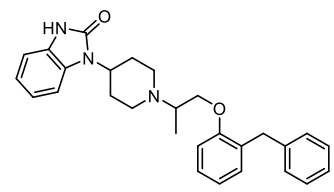

P #14

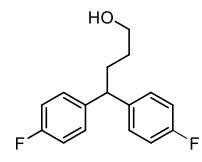

P #15

Supplement: S6 Fig — Corresponding chemical names are listed in S1 Table. The figure was created in ChemDraw Prime (Version 25.0.2.14). (PDF) [file pcbi.1014472.s011.pdf]

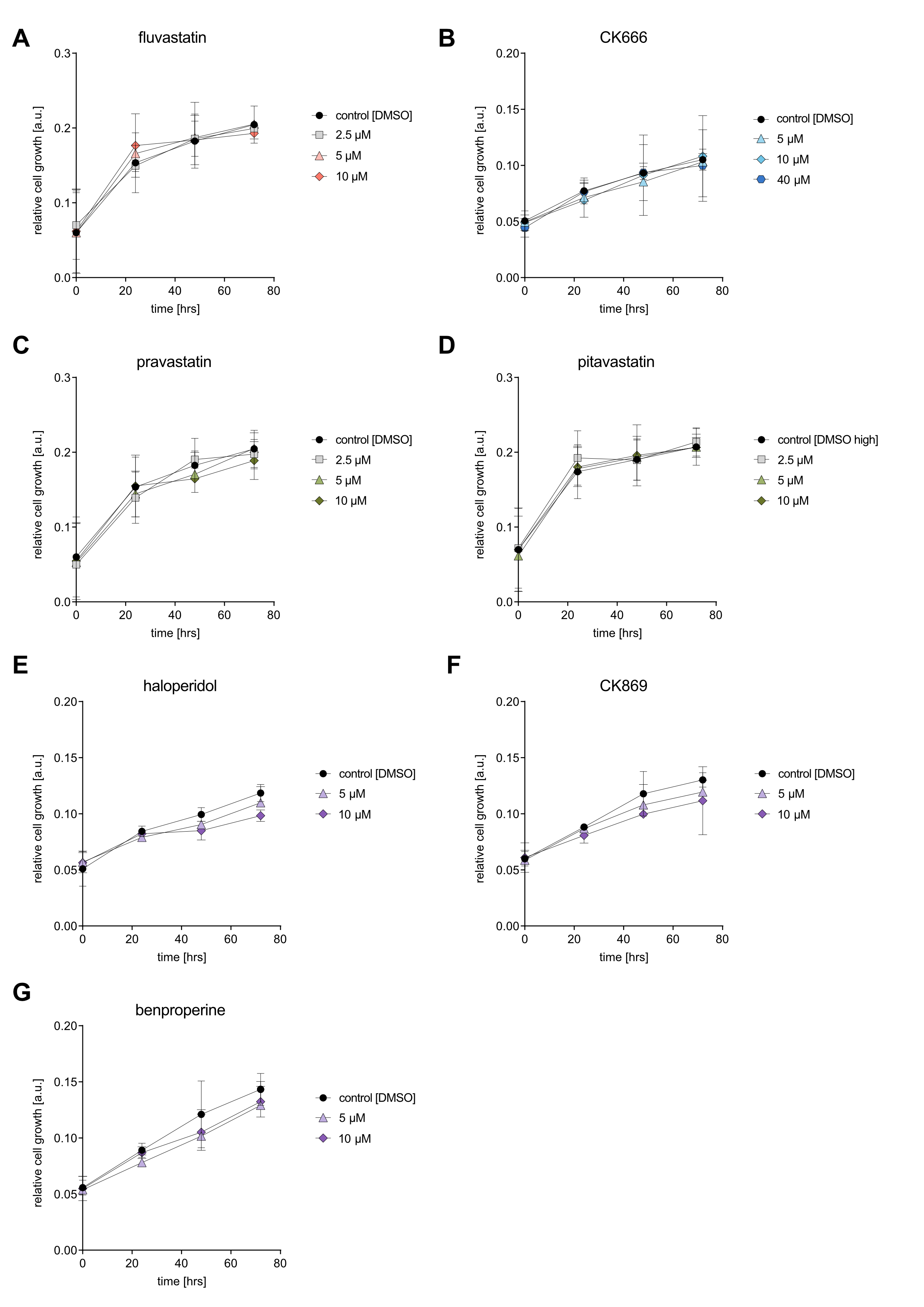

Supplement: S7 Fig — Cell viability assay for (A) fluvastatin (B) CK666 (C) pravastatin (D) pitavastatin (E) haloperidol (F) CK869, and (G) benproperine in ASPC1 cells, tested at indicated concentrations and timepoints. DMSO at the concentration present in the highest compound concentration was used as vehicle only control. Symbols represent mean values, with error bars indicating ± SEM calculated from three independent experiments. (PNG) [file pcbi.1014472.s012.png]

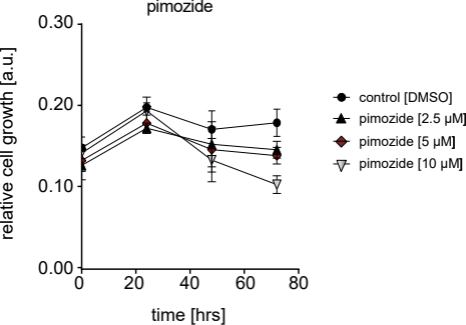

Supplement: S8 Fig — Cell viability assay for pimozide in ASPC1 cells, tested at indicated concentrations and timepoints. Symbols represent mean values, with error bars indicating ± SEM calculated from three independent experiments. (PDF) [file pcbi.1014472.s013.pdf]

**A**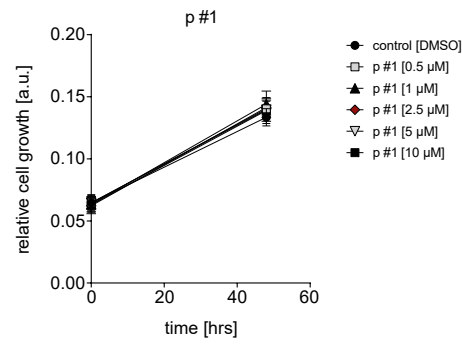**B**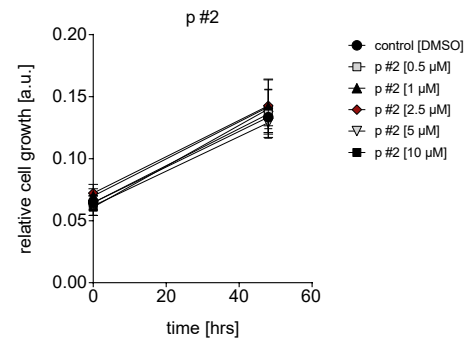**C**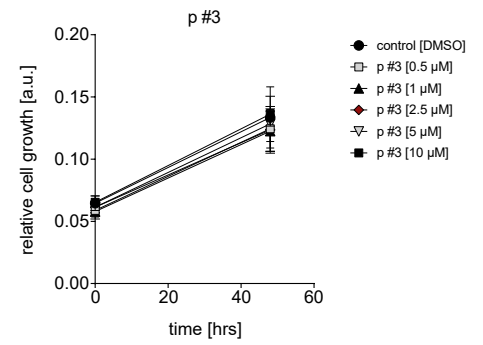**D**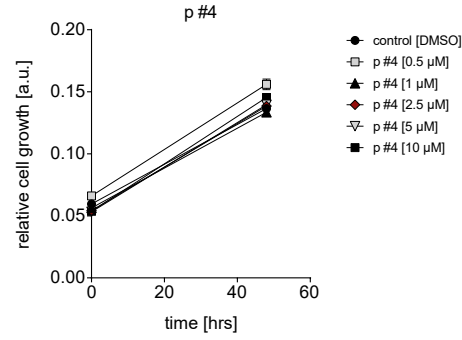**E**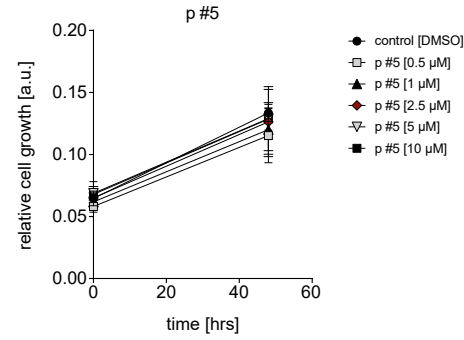**F**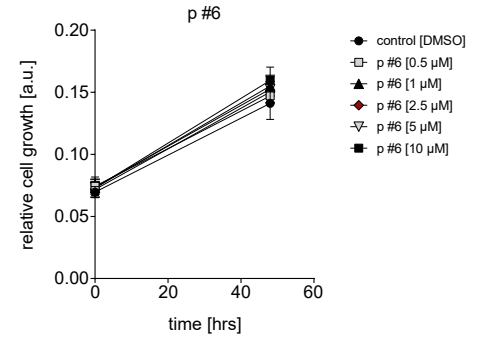**G**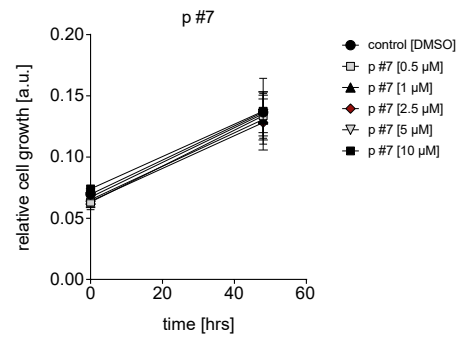**H**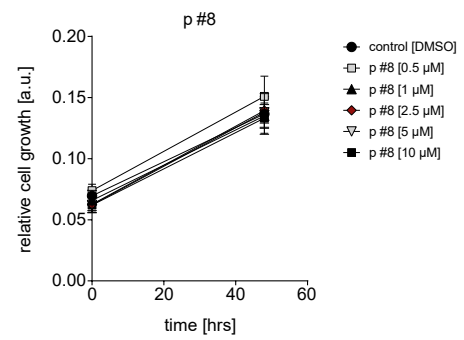**I**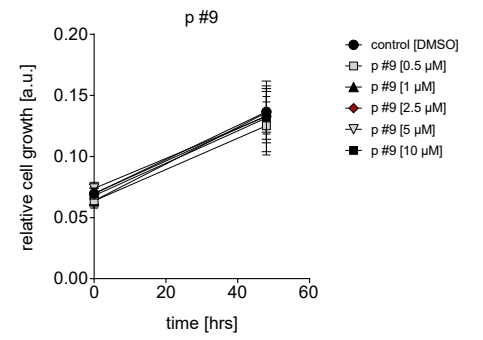**J**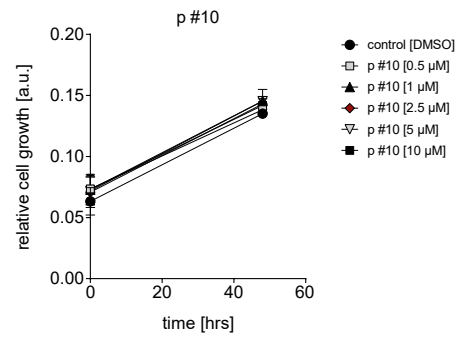**K**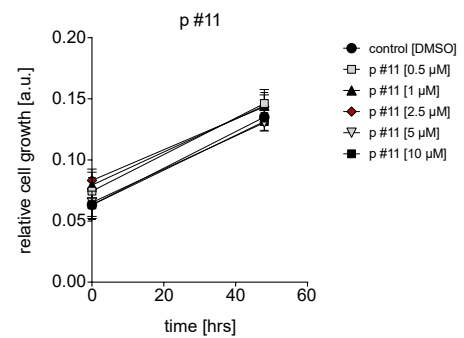**L**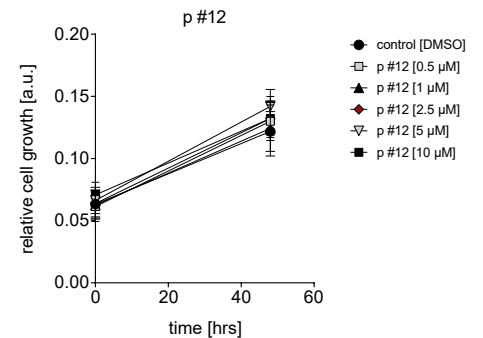**M**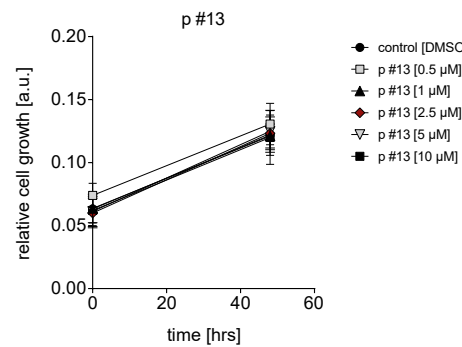**N**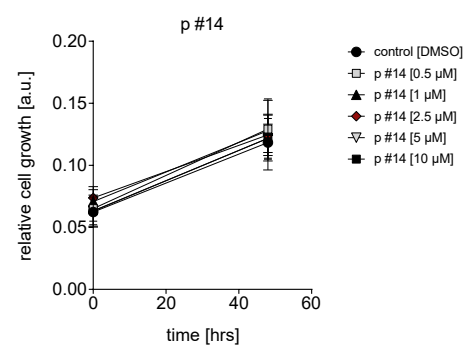**O**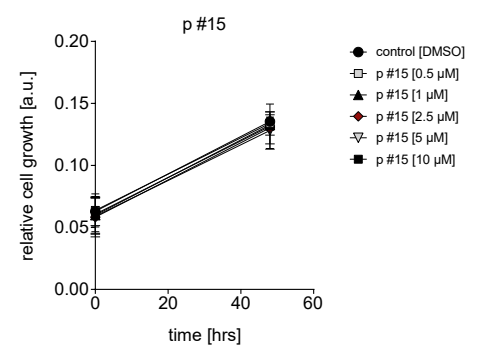

Supplement: S9 Fig — Cell viability assay for 15 pimozide derivatives (panels) in ASPC1 cells, tested at indicated concentrations and timepoints. Symbols represent mean values, with error bars indicating ± SEM calculated from three independent experiments. Chemical names are provided in S1 Table. (PDF) [file pcbi.1014472.s014.pdf]

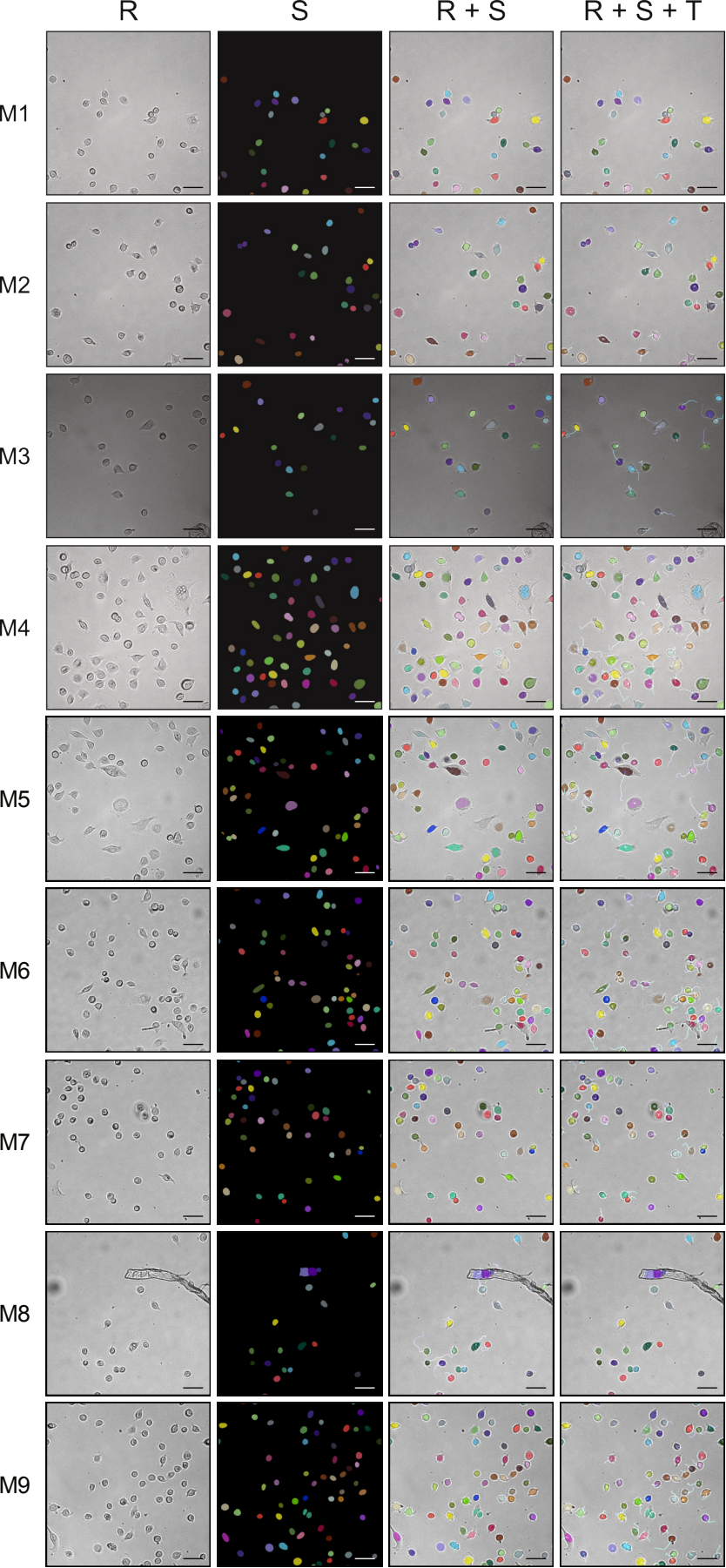

Supplement: S10 Fig — Representative outputs from the cell segmentation and tracking pipeline for nine movies (M1-M9). For each indicated field/condition, images are displayed in four columns: raw (R), segmentation (S), raw and segmentation (R + S), and raw, segmentation and tracking (R + S + T) views. The R column shows the original bright-field microscopy image used for analysis. The S column shows the identified cell objects after image segmentation. The R + S column shows the segmented cells overlaid with raw image. The R + S + T column overlays segmentation and tracking results onto the original image, providing a visual summary of cell identification and trajectories. Scale bar = 50 µm. Images were visualized with Napari. Affinity Designer was used for final editing and layout design. (PDF) [file pcbi.1014472.s015.pdf]

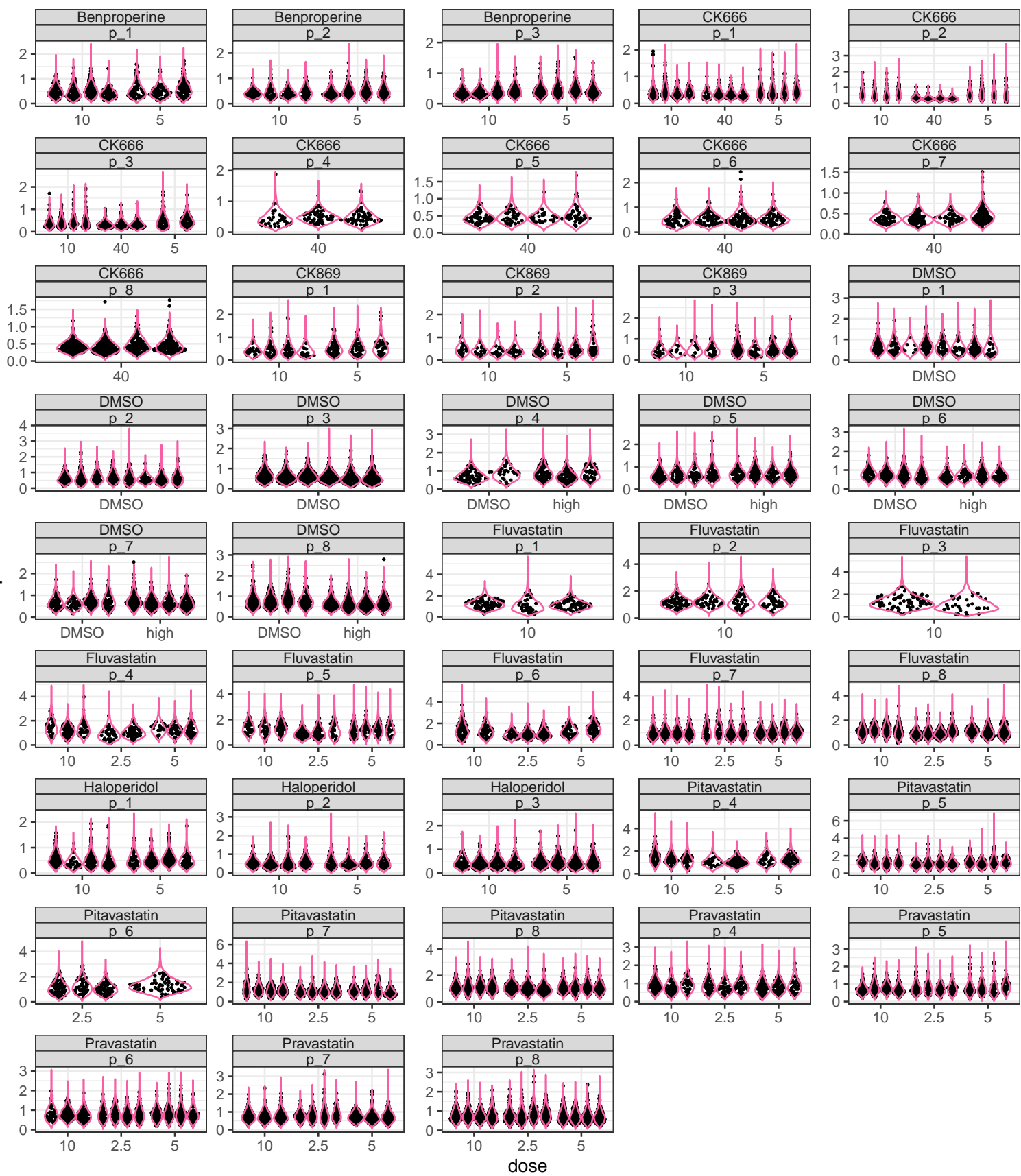

Supplement: S11 Fig — Each panel represents a specific chemical compound applied to wells on individual plates. Panel labels indicate the compound name and the corresponding plate identifier. Within each panel, black violins represent the distribution of observed cell velocities (y-axis), with individual cells shown as black dots. Pink violins show the model’s predicted distribution of cell velocities for the same wells. The x-axis denotes the treatment dose applied to the cells in each well. The close overlap between black and pink violins indicates strong agreement between observed and predicted velocities, suggesting that the model accurately retrodicts the observed data. Generated with ggplot2. (PDF) [file pcbi.1014472.s016.pdf]

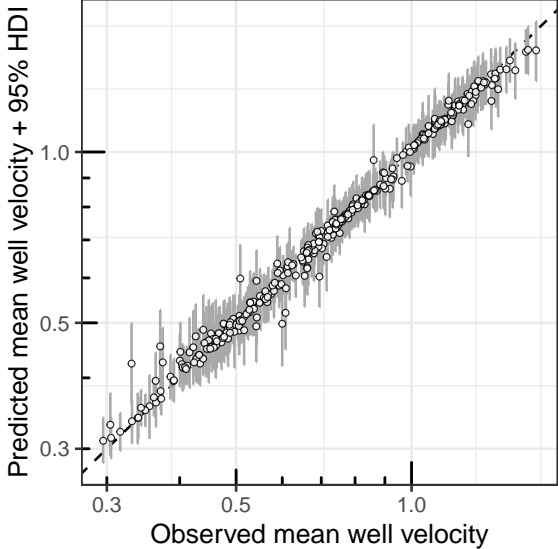

Supplement: S12 Fig — Each dot represents a well, with its observed and predicted mean velocity plotted on the x- and y-axes, respectively. Gray vertical error bars indicate the 95% highest density intervals (HDIs) of the predicted mean velocities. A black dotted diagonal line marks the identity line (x = y); dots lying close to this line indicate good agreement between observed and predicted values, suggesting that the model can retrodict the observed data well. Generated with ggplot2. (PDF) [file pcbi.1014472.s017.pdf]

A

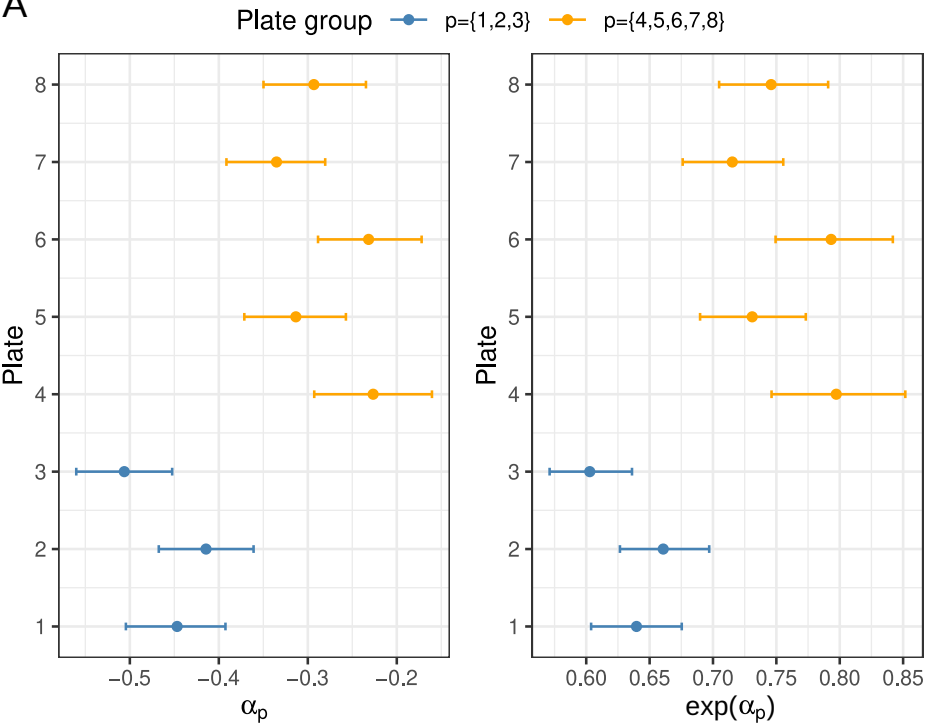

B

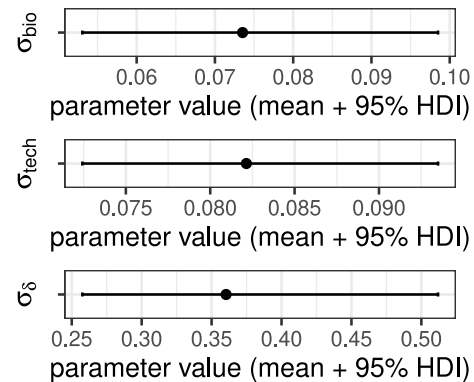

C

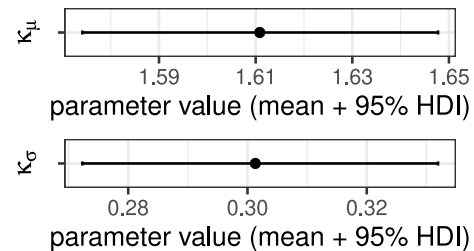

Supplement: S13 Fig — (A) αp (left panel) defined as the mean migration velocity (on log-scale) of the control treatment DMSO on each plate, and the exponentiated version (right panel), defined as the mean migration velocity (µm/min). Blue and orange dots and error bars correspond to plates from two different plate groups; (B) σbio defined as the standard deviation of treatment effects between biological replicates (plates), σtech defined as the standard deviation of treatment effects between technical replicates (wells on plate), and σδ defined as the standard deviation of the population of overall treatment effects. (C) mean (μκ) and standard deviation (σκ) of the normal distribution of well-specific log(κw) parameters. (PDF) [file pcbi.1014472.s018.pdf]

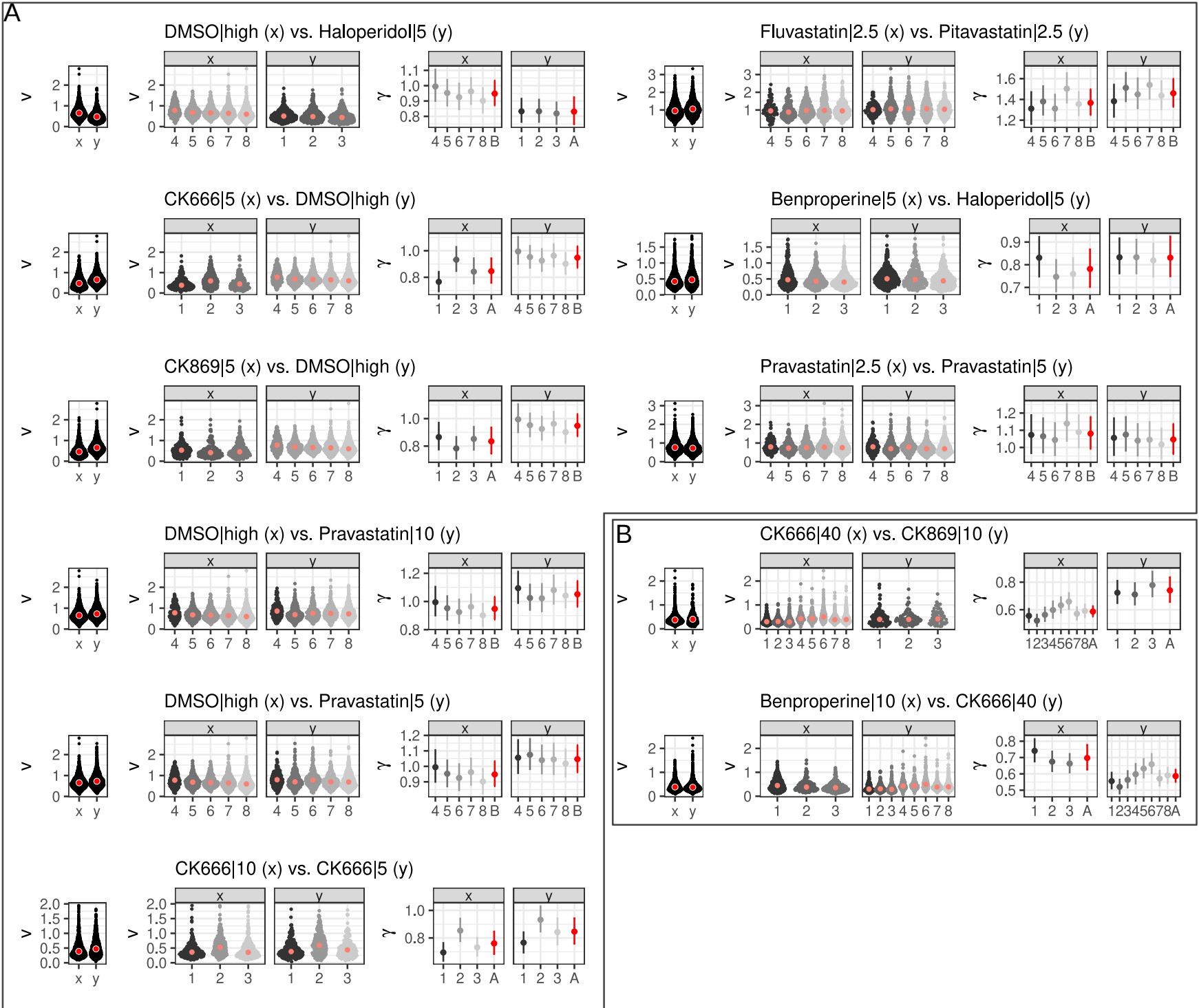

Supplement: S15 Fig — (A) Nine treatment pairs where the H-test indicates significance (adjusted p-value < 0.01) but cellmig indicates low probability (π<0.99) of differential effect velocity, likely due to unmodeled variability. (B) Two pairs where the H-test indicates non-significance but cellmig indicates strong evidence, likely due to batch correction. Dots are cell velocities in each treatment group (x and y). Cell velocities are shown as pooled across plates from experiment A and B (left panels), or within individual plates (middle panels). Mean plate-specific treatment effects and overall treatment effects and their 95% HDIs are shown as gray/red dots and error bars (right panels). Larger dots are group medians. (PDF) [file pcbi.1014472.s020.pdf]

From treatment

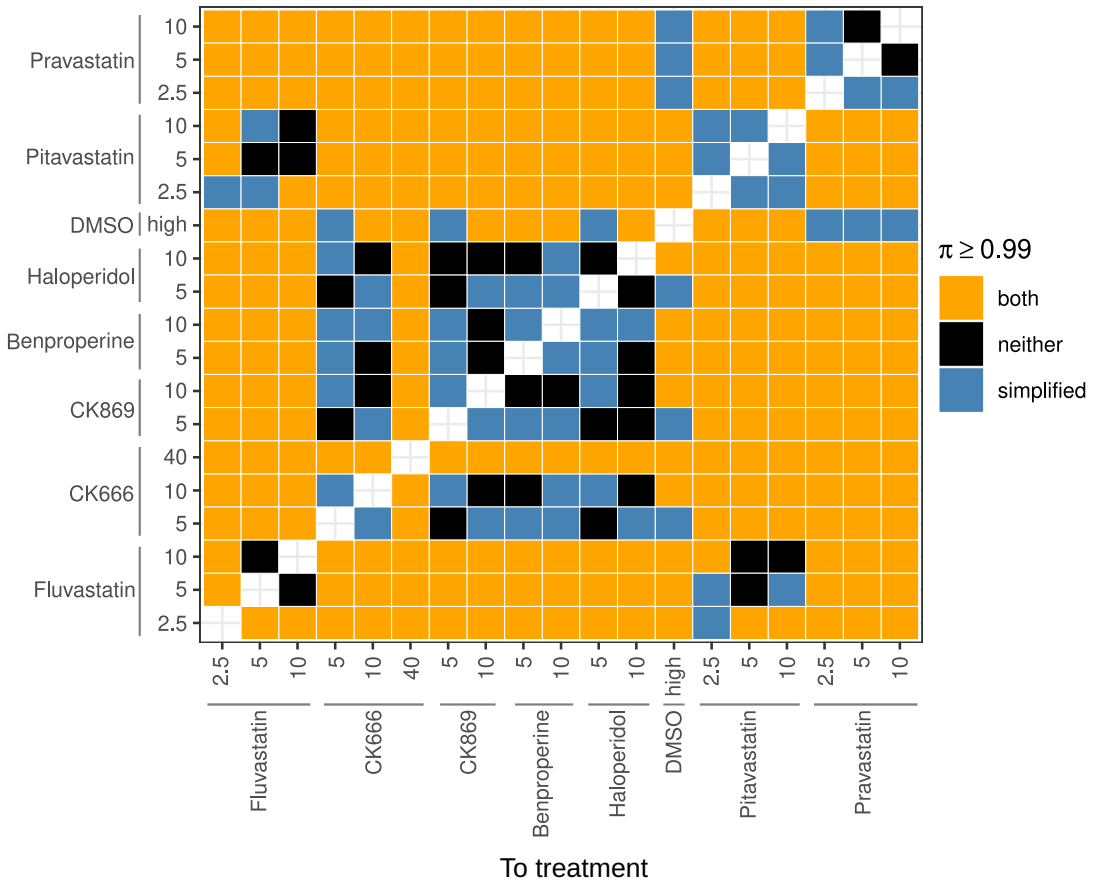

Supplement: S16 Fig — Tiles represent pairwise treatment comparisons colored by agreement on the probability of differential effect (πij). Orange: both methods detect a difference (π≥0.99). Black: neither method detects a difference (π<0.99). Blue: simplified model detects a difference (π≥0.99) but cellmig does not. Diagonal entries are omitted; the matrix is symmetric. (PDF) [file pcbi.1014472.s021.pdf]

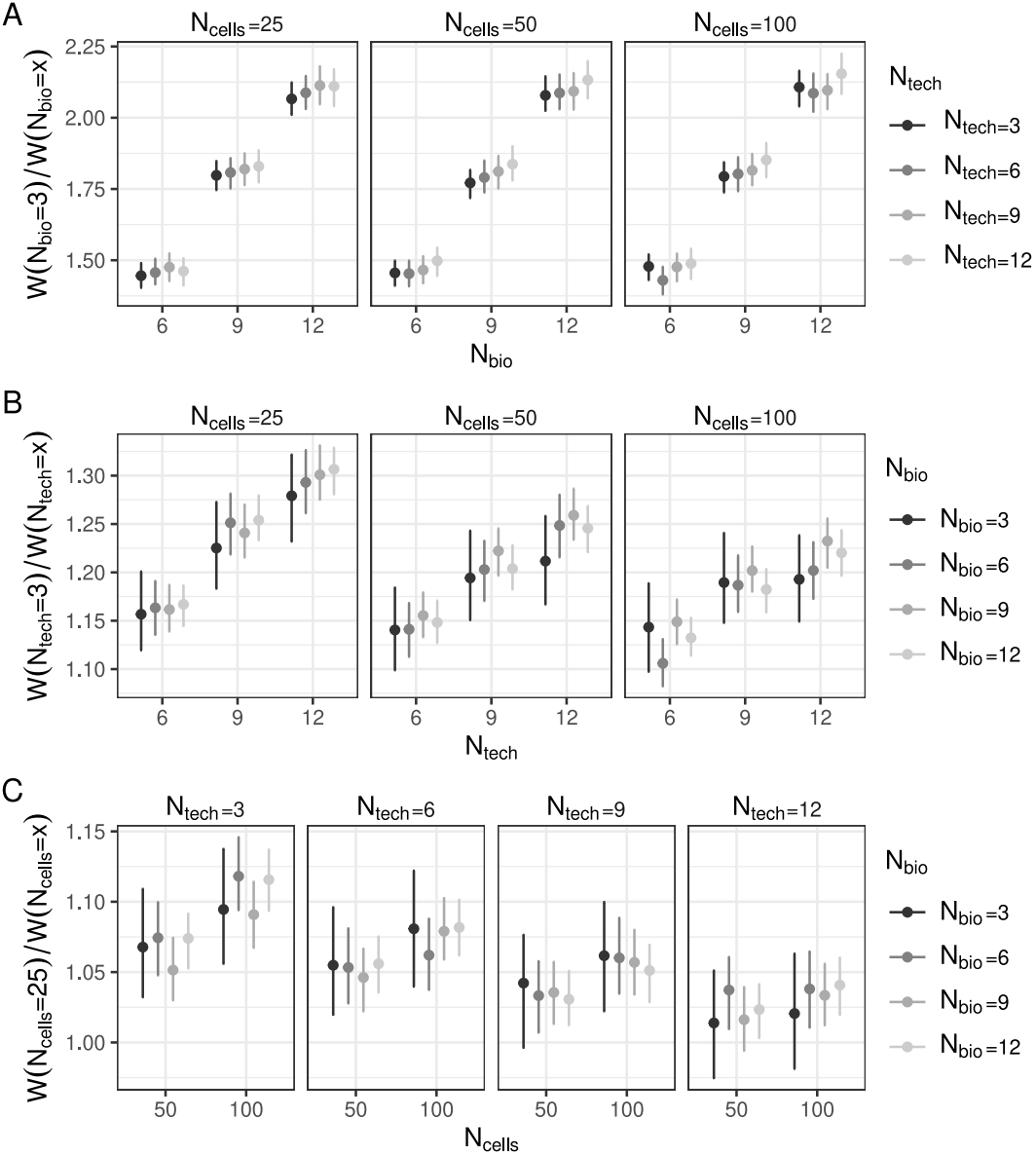

Supplement: S17 Fig — (A) Fold change in W between Nbio = 3 vs. Nbio = 6, 9, and 12 for specific combinations of Ntech and Ncells. (B) Fold change in W between Ntech = 3 vs. Ntech = 6, 9, and 12 for specific combinations of Nbio and Ncells. (C) Fold change in W between Ncells = 25 vs. Ncells = 50 and 100 for specific combinations of Nbio and Ntech. Dots are mean fold changes of 1,000 bootstraps, error bars are 95% HDIs of bootstrapped fold changes. (PDF) [file pcbi.1014472.s022.pdf]

A

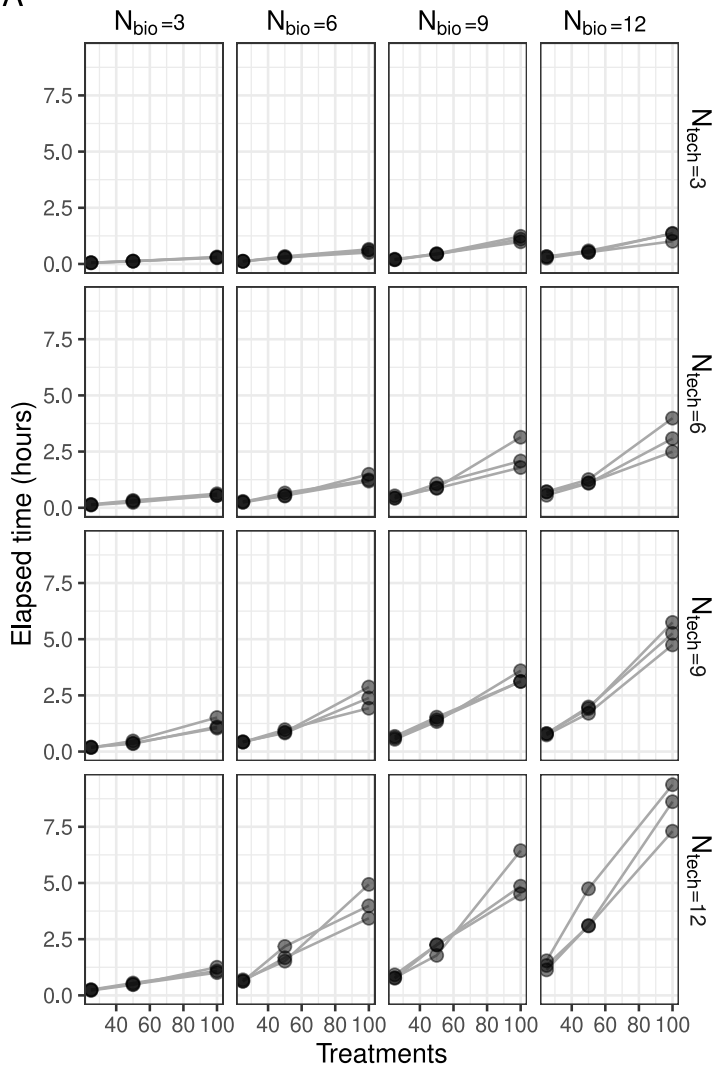

B

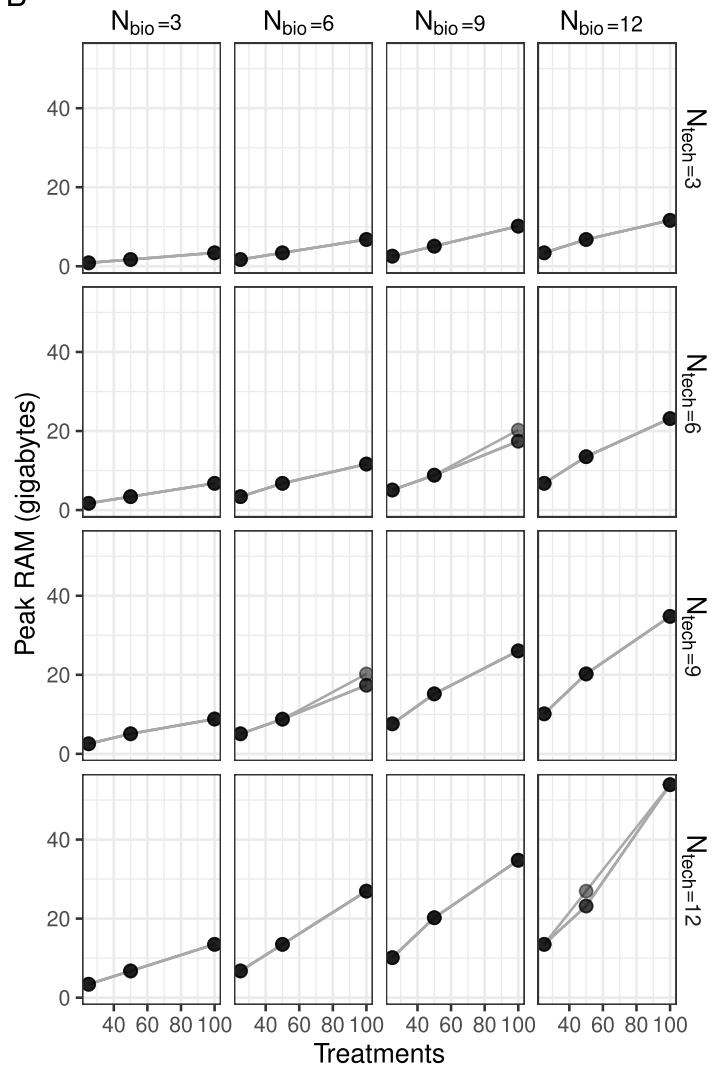

Supplement: S18 Fig — (A) Elapsed time (hours) and (B) peak random access memory (RAM) usage (gigabytes) for cellmig model fitting across varying experimental configurations. Each panel corresponds to a specific combination of biological replicates (Nbio, rows) and technical replicates (Ntech, columns). The number of treatment groups (Ngroup) was varied from 25 to 100. Gray lines connect results from the same synthetic dataset (n = 3 per configuration), while points represent individual model fits. Benchmarks reflect a single Markov chain run on an AMD EPYC 75F3 workstation with Ncells = 80 cells per well. (PDF) [file pcbi.1014472.s023.pdf]

A

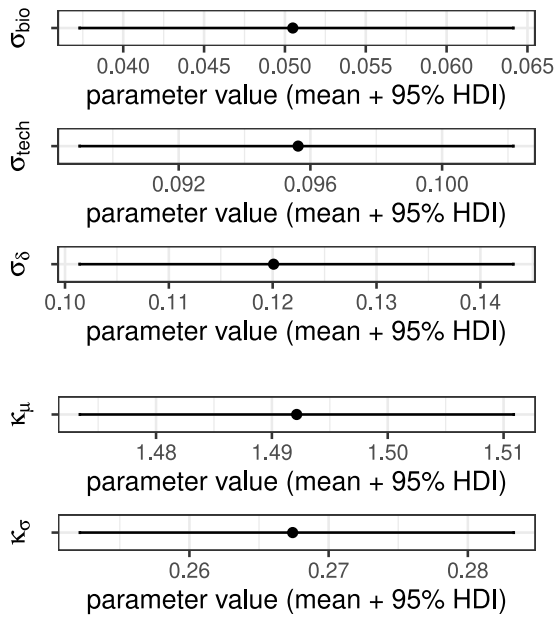

B

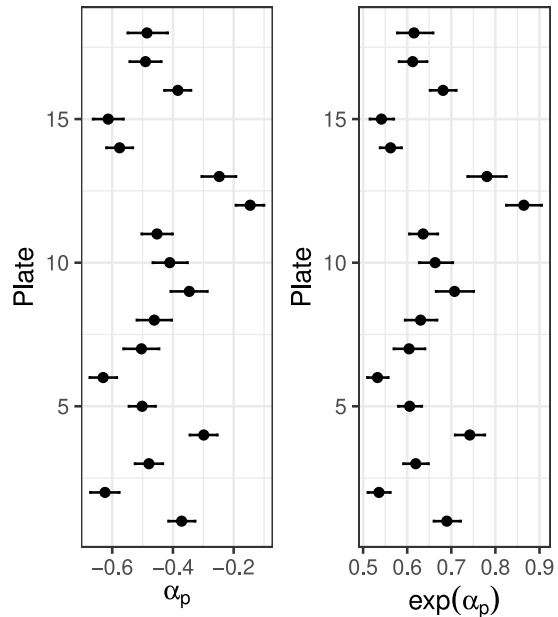

C

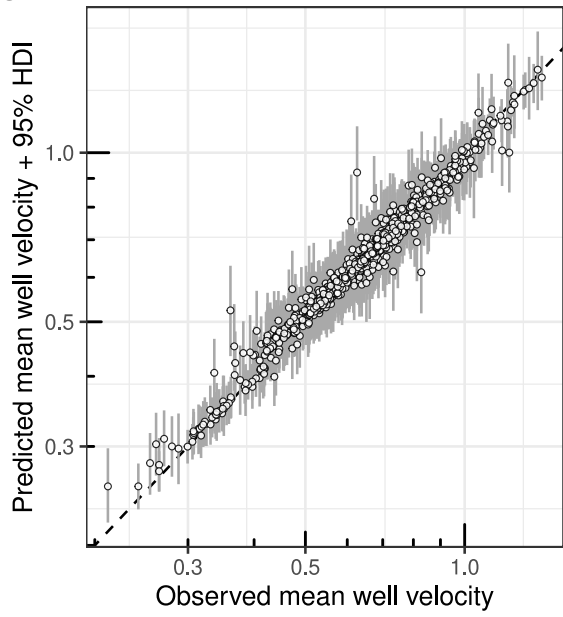

Supplement: S19 Fig — (A) Means and 95% HDIs of σbio defined as the standard deviation of treatment effects between biological replicates (plates), σtech defined as the standard deviation of treatment effects between technical replicates (wells on plate), and σδ defined as the standard deviation of the population of overall treatment effects (δt). Also shown are the mean (μκ) and standard deviation (σκ) of the normal distribution of well-specific log(κw) parameters. (B) Means and 95% HDIs of αp (left panel) defined as the mean migration velocity (on log-scale) of the control treatment DMSO on each plate, and the exponentiated version (right panel), defined as the mean migration velocity (µm/min). (C) Posterior predictive check of mean well velocities in Dataset 2. Each dot represents a well, with its observed and predicted mean velocities plotted on the x- and y-axes, respectively. Gray vertical error bars indicate the 95% highest density intervals (HDIs) of the predicted mean velocities. A black dotted diagonal line marks the identity line (x = y); dots lying close to this line indicate good agreement between observed and predicted values, suggesting that the model can retrodict the observed data well. Generated with ggplot2 and patchwork. (PDF) [file pcbi.1014472.s024.pdf]

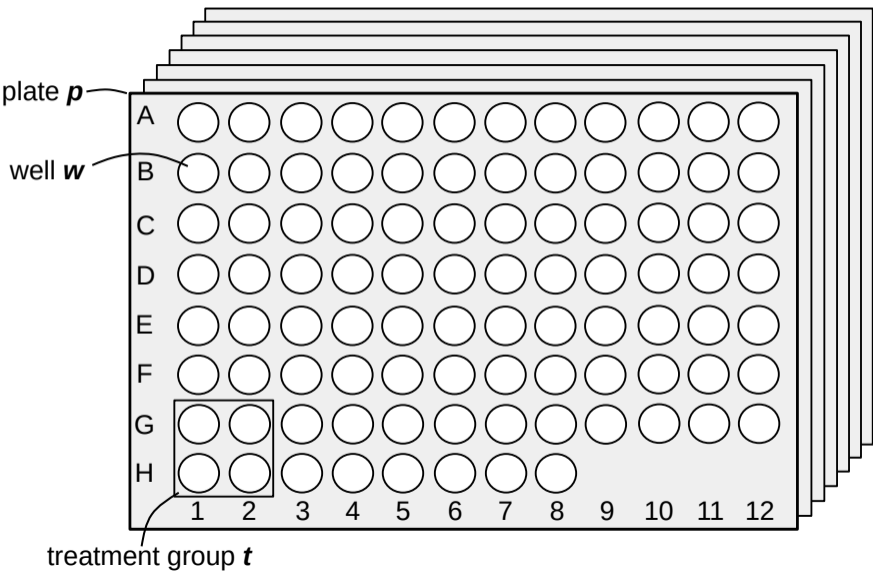

Supplement: S20 Fig — A number of cells are seeded in well w, position on 96-well plate p. At least 4 wells on a plate are treated with treatment group t (chemical compound administered at a specific concentration). (PDF) [file pcbi.1014472.s025.pdf]
