# Supplementary material for: Uncertainty-aware quantitative analysis of high-throughput live cell migration data
Source: PLoS Comput Biol. 2026 Jul 13;22(7):e1014472. doi: 10.1371/journal.pcbi.1014472 (PMC13387618; doi:10.1371/journal.pcbi.1014472)
Supplement: S4 Fig — (A) Hierarchical cellmig model. Scatter plots faceted by parameter comparing posterior means (points) and 95% Highest Density Intervals (error bars) between default priors (σbio,σtech,σδ~Normal+(0,1), x-axis) and wide priors (σbio,σtech,σδ~Normal+(0,3), y-axis), with the dashed diagonal line representing identity. Parameters include treatment effects (δt, δtp), plate intercepts (αp), well-specific means (μ) and shape parameters (κ), and variance components. (B) Simplified Bayesian model. Comparison of posterior means and 95% HDIs between default prior (σδ~Normal+(0,1), x-axis) and wide prior (σδ~Normal+(0,3), y-axis) priors for treatment effects (δt), intercept (α), means (μ), shape parameters (κ), and variance (σδ). Close agreement indicates posterior inferences are robust to prior specification. (PDF) [file pcbi.1014472.s009.pdf]

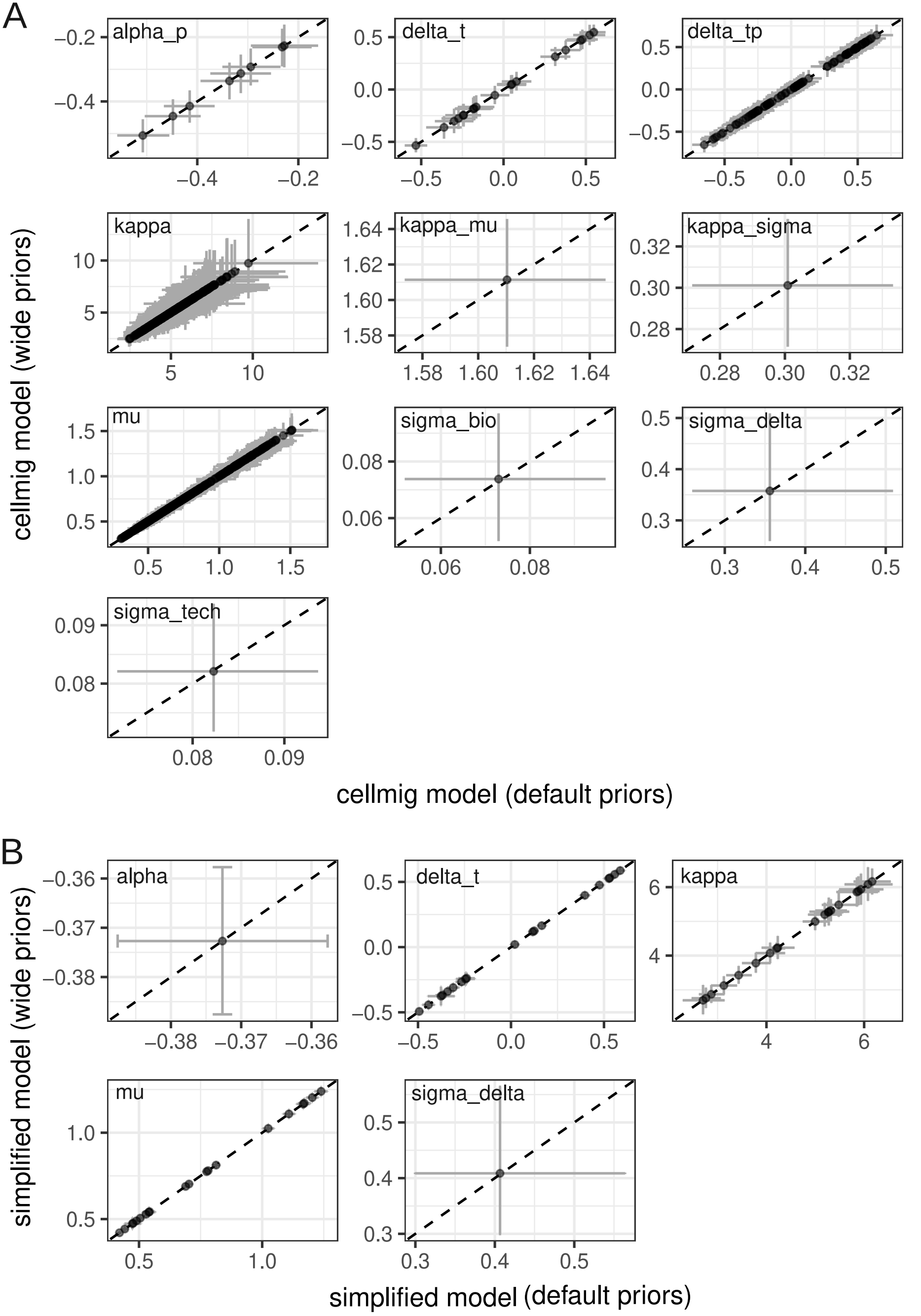

A
alpha_p
delta_t
delta_tp
kappa
kappa_mu
kappa_sigma
mu
sigma_bio
sigma_delta
sigma_tech
cellmig model (wide priors)
cellmig model (default priors)
B
alpha
delta_t
kappa
mu
sigma_delta
simplified model (wide priors)
simplified model (default priors)
