# Supplementary material for: Uncertainty-aware quantitative analysis of high-throughput live cell migration data
Source: PLoS Comput Biol. 2026 Jul 13;22(7):e1014472. doi: 10.1371/journal.pcbi.1014472 (PMC13387618; doi:10.1371/journal.pcbi.1014472)
Supplement: S14 Fig — Black dots represent comparisons based on cell velocity data from individual plates: y-axis shows the −log10 p-values obtained from a post hoc analysis using the Dunn’s test Benjamini-Hochberg FDR correction; x-axis shows the pairs of compared treatment groups. Green crosses represent the same statistical comparisons based on pooled cell velocities across plates. The horizontal dashed line represents p = 0.05. (PDF) [file pcbi.1014472.s019.pdf]

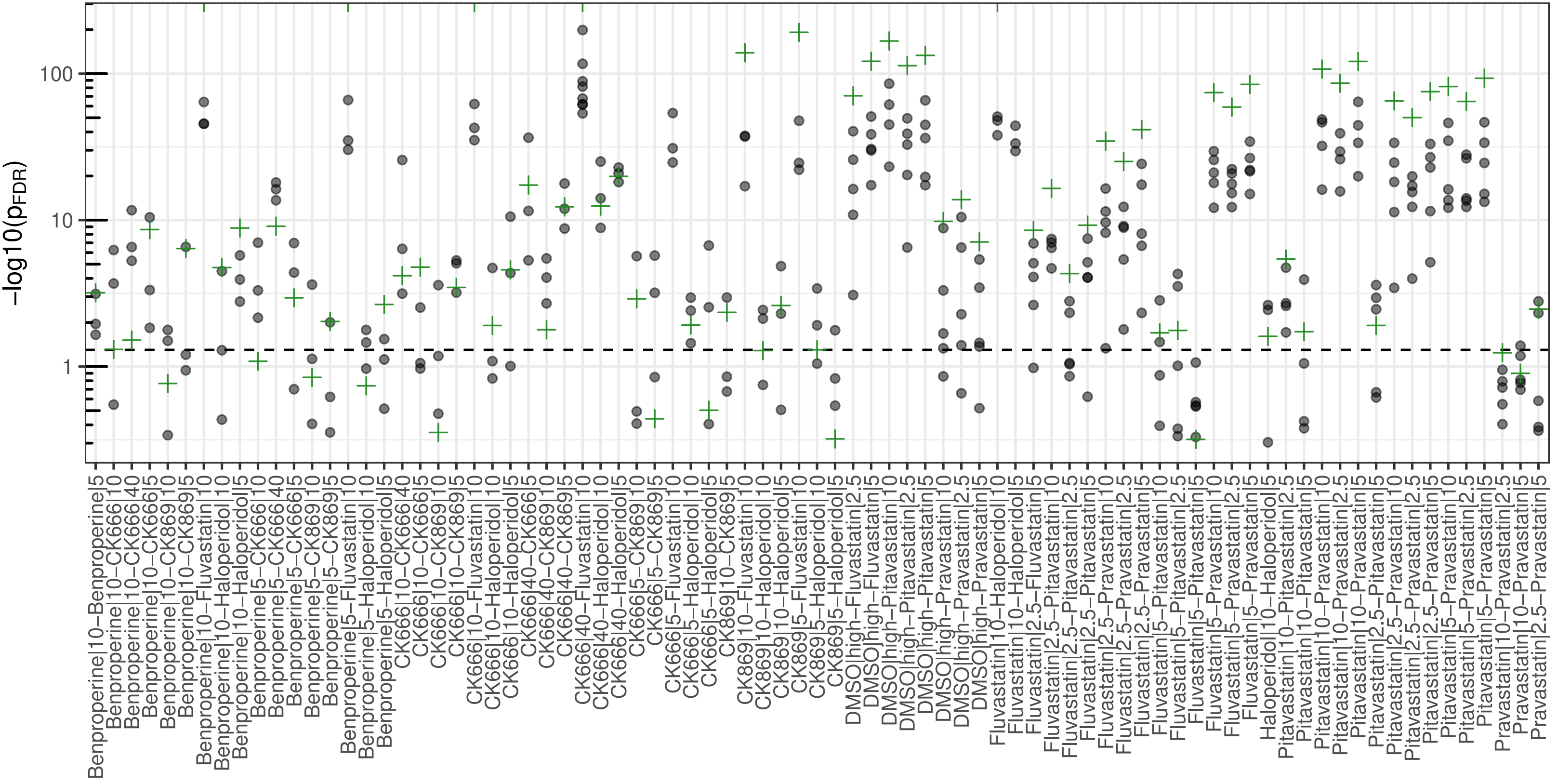
−log10(pFDR)
100
10
1
Benproperine|10–Benproperine|5
Benproperine|10–CK666|10
Benproperine|10–CK666|40
Benproperine|10–CK666|5
Benproperine|10–CK869|10
Benproperine|10–CK869|5
Benproperine|10–Fluvastatin|10
Benproperine|10–Haloperidol|10
Benproperine|10–Haloperidol|5
Benproperine|5–CK666|10
Benproperine|5–CK666|40
Benproperine|5–CK666|5
Benproperine|5–CK869|10
Benproperine|5–CK869|5
Benproperine|5–Fluvastatin|10
Benproperine|5–Haloperidol|10
Benproperine|5–Haloperidol|5
CK666|10–CK666|40
CK666|10–CK666|5
CK666|10–CK869|10
CK666|10–CK869|5
CK666|10–Fluvastatin|10
CK666|10–Haloperidol|10
CK666|10–Haloperidol|5
CK666|40–CK666|5
CK666|40–CK869|10
CK666|40–CK869|5
CK666|40–Fluvastatin|10
CK666|40–Haloperidol|10
CK666|40–Haloperidol|5
CK666|5–CK869|10
CK666|5–CK869|5
CK666|5–Fluvastatin|10
CK666|5–Haloperidol|10
CK666|5–Haloperidol|5
CK869|10–CK869|5
CK869|10–Fluvastatin|10
CK869|10–Haloperidol|10
CK869|10–Haloperidol|5
CK869|5–Fluvastatin|10
CK869|5–Haloperidol|10
CK869|5–Haloperidol|5
DMSO|high–Fluvastatin|2.5
DMSO|high–Fluvastatin|5
DMSO|high–Pitavastatin|10
DMSO|high–Pitavastatin|2.5
DMSO|high–Pitavastatin|5
DMSO|high–Pravastatin|10
DMSO|high–Pravastatin|2.5
DMSO|high–Pravastatin|5
Fluvastatin|10–Haloperidol|10
Fluvastatin|10–Haloperidol|5
Fluvastatin|2.5–Fluvastatin|5
Fluvastatin|2.5–Pitavastatin|10
Fluvastatin|2.5–Pitavastatin|2.5
Fluvastatin|2.5–Pitavastatin|5
Fluvastatin|2.5–Pravastatin|10
Fluvastatin|2.5–Pravastatin|2.5
Fluvastatin|2.5–Pravastatin|5
Fluvastatin|5–Pitavastatin|10
Fluvastatin|5–Pitavastatin|2.5
Fluvastatin|5–Pitavastatin|5
Fluvastatin|5–Pravastatin|10
Fluvastatin|5–Pravastatin|2.5
Fluvastatin|5–Pravastatin|5
Haloperidol|10–Haloperidol|5
Pitavastatin|10–Pitavastatin|2.5
Pitavastatin|10–Pitavastatin|5
Pitavastatin|10–Pravastatin|10
Pitavastatin|10–Pravastatin|2.5
Pitavastatin|10–Pravastatin|5
Pitavastatin|2.5–Pitavastatin|5
Pitavastatin|2.5–Pravastatin|10
Pitavastatin|2.5–Pravastatin|2.5
Pitavastatin|2.5–Pravastatin|5
Pitavastatin|5–Pravastatin|10
Pitavastatin|5–Pravastatin|2.5
Pitavastatin|5–Pravastatin|5
Pravastatin|10–Pravastatin|2.5
Pravastatin|10–Pravastatin|5
Pravastatin|2.5–Pravastatin|5
