# Supplementary material for: Uncertainty-aware quantitative analysis of high-throughput live cell migration data
Source: PLoS Comput Biol. 2026 Jul 13;22(7):e1014472. doi: 10.1371/journal.pcbi.1014472 (PMC13387618; doi:10.1371/journal.pcbi.1014472)
Supplement: S1 Table — Identifiers (IDs) and names of chemical compounds. (PDF) [file pcbi.1014472.s026.pdf]

# Supplementary information

## S1 Table: Chemical compound overview

| ID | Chemical name |
|---|---|
| Pimozide | 3-[1-[4,4-Bis(4-fluorophenyl)butyl]piperidin-4-yl]-1H-benzo[d]imidazol-2(3H)-one |
| P #1 | 3-[1-[4,4-Bis(4-fluorophenyl)butyl]piperidin-4-yl]-5-methyl-1H-benzo[d]imidazol-2(3H)-one |
| P #2 | 3-[1-[4,4-Bis(4-fluorophenyl)butyl]piperidin-4-yl]-5-methoxy-1H-benzo[d]imidazol-2(3H)-one |
| P #3 | 4,4-Bis(4-fluorophenyl)butylpiperidine |
| P #4 | 1-[4,4-Bis(4-fluorophenyl)butyl]piperidin-4-yl-1,3-dihydroindol-2-one |
| P #5 | 1-(1-Butyl-4-piperidinyl)-2H-benzo[d]imidazol-2(3H)-one |
| P #6 | 1-[4,4-Bis(4-fluorophenyl)butyl]-1H-benzo[d]imidazol-2(3H)-one |
| P #7 | 3-[1-[4,4-Bis(4-fluorophenyl)butyl]piperidin-4-yl]-1(prop-2-ynyl)-benzo[d]imidazol-2(3H)-one |
| P #8 | 3-[1-[3-[2-[2[2-[2-[5-[(3aS,4S,6aR)-2-Oxohexahydro-1H-thieno[3,4-d]imidazol-4-yl]pentanamidyl]ethoxy]ethoxy]ethoxy]ethyl]-1,2,3-triazol-5-yl]methyl]-8-[4,4-Bis(4-fluorophenyl)butyl]-1-phenyl-1,3,8-triazaspiro[4.5]decan-4-one |
| P #9 | 8-[4,4-Bis(4-fluorophenyl)butyl]-1-phenyl-3-prop-2-ynyl-1,3,8-triazaspiro[4.5]decan-4-one |
| P #10 | 3-[1-[1,1-Bis(4-fluorophenyl)methyl]piperidin-4-yl]-1H-benzo[d]imidazol-2(3H)-one |
| P #11 | 1-(1-(4,4-Bis(4-fluorophenyl)butyl)piperidin-4-yl)-4-hydroxy-1,3-dihydro-2H-benzo[d]imidazol-2-one |
| P #12 | 6-Methoxy-(piperidin-4-yl)-1H-benzo[d]imidazol-2(3H)-one |
| P #13 | 1-(1-(2-4-Fluorobenzyl-5-fluorophenoxy)propan-2-yl-piperidin-4-yl)-1H-benzo[d]imidazol-2(3H)-one |
| P #14 | 1-(1-(2-Benzylphenoxy)propan-2-yl-piperidin-4-yl)-1H-benzo[d]imidazol-2(3H)-one |
| P #15 | 4,4-Bis(4-fluorophenyl)butan-1-ol |
