# Supplementary material for: Uncertainty-aware quantitative analysis of high-throughput live cell migration data
Source: PLoS Comput Biol. 2026 Jul 13;22(7):e1014472. doi: 10.1371/journal.pcbi.1014472 (PMC13387618; doi:10.1371/journal.pcbi.1014472)
Supplement: S2 Table — Error counts were derived from a representative subset of 9 movies (20 frames each; 180 frames total). Automated tracking results were compared against manually corrected annotations after applying a minimum track-duration filter (≥10 frames per track). The table reports acyclic oriented graphs matching (AOGM)-inspired error counts following the implementation in MMV_H4Tracks (https://github.com/MMV-Lab/mmv_h4tracks), including false-positive detections (fp), false-negative detections (fn), object-splitting operations (sc), edge-deletion operations (de), edge-addition operations (ae), and total number of cell instances (N). For the corresponding sensitivity analysis of the downstream migration readout, 354 tracks were identified before correction and 360 after. The difference in average velocity before and after correction was negligible (difference = 0.003; 95% confidence interval = -0.028, 0.034; p-value = 0.85, Welch Two Sample t-test). Statistics across movies include Average (AVG), Standard Deviation (STD), and Average Error Percentage with Standard Error (SE). (PDF) [file pcbi.1014472.s027.pdf]

Supplementary information

S2 Table: Tracking-error summary based on manual frame correction

Movie	fp	fn	sc	de	ae	N
M1	2	3	0	0	3	504
M2	1	44	0	0	56	527
M3	5	20	0	2	24	332
M4	15	24	0	10	31	1205
M5	6	64	0	1	71	967
M6	39	1	0	19	3	1069
M7	4	9	0	3	15	924
M8	1	4	1	1	8	1181
M9	80	5	0	52	9	405
AVG	17	19.33	0.11	9.77	24.44	
STD	26.52	21.72	0.33	17.02	24.30	
AVG error %; SE	3.1; 2.11	2.91; 1.06	0.01; 0.01	1.84; 1.39	3.69; 1.25	
