# Supplementary material for: Uncertainty-aware quantitative analysis of high-throughput live cell migration data
Source: PLoS Comput Biol. 2026 Jul 13;22(7):e1014472. doi: 10.1371/journal.pcbi.1014472 (PMC13387618; doi:10.1371/journal.pcbi.1014472)
Supplement: S3 Table — Each prior is specified as a normal distribution N(M,SD) where M denotes the mean (_M) and SD denotes the standard deviation (_SD). Superscript + indicates half-normal priors (truncated at zero) for scale parameters. (PDF) [file pcbi.1014472.s028.pdf]

Supplementary information

S3 Table: Prior hyperparameters for `gen_full()` and `gen_partial()`

Parameter	Component	Description
`prior_alpha_p_M`, `prior_alpha_p_SD`	Plate batch effects (α_p)	Mean and SD of the normal prior for plate-specific batch effects on the log-scale. Represents the baseline migration velocity of control treatments on each plate. Default: $\mathrm{N}(-0.5, 1.0)$.
`prior_kappa_mu_M`, `prior_kappa_mu_SD`	Gamma shape mean (μ_κ)	Mean and SD of the normal prior for the population mean of $\log(\kappa_w)$, where κ_w is the Gamma shape parameter for well w. Equivalent to the mean log inverse squared coefficient of variation. Default: $\mathrm{N}(1.5, 1.0)$.
`prior_kappa_sigma_M`, `prior_kappa_sigma_SD`	Gamma shape SD (σ_κ)	Mean and SD of the normal prior for the population standard deviation of $\log(\kappa_w)$ across wells. Captures heterogeneity in the shape parameter. Default: $\mathrm{N}^+(0, 1)$.
`prior_sigma_bio_M`, `prior_sigma_bio_SD`	Biological variability (σ_{bio})	Mean and SD of the half-normal prior for variability between biological replicates (plates). Captures variation in treatment effects across independent experiments. Default: $\mathrm{N}^+(0, 1)$.
`prior_sigma_tech_M`, `prior_sigma_tech_SD`	Technical variability (σ_{tech})	Mean and SD of the half-normal prior for variability between technical replicates (wells within plates). Captures measurement noise and well-to-well variation. Default: $\mathrm{N}^+(0, 1)$.
`prior_sigma_delta_M`, `prior_sigma_delta_SD`	Treatment effect variability (σ_δ)	Mean and SD of the half-normal prior for the standard deviation of the population of overall treatment effects. Default: $\mathrm{N}^+(0, 1)$. Used only in `gen_full()` to draw treatment-specific effects.
