# Supplementary material for: Uncertainty-aware quantitative analysis of high-throughput live cell migration data
Source: PLoS Comput Biol. 2026 Jul 13;22(7):e1014472. doi: 10.1371/journal.pcbi.1014472 (PMC13387618; doi:10.1371/journal.pcbi.1014472)
Supplement: Attachment — Submitted filename: Response_to_Reviewers.pdf [file pcbi.1014472.s029.pdf]

Point-by-point replies to reviewers' comments

Reviewer #1

This manuscript describes a new tool for the analysis of cell migration data ('cellmig'). This R package implements Bayesian hierarchical models to estimate treatment effects while accounting from noise in the data arising from technical or biological variation or batch effects. The authors test a number of experimental conditions to validate the use of cellmig to detect variation in differences in cell migration between experimental or biological replicates. The data are robust and cellmig illustrates where conventional tracking and statistical methods fall short. That said, there are some areas where the paper could be stronger.

Comment 1: the Discussion nicely lays out the current limitations of cellmig but unfortunately these likely preclude its adoption by a wider audience of biologists. Cellmig is currently optimized for use with pixel per frame unit measurements, but biological data should not be shown this way. Including a demonstration of the process of optimizing the priors in the Bayesian model for data collected with other units of measurement would be helpful.

Our reply: We agree with the reviewer's emphasis on biological relevance. In the revised manuscript, we explicitly demonstrate cellmig's compatibility with micrometer-per-minute units -- the standard for migration studies. To ensure robust prior optimization across diverse measurement systems, we provide clear guidelines for adapting Bayesian priors to different experimental setups. Crucially, we validate our prior distributions using two approaches: First, we show that our priors fully encompass the physiological range of mean velocities from 54 cell types measured in "The First World Cell Race" (10.1016/j.cub.2012.07.052). Second, we demonstrate through comprehensive analysis of our new application dataset that the prior choices derived from our benchmarking dataset remain effective when applied to real-world experimental conditions. This dual-validation approach confirms that biologists can confidently apply cellmig to their data without unit-conversion barriers while maintaining statistical rigor.

Comment 2: Focus on cell velocity is also a limitation. If the goal is to reduce noise to identify rare or subtle changes, these changes will only be crudely observed by changes in cell velocity on a population level.

Our reply: This remark by the reviewer accurately reflects the state of the art before

cellmig. With cellmig this is no longer true. The revised version demonstrates with diverse real data sets, including now a new 15 compound multi-dose dataset, that population-level velocity analysis with cellmig enables detection of subtle, noise-masked effects in large-scale compound screens. Moreover, we pointed out that the probabilistic model underlying cellmig can be extended to other metrics.

Comment 3: Aside from validation experiments, the manuscript would greatly benefit from a biological application or finding. Is there a test case where application of cellmig can identify a biological finding otherwise masked by noisy data?

Our reply: We have added a novel biological case study demonstrating cellmig's ability to uncover otherwise masked findings. As detailed in the new Section "Application of *cellmig* in chemical biology", we applied cellmig to a novel large-scale chemical screen with over 80,000 cells treated by 15 chemical modulators of cell migration, each tested at 5 concentrations. This analysis enabled the first quantitative Structure-Activity Relationship (SAR) study for phenotypic migration data at this resolution. Critically, cellmig successfully quantified subtle and complex modulation patterns, uncovering new chemical biology.
