# Supplementary material for: Uncertainty-aware quantitative analysis of high-throughput live cell migration data
Source: PLoS Comput Biol. 2026 Jul 13;22(7):e1014472. doi: 10.1371/journal.pcbi.1014472 (PMC13387618; doi:10.1371/journal.pcbi.1014472)
Supplement: Attachment — Submitted filename: Rebuttal_Response_to_Reviewers.pdf [file pcbi.1014472.s030.pdf]

Point-by-point replies to reviewers' comments

Reviewer #1

Major concern 1: Benchmarking and "state-of-the-art" positioning are currently misaligned with the comparisons shown. If the authors want to retain an "exhaustive/SOTA" framing, the benchmark needs to include stronger, field-standard approaches for nested high-content data, ideally chosen from methods with stable, open implementations so the comparison is transparent and reproducible. At a minimum, that typically means testing against multilevel/hierarchical baselines (e.g., mixed-effects modeling, or Bayesian multilevel regression implemented in widely used frameworks such as Stan interfaces like brms), and also considering distribution-robust options that respect nesting (e.g., resampling approaches designed for clustered data). The high-throughput screening literature also provides relevant comparators and evaluation concepts that could strengthen the benchmarking story—plate QC metrics like *Z′*, spatial normalization approaches such as B-score/median polish, and established Bioconductor tooling for plate-based or image-based screens (e.g., cellHTS2, imageHTS). In parallel, there is now precedent for benchmarking batch-correction methods directly in image-based profiling (Cell Painting) using quantitative metrics for both batch removal and biological signal preservation; if the manuscript claims robustness to batch effects, this kind of framing can help define what "robust" means and how to measure it. Alternatively, if the authors' real target is to improve on common practice in migration workflows (two-stage summaries plus conventional tests), they should say so clearly and consistently and retire the "state-of-the-art" benchmarking language. That reframing does not eliminate novelty: the defensible contribution is an end-to-end, migration-tailored hierarchical workflow that unifies biological effects and batch/plate effects in one generative model and pairs inference with simulation-based experimental planning.

Our reply: We thank the reviewer for this insightful critique and, in particular, for providing a precise framing of our work's contribution. We found the following statement from your comment to be an exceptionally accurate summary of our goals:

"That reframing does not eliminate novelty: the defensible contribution is an end-to-end, migration-tailored hierarchical workflow that unifies biological effects and batch/plate effects in one generative model and pairs inference with simulation-based experimental planning."

Our primary contribution is not the development of new statistical theory, but rather the operationalization of established hierarchical Bayesian methods into an accessible, domain-specific workflow for cell migration biologists. To address this, we have revised the manuscript to explicitly position *cellmig* as a tool that bridges the gap between rigorous statistical methodology and experimental practice. Specifically:

1. **We have reframed our contribution:** We have removed 'state-of-the-art' claims regarding the statistics itself throughout the manuscript. Instead, we emphasize that while general-purpose packages for statistical modeling exist, they require specialized

expertise in model specification, diagnostics, and interpretation that *cellmig* encapsulates.

2. **We have clarified and expanded our benchmarking scope:** We clarified in the Method section **"Evaluating *cellmig* against commonly used approaches"**, that our benchmark against non-parametric tests (Wilcoxon/Kruskal-Wallis) reflects the *current standard practice* in the cell migration community, which often ignores hierarchical structure. We agree that comparing against mixed-effects models is valuable; therefore, we added a new benchmark against a **simplified Bayesian model** that ignores nesting and batch effects. This isolates the value of the hierarchical structure itself, independent of the Bayesian vs. Frequentist debate. We now include Leave-One-Out Cross-Validation (LOO-CV) metrics to quantitatively demonstrate that the hierarchical model offers improved predictive accuracy compared to non-hierarchical alternatives, providing an objective measure of 'robustness' beyond simple concordance.

3. **We clarify *cellmig's* scope in the Introduction:** "To address this gap, we present *cellmig*, an R package that makes rigorous statistical analysis accessible for cell migration studies. *cellmig* automatically handles the complexity of high-throughput migration data-accounting for variation between individual cells, wells, and experimental plates-and returns treatment effect estimates with clear uncertainty measures. The package also includes simulation functionality to help researchers determine how many replicates are needed before running costly experiments. *cellmig* is designed for single-cell velocity data with hierarchical structure (cells nested within wells within plates) and control treatments for batch correction. It is not suitable for assays producing well-level summaries (e.g., wound closure gap area), population-level metrics (e.g., optical flow, collective migration), or experimental designs without replicate structure. This focused scope enables rigorous uncertainty quantification tailored to high-throughput live cell migration data. By integrating these features into an accessible tool, *cellmig* enables quantitative research and enhances reproducibility. After benchmarking on two independent experiments (about 30,000 cells from 20 biological conditions), we applied *cellmig* to a high-throughput discovery experiment (about 80,000 cells from 80 biological conditions), uncovering new chemical biology while validating statistical robustness."

Major concern 2: The manuscript sometimes labels disagreements with the baseline analysis as "false positives" and "false negatives" without an independent truth standard. On real datasets, this is stronger than the evidence supports and reads as if the authors are declaring their own method the ground truth. A cleaner approach is to describe these as discordant calls and then argue, mechanistically and statistically, why one approach is more appropriate for the experimental structure at hand. If the authors want to make error-rate statements, they should confine those to settings where truth is known (e.g., simulations that include realistic plate/well/cell nesting and batch effects) or to external validation. More broadly, the screening literature already offers a vocabulary for decision-making that can be more informative than "false positive/false negative versus my method," including effect-size and ranking metrics used for hit selection such as SSMD; positioning the decision layer in that ecosystem would strengthen the paper and make it more familiar to screening practitioners.

Our reply: We thank the reviewer for this insightful critique. We agree that claiming "false positives/negatives" on real experimental data implies a ground truth that is unavailable outside of simulations. We have thoroughly revised the manuscript to address this.

First, we have confined all "false positive/negative" and "true positive rate" terminology strictly to the simulation sections (**Results: "Experiment design by numerical simulation" and Supplementary Section S4**), where ground truth is known by design.

Second, in the context of experimental datasets, we now describe discrepancies between methods as "discordant calls" and "likely false discoveries." For example, **in the Discussion**, we revised the benchmarking summary to state that *cellmig* demonstrated "enhanced robustness in resolving discordant calls" and "reduced likely false discoveries arising from inflated sample sizes," rather than claiming it "avoided false negatives/positives." Similarly, we updated Supplementary Figure S15 to reflect "Discordant treatment pairs" rather than error types.

Third, as suggested by the reviewer, we have updated the Discussion to position *cellmig* within the existing ecosystem of high-throughput screening tools, noting established metrics like SSMD and Z-factors, and highlighting how *cellmig* extends these concepts by propagating uncertainty through hierarchical modeling rather than relying on point estimates. We also added **a new Methods section "Visualization and prioritization of treatment effects,"** and a **new vignette ("Large Screen Analysis with *cellmig*")** to provide practical guidance on using *cellmig* outputs for prioritization and decision making in small and large-scale screens.

Fourth, to address the request for a mechanistic and statistical argument regarding experimental structure, we added a benchmark against a simplified Bayesian model that ignores hierarchical nesting. This comparison **quantitatively** isolates the value of the hierarchical structure rather than treating it as a decision problem. We show that the non-hierarchical model produces (a) biased estimates of overall treatment effects because it disregards batch effects and (b) artificially narrow uncertainty intervals due to pseudoreplication, leading to prioritization decisions that differ from those of the hierarchical model. We describe these as "discordant calls arising from structural mismatch" rather than errors, emphasizing that the hierarchical model is statistically appropriate for the nested design. This analysis is presented in our **new Results section ("Comparison with simplified Bayesian model")** and supported by Leave-One-Out Cross-Validation (LOO-CV) metrics demonstrating improved predictive accuracy for the hierarchical structure.

Major concern 3: The paper emphasizes uncertainty and probability-style evidence summaries, but it does not yet make a convincing case that these quantities are calibrated or how they should be used to make screening decisions at scale. The Bayesian workflow literature is blunt on this point: uncertainty intervals are only as good as the model and need to be interrogated with posterior predictive checks and sensitivity analysis, especially when the data-generating process is plausibly misspecified. This matters here because velocity distributions can be skewed and heavy-tailed, and can also be “structurally weird” due to tracking dropouts and filtering rules. The paper would be much stronger if it demonstrated that

its uncertainty statements behave sensibly under realistic conditions and then explained, in practical terms, how an analyst should use those quantities to prioritize treatments in large screens (including how the approach interacts with multiple testing or multi-condition ranking).

Our reply: we thank the reviewer for this critical insight. We agree that uncertainty quantification is only valuable if the underlying model is robust, calibrated, and actionable for screening decisions. We have significantly revised the manuscript, highlighting model calibration, prior sensitivity/prediction analysis, and model validation by posterior predictive checks. We made the following specific changes:

Posterior predictive checks at the level of cells and wells: To directly address the concern about whether uncertainty statements behave sensibly under realistic conditions, we now include Posterior Predictive Checks (PPCs) for each of our datasets in the results section:

- **Dataset 1:** Section "Experimental design and computational workflow", Supplementary Figure S11 and S12
- **Dataset 2:** Section "Application of *cellmig* in chemical biology", Supplementary Figure S19C

In these figures, we compare the distribution of observed cell/well velocities against the distribution of velocities simulated from the fitted model parameters' posteriors. The close agreement confirms that the Gamma error model is appropriate and the hierarchical model structure is well-calibrated and accurately reproduces the observed data, including skewness and variability. This demonstrates that the 95% HDIs reported by *cellmig* are reliable estimates of uncertainty rather than artifacts of model misspecification. As posterior predictive checks are essential to diagnose invalid models and to detect "structurally weird" data, as pointed out by the reviewer, we have extended Supplementary Section S1 (Model inference and checks) to provide a transparent description of our model validation workflow.

Data inspection and model fit diagnostics: We have updated the main *cellmig* vignette (Vignette "*cellmig:* Quantifying Cell Migration Velocity with Hierarchical Bayesian Models", Section: "Model Diagnostics & Calibration") to guide users in interrogating their specific datasets. This includes:

- Visual inspection of raw velocity distributions (at the level of cells, wells and plates) to detect abnormalities such as multimodal velocity data
- Instructions for checking Pareto k diagnostics (using the *loo* package) to identify highly influential observations or model misspecification.
- Guidance on inspecting *rstan* objects to ensure MCMC convergence.
- Visual workflows for posterior predictive checks: comparing observed vs. predicted velocity distributions to detect invalid models.

Uncertainty Calibration: We added a new Results paragraph ("Uncertainty interval calibration") demonstrating that *cellmig*'s 95% HDIs achieve 95.1% coverage across all experimental configurations in our simulation, confirming statistical calibration.

Practical guidance for screening decisions: to address how researchers should use these quantities at scale we have **updated our hierarchical model to estimate the shrinkage hyperparameter (σ_δ) from the data rather than fixing it a priori.** We clarified in the **new Methods ("Treatment-level model: partial pooling across treatments", Equation 6)** and the **new Vignette** (**"Large Screen Analysis with *cellmig*"**) that this adaptive hierarchical shrinkage inherently controls false positives by pulling uncertain effects toward zero. This obviates the need for explicit multiple testing correction while maintaining rigorous error control that adapts to the specific screen's signal-to-noise ratio.

- **New vignette:** We created a new R vignette titled **"Large Screen Analysis with *cellmig*"**. This tutorial uses simulated data with **known ground truth** to demonstrate how uncertainty estimates behave under varying noise conditions. It explicitly shows a) how uncertainty interval widths are affected by artificially introduced variability in the experiment; and b) how partial pooling recovers true effects and controls false positives by partial pooling.
- **Prioritization framework:** We added a practical decision framework in the Results (**Visualization and prioritization of treatment effects**) and Discussion. We explain how to jointly use effect size (ρ') and probability of effect (π) to prioritize hits.

We believe these additions demonstrate that *cellmig*'s uncertainty statements are calibrated, and directly actionable for large-scale screening workflows, while at the same time equipping the user with the tools to detect data anomalies and instances where the model might be misspecified.

Major concern 4: Robustness to model misspecification and common migration-data artifacts needs more emphasis. Migration velocity estimates can be sensitive to outliers, track fragmentation, and heterogeneity that looks more like mixtures than a single clean distribution. The manuscript would benefit from explicitly acknowledging these failure modes and showing that conclusions are either stable to them or that the tool provides clear indicators when they are not. This is also where the existing ecosystem provides useful context: in many migration settings, investigators rely on mature tracking and analysis toolchains (e.g., TrackMate, u-track, CellProfiler) and the broader community has developed benchmark standards for tracking performance (e.g., the Cell Tracking Challenge). If the paper's downstream inference depends heavily on tracking-derived velocities, it should engage with that reality and help users understand when measurement artifacts could dominate the inference.

Our reply: We thank the reviewer for this helpful comment. We agree that tracking-derived migration velocities can be affected by common tracking artifacts, including detection errors, linking errors, and track fragmentation.

To address this point, we have added a tracking-error summary based on the graph-edit operations underlying the AOGM measure used in the Cell Tracking Challenge [1]. Since the full AOGM measure is defined as a weighted sum of these operations, we do not report it as a single score. Instead, following the implementation in [2] we have provided the individual error

counts, as these are more directly interpretable for assessing detection- and linking-level artifacts that may affect downstream velocity estimates.

To obtain these error counts, we manually corrected a representative subset of 9 movies (M1-M9) with 20 frames each, corresponding to 180 frames in total, and compared the automated tracking output against these corrected annotations after applying our noise-reducing minimum track-duration filter requiring at least 10 frames per track. The results of this analysis are shown in Table 1, which is now included as **Supplementary Table S2** in our revised manuscript. In short, this analysis identified 153 false-positive detections (fp), 174 false-negative detections (fn), 1 object-splitting operation (sc), 88 edge-deletion operations (de), and 220 edge-addition operations (ae). False-positive and false-negative detections represent spurious and missed cell detections, respectively. Edge-deletion operations correspond to redundant or incorrect temporal links, whereas edge-addition operations correspond to missing temporal links between detections. The object-splitting operation indicates one case in which a detected object had to be separated into multiple cell instances. Next, we calculated the total number of cell instances (N) in each movie, which allowed us to calculate the relative count of each error type (error/N). Across the curated movies, the mean relative error percentage for all types was below 3.69%, with low standard errors. These absolute and relative counts provide transparency regarding the extent and nature of tracking artifacts.

In addition, we have included a brief sensitivity analysis of the downstream migration results before and after frame correction in this subset. Specifically, we have reported the number of tracks and the difference in average speed **before correction** (354 tracks, mean velocity = 0.441 µm/min) and **after correction** (360 tracks, mean velocity = 0.438 µm/min): negligible difference in velocity = 0.003 (95% confidence interval: -0.028, 0.034; Welch Two Sample t-test, p-value = 0.85), suggesting that the main migration readouts are not substantially affected by the observed level of tracking artifacts.

movie	***fp***	***fn***	***sc***	***de***	***ae***	***N***
M1	2	3	0	0	3	504
M2	1	44	0	0	56	527
M3	5	20	0	2	24	332
M4	15	24	0	10	31	1205
M5	6	64	0	1	71	967
M6	39	1	0	19	3	1069
M7	4	9	0	3	15	924
M8	1	4	1	1	8	1181
M9	80	5	0	52	9	405
**AVG**	17	19.33	0.11	9.77	24.44	
**STD**	26.52	21.72	0.33	17.02	24.30	
**AVG error % (SE)**	3.1 (2.11)	2.91(1.06)	0.01 (0.01)	1.84 (1.39)	3.69 (1.25)	

Table 1: Summary of error counts from manual curation of 9 movies: M1-M9 (movie name=column movie). Column legends: fp=false positives; fn=false negatives; sc=object splitting operations; de=edge deletion operations; ae=edge addition operations; N=total number of cell instances; Statistics: Average (AVG), Standard Deviation (STD), and Average Error Percentage (AVG error %) with Standard Error (SE).

In addition to these image-processing quality controls, we have augmented *cellmig* (the R-package), the vignette, and the manuscript with statistical diagnostics that allow users to detect anomalous velocity distributions, highly influential outliers, and other types of abnormalities that might cause the inference to break. As described in our response to Major Concern 3, we now emphasize posterior predictive checks (PPCs) in the Results and Supplementary Section S1, which compare observed velocity distributions against simulations from the fitted model to reveal systematic deviations such as multimodality or excess zeros indicative of tracking dropouts. Furthermore, the vignette now includes detailed guidance on inspecting MCMC convergence diagnostics (R-hat, effective sample size, divergences) to ensure uncertainty estimates are not artifacts of poor sampling, and on performing pointwise inspection using Leave-One-Out (LOO) Pareto k diagnostics. High k values (>0.7) flag cells that are highly influential or poorly fit by the model, often signaling tracking artifacts or biological outliers that require investigation.

This layered approach (image processing quality controls and inference-level statistical diagnostics) ensures robust conclusions, directly addressing the concern about robustness to model misspecification and common migration-data artifacts.

[1] Matula, Pavel, et al. "Cell tracking accuracy measurement based on comparison of acyclic oriented graphs." PloS one 10.12 (2015): e0144959.
[2] https://github.com/MMV-Lab/mmv_h4tracks

Major concern 5: The manuscript should more clearly delimit what is modeled and what is not with respect to upstream image analysis. Right now, the inference layer risks being read as more “uncertainty-aware” than it truly is, because segmentation/tracking uncertainty is not explicitly modeled and filtering choices can change the effective population being analyzed. The most convincing way to handle this is straightforward: state the scope clearly, then show sensitivity analyses that demonstrate how robust the reported treatment effects are to plausible variations in preprocessing and QC. The paper would also benefit from stronger evidence of measurement fidelity (clearer overlays of raw images with segmentation and tracks, and reporting of basic QC summaries such as track-length distributions and exclusion rates), so that readers can judge whether the velocity measurements are trustworthy inputs to a hierarchical inference engine.

Our reply: We thank the reviewer for this important clarification regarding the scope of uncertainty quantification in *cellmig*. We agree that it is essential to clearly delimit what sources of uncertainty are modeled versus what are treated as fixed inputs, and to ensure users understand that valid inference requires quality-controlled data. To address this, we have revised the introduction to the **Materials and Methods section** to include an explicit scope definition at the beginning of the statistical modeling subsection. This statement clarifies that *cellmig* focuses on statistical inference from quality-controlled and processed cell migration data rather than image-processing uncertainty, specifying that the tool does not perform image processing and does not quantify uncertainty arising from segmentation, tracking, or other upstream image analysis and quality control steps. We further emphasize that users must

ensure input data have undergone rigorous quality control, as valid statistical inference relies on the accuracy of these upstream measurements. This addition helps readers to understand from the outset that *cellmig* operates downstream of image analysis and treats velocities as fixed inputs, preventing misinterpretation of the tool as an end-to-end image analysis pipeline.

In parallel, we have updated the Limitations paragraph in the **Discussion** to explicitly acknowledge that *cellmig* quantifies uncertainty at the statistical inference level (biological/technical variability, batch effects) but does not model measurement uncertainty from upstream segmentation and tracking. We explain this as a deliberate design choice that ensures compatibility with diverse tracking workflows and accessibility for experimental biologists, while noting that future work may integrate measurement uncertainty models for users requiring this additional layer of rigor.

To demonstrate that our conclusions remain robust despite this scope limitation, we refer to the sensitivity analyses and quality control evidence described in our response to Major Concern 4. These include robustness checks across preprocessing variations (track-length filters, cell-count thresholds), raw image overlays with segmentation tracks, and track-length distributions. Together, these textual clarifications and empirical validations ensure that readers cannot misinterpret "uncertainty-aware" as including measurement uncertainty, understand that quality-controlled data is a prerequisite for valid inference, and have the tools to assess whether their data meets quality thresholds before analysis.

Minor concern 1: Figure clarity, especially for segmentation/tracking examples, should be improved so readers can actually evaluate measurement fidelity. If space is tight in the main text, the supplement is fine, but the examples need to be legible and representative.

Our reply: We agree that transparent visual examples are essential for evaluating measurement fidelity. We have included a **new Supplementary Figure S10** to include high-clarity, representative examples of raw microscopy frames overlaid with segmentation masks and track identifiers. These qualitative examples now directly complement the quantitative error metrics in the **newly included Supplementary Table S2.** Furthermore, we have updated the Methods section ("Computational segmentation and tracking") to explicitly reference these improvements. Together with our sensitivity analysis showing negligible impact on velocity estimates, these updates provide robust evidence of tracking fidelity.

Minor concern 2: The manuscript should ensure consistent handling of fold-change null references. If the null is 1 on the natural scale and 0 on the log scale, that needs to be stated and used consistently across axes, captions, and legends to avoid confusion.

Our reply: We thank the reviewer for this important clarification. We have revised the manuscript to ensure strict consistency in how effect scales and null references are presented. Specifically:

We added a clear statement in the Methods section (**"Quantifying treatment effects"**) that reads: *"Throughout the Results and Figures, we present effects on the natural scale (fold-change), where the null effect equals 1 (no change). Values greater than 1 indicate increased migration velocity relative to control; values less than 1 indicate decreased migration velocity."*

We have standardized the text to use δt′ and ρ′ (exponentiated values) throughout the Results section, removing ambiguous references to the log-scale null (0).

We updated all relevant figure captions (Figures 2, 3, 5, and 7) to consistently reference the null effect as "1 (no change)." Specifically, we revised **Figure 5 (Panels A–D)** to display fold-change (ρ′) rather than log-fold-change (ρ), ensuring that all benchmarking visuals align with the natural scale used in the rest of the manuscript.

Minor concern 3: A short scope statement would help. Readers should know exactly which migration paradigms the method targets (single-cell velocity summaries in high-throughput, plate-based assays) and which common paradigms it is not aiming to replace (e.g., scratch/wound-closure analysis pipelines such as TScratch, broader end-to-end migration environments like CellMissy, or collective-migration workflows that use optical-flow/PIV rather than single-cell tracking). This also helps prevent overgeneralization of "velocity" across biological regimes.

Our reply: We thank the reviewer for this suggestion. We agree that clearly defining the scope of *cellmig* is essential to prevent overgeneralization and help users identify when the tool is appropriate for their experimental design. We have added explicit scope statements in two key sections:

We added a paragraph at the end of the **Introduction** clarifying that *cellmig* focuses on statistical analysis of single-cell velocities from hierarchical, plate-based assays. We explicitly state that it is *"not suitable for assays producing well-level summaries (e.g., wound closure gap area), population-level metrics (e.g., optical flow, collective migration), or experimental designs without replicate structure."*

We reinforced this scope in the **Discussion**, specifying that *cellmig "is not designed for wound-closure assays or collective-migration workflows."*

Minor concern 4: Reproducibility and deployment details should be tightened. Readers will expect basic guidance on versions, typical compute requirements, and a minimal end-to-end script or vignette that reproduces a primary analysis.

Our reply: We thank the reviewer for this important suggestion. We have strengthened the reproducibility and deployment details in three ways:

1. **Version Information**: We added a new "Software availability" subsection in the Methods specifying the version of *cellmig* and key dependencies.

2. **Compute Requirements:** We added a **new "Computational requirements"** section in the Results benchmarking elapsed time and peak RAM across typical experimental configurations. We now explicitly reference our experimental datasets as concrete examples: Dataset 1 (20 treatment groups, 8 biological replicates, 4 technical replicates; 338 wells, 29,503 cells) completed in approximately 15 minutes; Dataset 2 (80 treatment groups, 18 biological replicates, 3-4 technical replicates; 1,109 wells, 84,122 cells) completed in approximately 1 hour. Our benchmarks show that *cellmig* is computationally accessible on standard personal computers for typical experimental designs (25-50 treatments, 3--6 replicates complete within 40 minutes using <7 GB RAM per chain), while larger screens may benefit from server access.

3. **Reproducible Vignette**: We provide comprehensive scripts reproducing the analysis of Dataset 1 and Dataset 2 at https://github.com/snaketron/cellmig_paper_data, with additional vignettes for analysis and simulation on Bioconductor. These have now been extended to include executable code, expected outputs, and workflow guidance. These resources are described in the **newly added section "Software availability"**.

Reviewer #2

Major Comment 1 (General presentation): I find the manuscript which confusing to read its current layout. It actually feels like the work was originally written for a Journal with section orders of Introduction -> Results -> Discussion -> Materials and Methods (e.g., like many general science journals). I found the transition from Introduction to Materials and Method to be incredibly jarring with the Materials and Methods being full of undefined acronyms and jargon, most of which was not at all properly defined until the Results section.

Our reply: We thank the reviewer for this critical observation regarding the manuscript structure and readability. We agree that the original organization created an abrupt transition by introducing technical statistical notation before establishing the experimental context, and that several technical terms were not defined until later sections.

To address this, we have completely restructured the Materials and methods section with the following specific changes:

1. **Reorganized logical flow:** We have restructured the Materials and Methods section to follow a more intuitive narrative. It now begins with an overview of the Cell Migration Data (describing the experimental strategy and dataset structure), followed by the Statistical Modeling (our main computational contribution, *cellmig*), and concludes with the Experimental Data Generation protocols. This ensures readers understand the data structure and analytical approach before encountering detailed laboratory protocols.
2. **Defined all abbreviations and removed jargon:** We have defined all technical terms and abbreviations at first use (e.g., HDI, CV, DMSO, ASPC1, etc.) and added explanatory phrases for statistical/experimental concepts to ensure accessibility for non-specialist readers.
3. **Added workflow overview and introduction:** We have added a concise introductory paragraph to the Materials and Methods section that explicitly outlines the section's structure. Additionally, we have created a new workflow figure (**Figure 2**) placed at the beginning of this section. This figure details the main steps of our experimental and computational pipeline in a digestible way, guiding the reader through the subsequent subsections.

Major Comment 2 (Contribution): It is difficult for me to identify what the main contribution to paper is presenting. Is it the cellmig package itself? or is it the modelling and the demonstration of the importance of dealing with technical/biological heterogeneity in a hierarchical sense? A clear contribution statement in the introduction would benefit the paper substantially. There also needs to be more clarity in exactly what the cellmig R package is providing. For example, if the cellmig restricted to the hierarchical model presented? What does it provide that other Bayesian tools for say Generalised linear/non-linear mixed-effects models would provide (like BRMS). The model that is presented in Equations (1)--(11) is not novel in of itself, so this all needs more clarity for me. Finally, the claim about the generative features within cellmig is not a contribution really, as this is an automatic feature of any parametric model

(Bayesian or not), and the authors concepts of fully generative and partially generative is effectively equivalent to prior and posterior predictive sampling which is standard practice. To be clear, I am not saying that this is not a valid thing to do, but the claims of novelty here are tool strong.

Our reply: We thank the reviewer for this constructive feedback. We agree that our original manuscript did not clearly distinguish between statistical novelty and practical implementation. We have revised the manuscript throughout to clarify that our primary contribution is accessibility and standardization of established methods for a specific domain, rather than the development of new statistical theory. Specific changes were made in:

Abstract: We tempered language implying statistical novelty. We now describe *cellmig* as a "specialized computational tool" that "implements established Bayesian hierarchical modeling within an accessible workflow tailored for cell migration assays."

Introduction: We completely restructured this section into three paragraphs to clearly articulate the contribution:

- Paragraph 1: Establishes the problem (current methods are inadequate).
- Paragraph 2: Introduces the Bayesian alternative and explicitly acknowledges that general-purpose frameworks exist (e.g., *brms*), but explains that they demand specialized expertise (specifying models for noise/batch correction, diagnosing convergence, organizing inputs, visualizing results).
- Paragraph 3: Positions *cellmig* as the solution that "encapsulates this expertise within a domain-specific workflow," making rigorous analysis accessible without requiring custom code.

Methods: We added an introductory statement acknowledging that "the hierarchical Bayesian framework presented here follows established statistical principles (Gelman, 2013 [1], Bürkner, 2017 [2]). Our contribution lies in tailoring this framework to cell migration data structures and providing an accessible implementation for experimental biologists."

Methods (Experiment Planning by Simulation): We clarified the terminology around "generative" modes. We now state: "Both modes are variants of prior predictive sampling (Gelman, 2013), with the latter allowing users to incorporate domain knowledge by constraining specific parameters." This acknowledges standard Bayesian practice while explaining the utility of our two modes.

Discussion: We added a new paragraph explicitly acknowledging that "the underlying hierarchical Bayesian framework is well-established and could theoretically be implemented using general-purpose packages like *brms*." We clarify that *cellmig's* value is removing barriers (model specification, convergence diagnostics, interpretation) that prevent biologists from using these rigorous methods.

We revised "unique strength" to "practical feature" and acknowledged that "prior predictive sampling is standard in Bayesian analysis (Gelman, 2013)" emphasizing that our contribution is integrating this into a simple function for experimental design.

We believe that these revisions accurately position *cellmig* as a valuable resource for the cell migration community. Our goal is not to introduce new statistical theory, but to operationalize existing rigorous methods into a standardized, accessible workflow that lowers the technical barrier for experimental biologists. This enables researchers to benefit from uncertainty-aware hierarchical modeling without needing to master the underlying statistical implementation and allows them to quantitatively analyze their high-throughput data.

[1] Gelman, Andrew, et al. *Bayesian data analysis*. Chapman and Hall/CRC, 1995.
[2] Bürkner, Paul-Christian. "brms: An R package for Bayesian multilevel models using Stan." *Journal of statistical software* 80 (2017): 1-28.

Major Comment 3 (Modelling): I am generally comfortable with the modelling itself, but I think the choices around priors are not clear at all. The section titled ``Model priors'' on page 10 state ``Wherever possible, we used priors informed by the literature, otherwise employing weakly informative priors ... ' ' however, there is not a single reference to the literature in this section, nor would I consider a normal prior with variance of 1 to be weakly informative, especially for a scale parameter. The Supplementary figures referred to are useful, but they are not justification for the prior choice alone. The priors obtained form the literature should be justified by clear reference to the relevant literature and the claim of weakly informative priors should also be justified (e.g., Gelman text book cited elsewhere discusses this quite a bit in the context of the schools example).

Our reply: We thank the reviewer for this critical feedback. We agree that the original description of our prior choices required further justification and precise terminology. We have substantially rewritten and restructured the "**Model priors**" section to address these points:

Terminology Correction: We have replaced the term "weakly informative" with "regularizing priors", following the terminology and guidance in Gelman et al. (2013) [1]. We now explicitly cite this reference within the section to justify the use of Half-Normal(0,1) priors for scale parameters.

Explicit Literature References: We have expanded the justification for our location parameters (α_p, μ_κ) by explicitly referencing two key literature sources:

1. Maiuri et al. (2013, World Cell Race study [2]): Used to inform the range of plausible velocities across 54 distinct cell types.
2. Cibir et al. (2023) [3]: Used to validate the priors against independent high-throughput neutrophil data. Since neutrophils migrate an order of magnitude faster than tumor cells, this confirmed that our priors are flexible enough to accommodate different biological contexts without being overly restrictive.

Prior Predictive Checks with Different Configurations: To address the concern about the restrictiveness of the priors, we performed a prior predictive check analysis using different prior configurations. Specifically, we compared Half-Normal(0, 1) against wider configurations (e.g., Half-Normal(0, 3)). We confirmed that while posterior estimates remain stable, the wider prior configurations generate biologically implausible velocities (over 5% of simulated cells had velocity >100 µm/min) in the prior predictive distribution. This demonstrates that our chosen configuration balances flexibility with biological plausibility. This analysis is now described in the text and detailed in the **revised Supplementary Figure S3**.

Detailed Parameter Justification: We added dedicated paragraphs for key parameters (specifically σ_δ and the scale parameters) to explain the biological and empirical reasoning behind their ranges, such as the sparsity assumption that most compounds have small effects ($|\delta_t| < 1$).

User Flexibility: We have clarified in the manuscript that while we provide default regularizing priors based on literature and empirical data, all prior hyperparameters are fully adjustable within the *cellmig* package as described in the **new Supplementary Sections S2, S3 and S5,** as well as the **new Supplementary Table S3**. This allows users to incorporate specific domain knowledge or adjust for different experimental contexts if needed.

We believe these revisions provide the transparency and rigorous justification required for our Bayesian framework.

[1] Gelman, Andrew, et al. *Bayesian data analysis*. Chapman and Hall/CRC, 1995.
[2] Maiuri, Paolo, et al. "The first world cell race." *Current Biology* 22.17 (2012): R673-R675.
[3] Cibir, Zülal, et al. "ComplexEye: a multi-lens array microscope for high-throughput embedded immune cell migration analysis." *Nature Communications* 14.1 (2023): 8103.

Major Comment 4 (Results): There are two main concerns about the results: **Major Comment 4.1:** The comparison between the probability of differential effect on migration π given in Equation (8) and the outcome of a frequentist hyponthesis test using a non-parametric test. This is statistically invalid from my perspective as Bayesian methods do not have statistical converage guarentees at all and the definition of π is simply not comparable to a p-value. Furthermore, a comparison of non-parametric methods with a parametric model is also not valid. To properly evaluate the improvements of cellmig, an equivalent Bayesian model that ignores the hierarchical structure would be needed. Such a comparison would ideally include prior sensitivity analysis.

Our reply: We thank the reviewer for this thoughtful critique. We agree with both points raised and have substantially revised our analysis in response.

1. Comparison of π vs. p-values: We fully acknowledge that π and p-values are fundamentally different quantities with different statistical interpretations, and direct comparison is not statistically valid. The purpose of our benchmark is not to equate π with p-values, but rather to compare decision-making frameworks that researchers in this field actually use in practice. In large-scale screens (e.g., with >1,000 treatments), experimental biologists often must make decisions about which compounds to validate experimentally based on the results

(differences in treatment effects) from the initial screen. The current standard practice is to use frequentist methods with a p-value threshold (e.g., $p<0.05$) to declare effects "significant." Our Bayesian approach provides an alternative decision-making framework based on probabilities of effect (π). We are comparing the practical outcomes of these two decision frameworks—specifically, which treatments each approach would prioritize for follow-up—not the statistical metrics themselves. We have revised the manuscript section to clarify our intent. We have added explicit language throughout the Results and Discussion to prevent misinterpretation.

2. Bayesian model comparison (new analysis): Following the reviewer's recommendation, we have now added a direct comparison between our hierarchical Bayesian model and a simplified Bayesian counterpart that ignores the nested experimental structure and batch effects. This addresses the core methodological concern more appropriately than the frequentist comparison alone. Key findings from this new analysis:

- Both models yielded similar posterior means for overall treatment effects (δ_t'), but the uncertainty estimates diverged substantially (**updated Figure 5C**). The simplified model produced artificially narrow 95% HDIs due to pseudoreplication (pooling all cells receiving the same treatment) and the inability to account for plate-to-plate variability. Furthermore, we observed systematic shifts in treatment effects corresponding to experiments A and B, reflecting unmodeled batch effects, which propagated to biased differences in overall treatment effects between pairs of treatments.
- The underestimation of uncertainty propagated to pairwise comparisons (Figure 5D), generating spurious differences between treatment groups. The simplified model identified 30 additional treatment pairs with high probability ($\pi \geq 0.99$) of differential effect compared to *cellmig*'s hierarchical model. Meanwhile, unmodeled batch effects created systematic discrepancies in effect magnitude between the two models,visible as red dots above the diagonal in Figure 5D (inter-experiment comparisons).
- Quantitative model comparison based on Leave-One-Out Cross-Validation strongly favors the hierarchical model (ΔLOOIC=861.1, SE = 45.9)

This analysis is now presented **in a new subsection "Comparison with simplified Bayesian model" (Results)**. Full specifications of both models are now provided in the **newly created Supplementary Section S2 and S3**.

3. Prior Sensitivity Analysis: The results of our new prior sensitivity analysis (Half-Normal(0, 1) vs. Half-Normal(0, 3) on scale parameters for both *cellmig* model and the simplified Bayesian model) are included in the **newly created Supplementary Figure S4**. Posterior estimates remained stable across both configurations, with negligible differences in parameter means. This confirms that inferences are data-dominated rather than prior-driven. Consequently, the observed differences in uncertainty quantification between the hierarchical and simplified Bayesian models reflect structural differences in accounting for data hierarchy, rather than artifacts of prior specification.

We hope this clarification accurately conveys our intent: to provide experimental biologists with a more reliable decision-making tool for prioritizing treatments in large-scale screens, not to establish statistical equivalence between Bayesian and frequentist metrics.

Major Comment 4.2: The experimental design results and associated claims are potentially extremely biased as the simulations are effectively posterior predictive samples. So it is unclear how generalisable these results are. I wonder if a prior predictive approach would be better to highlight the value of the cellmig tools.

Our reply: We thank the reviewer for raising this important distinction between prior predictive checks and simulation-based experimental design. We agree that this distinction is critical for interpreting the generalizability of our results. We wish to clarify that our simulation framework serves two distinct purposes, each requiring a different generative approach.

First, to ensure the model itself is generalizable and not biased toward our specific dataset, we perform **fully generative** simulations (*cellmig*'s function `gen_full()`) where all parameters are drawn from priors. As the reviewer suggested, this is the correct approach to validate that the model can accommodate diverse biological contexts (e.g., different cell types or migration speeds). We have included these checks in **Supplementary Figure S3**, demonstrating that our priors generate biologically plausible velocities across a wide range of scenarios (from slow epithelial cells to fast neutrophils).

Second, to provide actionable guidance for experimental planning, one must make assumptions about the variance structure of the specific assay being used. Power analysis inherently requires estimates of effect sizes and noise levels (e.g., variability between biological and technical replicates: σ_{bio} and σ_{tech}). Therefore, for **Figure 7 (Figure 6 in the initially submitted manuscript)**, we used a **partially generative** approach (*cellmig*'s function `gen_partial()`), fixing variance parameters to empirical estimates derived from our high-throughput migration assays.

The reviewer is correct that the specific power curves in Figure 7 are contingent on the noise levels observed in our specific experimental setup (laboratory, imaging system, cell line, and tracking pipeline). If a user employs a different setup (e.g., a much noisier microscope or a more heterogeneous cell type), the specific curves in Figure 7 would not directly apply. However, this is not a limitation of the tool, but a fundamental property of power analysis.

This workflow ensures that the tool remains generalizable: it does not impose our noise assumptions on others, but rather provides the mechanism for users to incorporate their own noise estimates into the planning process. To ensure this distinction is unmistakable to readers, we have made the following revisions:

- Methods (subsection "Experiment planning by simulation"): We expanded the description of the two simulation modes. We now explicitly state: "The specific power curves presented in the Results reflect the noise structure of our assay. For experimental setups with different variability (e.g., different imaging systems), users should estimate variance components from a pilot study and use the partially generative mode to tailor the power analysis to their specific context.".
- Results (subsection "Experiment design by numerical simulation"): We added a clarifying sentence noting that while biological replicates dominated uncertainty reduction in our

data, cellmig enables power analysis under diverse conditions (e.g., high variability regimes) where technical replicates may have greater influence.

- Discussion: We revised the paragraph on simulation capacity to highlight that users must balance specificity (using pilot data for power analysis) and generalizability (using vague priors for model validation), directly addressing the concern about bias.
- Figure 7 Legend: We added a note specifying that the simulations used variance parameters inferred from Dataset 1, and that users should adjust these parameters for different experimental conditions."

Major Comment 5 (Software): I was a bit surprised given the emphasis on the cellmig package that there was not clear description of it anywhere. I think the paper would benefit from a short appendix/supplementary description with example code demonstrating the main features of the software.

Our reply: We thank the reviewer for this valuable suggestion. We acknowledge that, despite the emphasis on the *cellmig* package in the main text, a dedicated description of its functionality and workflow was lacking. To address this, we have added a **new Supplementary Section S5: *cellmig* functions** to the supplementary document.

This section provides a comprehensive overview of the package's main functions, organized into four categories: (i) model fitting, (ii) posterior predictive checks, (iii) generative simulation, and (iv) visualization and comparison of treatment effects. For each function, we provide detailed descriptions of the main inputs and outputs, along with representative R code snippets demonstrating typical usage. Additionally, we have included a glossary table summarizing all prior hyperparameters used in the generative functions with explanations of their roles in the hierarchical model and default values. We believe this addition significantly enhances the reproducibility of our work and provides users with a clear, practical guide to utilizing the software.

Furthermore, we added a **Software availability** section in the Materials and Methods, which directs users to comprehensive scripts reproducing the analysis of Dataset 1 and Dataset 2 at https://github.com/snaketron/cellmig_paper_data. 3. In this section, we also reference two extended vignettes for analysis and data simulation available on Bioconductor (https://bioconductor.org/packages/cellmig/), which include executable code, expected outputs, and workflow guidance.

Minor comment 1: The next revision should include line numbers, it is quite difficult to provide minor feedback without line numbers.

Our reply: line numbers are included in the revised manuscript.

Minor comment 2: throughout, can Curiour New font be used for the cellmig software name.

Our reply: We appreciate this comment. Upon reviewing the PLOS Computational Biology author guidelines, we noted that while standard fonts are required, there is no strict mandate for monospaced fonts for software names. Furthermore, in surveying recent literature in the field, we observed that while practices vary, italics are commonly used to denote methods and software tools. To ensure the method name is highlighted consistently without implying it is a code snippet, we have chosen to use italics for *cellmig* throughout the manuscript.

Minor comment 3: The abstracts reference to the detail of the experiments are too vague. just remove them, that is the number of cells and conditions.

Our reply: We thank the reviewer for this suggestion. We have removed the specific numbers of cells and conditions from the abstract to improve clarity and conciseness, as suggested.

Minor comment 4: Page 3, can you provide references for the ``Diverse experimental methods…"

Our reply: We thank the reviewer for this suggestion. We agree that supporting references are necessary to substantiate the statement regarding experimental methods. We have now added a relevant citation (the review by Du et al., 2026 [1]) to this sentence in the revised manuscript (Page 3, Line 55).

[1] Du, W., Nair, P. R., Forjaz, A., Phillip, J. M., Wu, P. H., & Wirtz, D. (2026). Selecting the optimal cell migration assay: fundamentals and practical guidelines. *Nature methods*, *23*(1), 30-42.

Minor comment 5: Page 3, what you you mean by high-throughput?

Our reply: We thank the reviewer for highlighting this ambiguity. We realize that "high-throughput" can be interpreted in various ways. We have revised the text to specify that this refers to automated imaging using multi-well plate formats, which allows for the parallel analysis of dozens of conditions. The revised sentence reads:

“A high-throughput version of this method facilitates the analysis of cell velocity across dozens of wells in multiple multi-well plates via automated imaging and batch processing, enabling the extraction of quantitative velocities and the detection of changes following perturbations such as drug treatments (Cibir et al., 2023; Hulkower and Herber, 2011; Kramer et al. 2013).”

Minor comment 6: Page 4, I dont understand the point of the ``simple plots" as being a methodological issue, this is a communication issue.

Our reply: We agree with the reviewer that this point pertains more to data communication than to methodology. To avoid any confusion and to keep the focus strictly on statistical limitations, we have removed this sentence entirely from the revised manuscript. The text now focuses solely on the methodological gaps regarding hierarchical structure and uncertainty quantification.

Minor comment 7: Generally I find the methods section to be too vague. (e.g., what is the difference between A & B in dataset 1) especially page 4 to 7.

Our reply: We thank the reviewer for highlighting this lack of clarity. We agree that the distinction between Experiments A and B was not sufficiently explicit in the previous version. In the revised manuscript, we have restructured the Materials and methods section to address this vagueness.

Specifically, we now explain the distinction between Experiments A and B in the very first paragraph of the Cell migration data subsection. We clarify that these represent independent batches conducted with slightly different imaging configurations to intentionally introduce systematic batch effects, allowing us to benchmark the model's correction capabilities. The revised introductory paragraph now reads:

"To validate cellmig, we adopted a tiered experimental strategy comprising a benchmarking phase (Dataset 1) and an application phase (Dataset 2). ***Dataset 1 combines two independent experiments (A and B) designed to test the model's ability to handle technical variability. By conducting experiments A and B in independent batches with slightly different imaging configurations, we introduced systematic batch effects, which the model corrects using shared control treatments on each plate****. Dataset 2 serves as an independent, large-scale chemical screen to assess scalability and discover new biological insights. Detailed experimental protocols, including cell culture, compound preparation, and imaging parameters, are provided in the subsequent Experimental methods sections below. The following subsections detail the hierarchical structure of the processed data tables derived from these experiments."*

Minor comment 8: Page 10 remove the $\rho = $ in the integral lower bounds in equation (8).

Our reply: We have removed the rho from the integral lower bounds in Equation (8) as suggested.

Minor comment 9: Page 15, a Wilcoxon rank-sum test and Kruskal-Wallis text etc are not state-of-the-art, they are classical and standard methods, but not state-of-the-art.

Our reply: We agree that "state-of-the-art" was imprecise in this context. Wilcoxon and Kruskal-Wallis tests are indeed classical statistical methods, not methodological advances. Our intended meaning was that these represent the current standard practice in the cell migration analysis community, despite their limitations for hierarchical data. We have revised the manuscript throughout to replace "state-of-the-art" with more accurate phrasing such as "commonly used approaches," or "current standard practice". These changes are included in:

- Abstract:
  - old statement "Through exhaustive benchmarking **against state-of-the-art methods**, we demonstrate …"

    - new statement: “Through exhaustive benchmarking **against commonly used approaches in the field**, we demonstrate …”

- Section “**Evaluating *cellmig* against state-of-the-art approaches**” renamed to “**Evaluating *cellmig* against commonly used approaches**”
    - The following sentence was added in this section, referring to Wilcoxon rank-sum and the Kruskal-Wallis test, to avoid confusion: “These methods represent the **current standard practice in the field**, despite being originally developed for simpler experimental designs.”

We believe this better reflects our argument: that widely adopted methods in this field are inadequate for the data structure, not that they represent cutting-edge statistics.
