# Supplementary material for: Uncertainty-aware quantitative analysis of high-throughput live cell migration data
Source: PLoS Comput Biol. 2026 Jul 13;22(7):e1014472. doi: 10.1371/journal.pcbi.1014472 (PMC13387618; doi:10.1371/journal.pcbi.1014472)
Supplement: Peer review history

# Author comment: Author response to Decision Letter 0

**DOI:** 10.1371/journal.pcbi.1014472.r001

9 Feb 2026

Attachment Submitted filename: Response_to_Reviewers.pdf

# Peer review: Decision Letter 0

**DOI:** 10.1371/journal.pcbi.1014472.r002

**Authors:** Dimitrios Vavylonis

23 Mar 2026

PCOMPBIOL-D-26-00296

Uncertainty-aware quantitative analysis of high-throughput live cell migration data

PLOS Computational Biology

Dear Dr. Kitanovski,

Thank you for submitting your manuscript to PLOS Computational Biology. After careful consideration, we feel that it has merit but does not fully meet PLOS Computational Biology's publication criteria as it currently stands. Both reviewers find the method promising but request clearer articulation of the contribution, stronger benchmarking against appropriate methods, and improvements in presentation, modeling justification, and analysis. Therefore, we invite you to submit a revised version of the manuscript that addresses the points raised during the review process.

Please submit your revised manuscript by May 23 2026 11:59PM. If you will need more time than this to complete your revisions, please reply to this message or contact the journal office at ploscompbiol@plos.org. When you're ready to submit your revision, log on to https://www.editorialmanager.com/pcompbiol/ and select the 'Submissions Needing Revision' folder to locate your manuscript file.

Please include the following items when submitting your revised manuscript:

* A letter that responds to each point raised by the editor and reviewer(s). You should upload this letter as a separate file labeled 'Response to Reviewers'. This file does not need to include responses to formatting updates and technical items listed in the 'Journal Requirements' section below.

* A marked-up copy of your manuscript that highlights changes made to the original version. You should upload this as a separate file labeled 'Revised Manuscript with Track Changes'.

* An unmarked version of your revised paper without tracked changes. You should upload this as a separate file labeled 'Manuscript'.

If you would like to make changes to your financial disclosure, competing interests statement, or data availability statement, please make these updates within the submission form at the time of resubmission. Guidelines for resubmitting your figure files are available below the reviewer comments at the end of this letter

We look forward to receiving your revised manuscript.

Kind regards,

Dimitrios Vavylonis

Section Editor

PLOS Computational Biology

**Journal Requirements:**

If the reviewer comments include a recommendation to cite specific previously published works, please review and evaluate these publications to determine whether they are relevant and should be cited. There is no requirement to cite these works unless the editor has indicated otherwise.

1) We ask that a manuscript source file is provided at Revision. Please upload your manuscript file as a .doc, .docx, .rtf or .tex. If you are providing a .tex file, please upload it under the item type u2018LaTeX Source Fileu2019 and leave your .pdf version as the item type u2018Manuscriptu2019.

2) Please upload all main figures as separate Figure files in .tif or .eps format. For more information about how to convert and format your figure files please see our guidelines:

https://journals.plos.org/ploscompbiol/s/figures

3) We have noticed that you have uploaded Supporting Information files, but you have not included a list of legends. Please add a full list of legends for your Supporting Information files after the references list.

4) Some material included in your submission may be copyrighted. According to PLOS’s copyright policy, authors who use figures or other material (e.g., graphics, clipart, maps) from another author or copyright holder must demonstrate or obtain permission to publish this material under the Creative Commons Attribution 4.0 International (CC BY 4.0) License used by PLOS journals. Please closely review the details of PLOS’s copyright requirements here: PLOS Licenses and Copyright. If you need to request permissions from a copyright holder, you may use PLOS's Copyright Content Permission form.

Please respond directly to this email and provide any known details concerning your material's license terms and permissions required for reuse, even if you have not yet obtained copyright permissions or are unsure of your material's copyright compatibility. Once you have responded and addressed all other outstanding technical requirements, you may resubmit your manuscript within Editorial Manager.

Potential Copyright Issues:

i) Figure 1. Please confirm whether you drew the images / clip-art within the figure panels by hand. If you did not draw the images, please provide (a) a link to the source of the images or icons and their license / terms of use; or (b) written permission from the copyright holder to publish the images or icons under our CC BY 4.0 license. Alternatively, you may replace the images with open source alternatives. See these open source resources you may use to replace images / clip-art:

- https://commons.wikimedia.org

- https://openclipart.org/.

5) Please amend your detailed Financial Disclosure statement. This is published with the article. It must therefore be completed in full sentences and contain the exact wording you wish to be published.

1) State what role the funders took in the study. If the funders had no role in your study, please state: "The funders had no role in study design, data collection and analysis, decision to publish, or preparation of the manuscript."

2) If any authors received a salary from any of your funders, please state which authors and which funders..

If you did not receive any funding for this study, please simply state: u201cThe authors received no specific funding for this work.u201d

6)  Please ensure that the funders and grant numbers match between the Financial Disclosure field and the Funding Information tab in your submission form. Note that the funders must be provided in the same order in both places as well.

7) Kindly amend your competing statement in the online submission form to align with the journal's style guidelines: 'The authors declare that there are no competing interests.'

**Reviewers' comments:**

Reviewer's Responses to Questions

**Comments to the Authors:**

**Please note here if the review is uploaded as an attachment.**

Reviewer #1: This manuscript presents cellmig, an R/Bioconductor package that uses a Bayesian hierarchical model to estimate treatment effects on single-cell migration velocity while accounting for nested experimental structure (cells within wells within plates) and plate/batch effects. The core idea is solid, practical for high-throughput migration experiments, and the software packaging is a meaningful contribution. The main issue is positioning: the manuscript frames its benchmarking as exhaustive and state-of-the-art, but the comparisons as presented do not yet support that level of claim. For a PLOS Computational Biology methods paper, the authors should either strengthen benchmarking against appropriate modern baselines or tone down the “SOTA” language and present the work as a rigorous, uncertainty-aware alternative to common practice. As written, I recommend major revision.

Strengths

* Addresses a common failure mode in migration analysis (pseudo-replication and batch effects) while carrying uncertainty through to effect estimates.

* Statistically coherent approach (hierarchical partial pooling; effect sizes with credible intervals; plate normalization using matched controls) implemented in Stan and distributed via Bioconductor.

* Outputs are practical and interpretable for experimental users (fold-changes with uncertainty and probability-style evidence summaries).

* Validation includes meaningful real datasets, including large-scale screens, showing plate-aware inference at scale.

* The simulation component for experimental design (biological vs technical replicates) is a genuinely valuable feature that many pipelines lack.

Key weaknesses (what currently blocks acceptance)

* Benchmarking and positioning do not match: the manuscript implies state-of-the-art performance but does not yet compare against the most relevant modern hierarchical alternatives.

* The manuscript uses “false positive/false negative” language in contexts where ground truth is not established, which overstates what can be concluded from method disagreement.

* Uncertainty is emphasized for velocity summaries, but upstream measurement uncertainty (segmentation/tracking/filtering choices) is treated lightly, leaving potential bias and sensitivity underexplored.

* Practical deployment guidance is still thin for a screening-oriented tool (runtime/scaling expectations, diagnostics conventions, thresholding guidance, and multiple-testing framing).

* Robustness to common data pathologies (heavy tails, mixtures, outliers, fragmented tracks) needs clearer stress testing and user-facing guidance.

Major concern 1: Benchmarking and “state-of-the-art” positioning are currently misaligned with the comparisons shown. If the authors want to retain an “exhaustive/SOTA” framing, the benchmark needs to include stronger, field-standard approaches for nested high-content data, ideally chosen from methods with stable, open implementations so the comparison is transparent and reproducible. At a minimum, that typically means testing against multilevel/hierarchical baselines (e.g., mixed-effects modeling, or Bayesian multilevel regression implemented in widely used frameworks such as Stan interfaces like brms), and also considering distribution-robust options that respect nesting (e.g., resampling approaches designed for clustered data). The high-throughput screening literature also provides relevant comparators and evaluation concepts that could strengthen the benchmarking story—plate QC metrics like Z′, spatial normalization approaches such as B-score/median polish, and established Bioconductor tooling for plate-based or image-based screens (e.g., cellHTS2, imageHTS). In parallel, there is now precedent for benchmarking batch-correction methods directly in image-based profiling (Cell Painting) using quantitative metrics for both batch removal and biological signal preservation; if the manuscript claims robustness to batch effects, this kind of framing can help define what “robust” means and how to measure it. Alternatively, if the authors’ real target is to improve on common practice in migration workflows (two-stage summaries plus conventional tests), they should say so clearly and consistently and retire the “state-of-the-art” benchmarking language. That reframing does not eliminate novelty: the defensible contribution is an end-to-end, migration-tailored hierarchical workflow that unifies biological effects and batch/plate effects in one generative model and pairs inference with simulation-based experimental planning.

Major concern 2: The manuscript sometimes labels disagreements with the baseline analysis as “false positives” and “false negatives” without an independent truth standard. On real datasets, this is stronger than the evidence supports and reads as if the authors are declaring their own method the ground truth. A cleaner approach is to describe these as discordant calls and then argue, mechanistically and statistically, why one approach is more appropriate for the experimental structure at hand. If the authors want to make error-rate statements, they should confine those to settings where truth is known (e.g., simulations that include realistic plate/well/cell nesting and batch effects) or to external validation. More broadly, the screening literature already offers a vocabulary for decision-making that can be more informative than “false positive/false negative versus my method,” including effect-size and ranking metrics used for hit selection such as SSMD; positioning the decision layer in that ecosystem would strengthen the paper and make it more familiar to screening practitioners.

Major concern 3: The paper emphasizes uncertainty and probability-style evidence summaries, but it does not yet make a convincing case that these quantities are calibrated or how they should be used to make screening decisions at scale. The Bayesian workflow literature is blunt on this point: uncertainty intervals are only as good as the model and need to be interrogated with posterior predictive checks and sensitivity analysis, especially when the data-generating process is plausibly misspecified. This matters here because velocity distributions can be skewed and heavy-tailed, and can also be “structurally weird” due to tracking dropouts and filtering rules. The paper would be much stronger if it demonstrated that its uncertainty statements behave sensibly under realistic conditions and then explained, in practical terms, how an analyst should use those quantities to prioritize treatments in large screens (including how the approach interacts with multiple testing or multi-condition ranking).

Major concern 4: Robustness to model misspecification and common migration-data artifacts needs more emphasis. Migration velocity estimates can be sensitive to outliers, track fragmentation, and heterogeneity that looks more like mixtures than a single clean distribution. The manuscript would benefit from explicitly acknowledging these failure modes and showing that conclusions are either stable to them or that the tool provides clear indicators when they are not. This is also where the existing ecosystem provides useful context: in many migration settings, investigators rely on mature tracking and analysis toolchains (e.g., TrackMate, u-track, CellProfiler) and the broader community has developed benchmark standards for tracking performance (e.g., the Cell Tracking Challenge). If the paper’s downstream inference depends heavily on tracking-derived velocities, it should engage with that reality and help users understand when measurement artifacts could dominate the inference.

Major concern 5: The manuscript should more clearly delimit what is modeled and what is not with respect to upstream image analysis. Right now, the inference layer risks being read as more “uncertainty-aware” than it truly is, because segmentation/tracking uncertainty is not explicitly modeled and filtering choices can change the effective population being analyzed. The most convincing way to handle this is straightforward: state the scope clearly, then show sensitivity analyses that demonstrate how robust the reported treatment effects are to plausible variations in preprocessing and QC. The paper would also benefit from stronger evidence of measurement fidelity (clearer overlays of raw images with segmentation and tracks, and reporting of basic QC summaries such as track-length distributions and exclusion rates), so that readers can judge whether the velocity measurements are trustworthy inputs to a hierarchical inference engine.

Minor concern 1: Figure clarity, especially for segmentation/tracking examples, should be improved so readers can actually evaluate measurement fidelity. If space is tight in the main text, the supplement is fine, but the examples need to be legible and representative.

Minor concern 2: The manuscript should ensure consistent handling of fold-change null references. If the null is 1 on the natural scale and 0 on the log scale, that needs to be stated and used consistently across axes, captions, and legends to avoid confusion.

Minor concern 3: A short scope statement would help. Readers should know exactly which migration paradigms the method targets (single-cell velocity summaries in high-throughput, plate-based assays) and which common paradigms it is not aiming to replace (e.g., scratch/wound-closure analysis pipelines such as TScratch, broader end-to-end migration environments like CellMissy, or collective-migration workflows that use optical-flow/PIV rather than single-cell tracking). This also helps prevent overgeneralization of “velocity” across biological regimes.

Minor concern 4: Reproducibility and deployment details should be tightened. Readers will expect basic guidance on versions, typical compute requirements, and a minimal end-to-end script or vignette that reproduces a primary analysis.

Minor concern 5: Copyediting would improve readability. Tightening informal phrasing and standardizing terminology throughout would make the manuscript feel more careful and reduce opportunities for misinterpretation.

Closing

I view cellmig as promising and potentially impactful because it brings replicate-aware inference and uncertainty reporting to a workflow that is often analyzed with overconfident pooled statistics. If the authors either (a) broaden benchmarking to match the strength of their claims—using appropriate hierarchical baselines and established screening/batch-effect evaluation concepts—or (b) adjust the claims to match the evidence while keeping the methodological contribution front and center, and they strengthen calibration/robustness/QC guidance, I would be supportive of publication.

Reviewer #2: The authors present a Bayesian hierarchical approach to analysis high throughput data related to cell migration. An important component to their approach is the explicit modelling of biological and technical heterogeneity, which is often overlooked in many studies due to pooling of data across technical replicates/batches. They also provide a R package called ``cellmig'' to provide their tools to the research community.

I think this work is timely and valuable contribution to the literature, however, there are a number of concerns I have in relation general presentation of the work and with respect to some claims and results. If the authors are able to satisfactorily address my concerns, then I think it would be suitable for publication in PLOS Computational Biology.

Major Comments:

1. General presentation: I find the manuscript which confusing to read its current layout. It actually feels like the work was originally written for a Journal with section orders of Introduction -> Results -> Discussion -> Materials and Methods (e.g., like many general science journals). I found the transition from Introduction to Materials and Method to be incredibly jarring with the Materials and Methods being full of undefined acronyms and jargon, most of which was not at all properly defined until the Results section.

2. Contribution: It is difficult for me to identify what the main contribution to paper is presenting. Is it the cellmig package itself? or is it the modelling and the demonstration of the importance of dealing with technical/biological heterogeneity in a hierarchical sense? A clear contribution statement in the introduction would benefit the paper substantially. There also needs to be more clarity in exactly what the cellmig R package is providing. For example, if the cellmig restricted to the hierarchical model presented? What does it provide that other Bayesian tools for say Generalised linear/non-linear mixed-effects models would provide (like BRMS). The model that is presented in Equations (1)--(11) is not novel in of itself, so this all needs more clarity for me. Finally, the claim about the generative features within cellmig is not a contribution really, as this is an automatic feature of any parametric model (Bayesian or not), and the authors concepts of fully generative and partially generative is effectively equivalent to prior and posterior predictive sampling which is standard practice. To be clear, I am not saying that this is not a valid thing to do, but the claims of novelty here are tool strong.

3. Modelling: I am generally comfortable with the modelling itself, but I think the choices around priors are not clear at all. The section titled ``Model priors'' on page 10 state ``Wherever possible, we used priors informed by the literature, otherwise employing weakly informative priors ... ' ' however, there is not a single reference to the literature in this section, nor would I consider a normal prior with variance of 1 to be weakly informative, especially for a scale parameter. The Supplementary figures referred to are useful, but they are not justification for the prior choice alone. The priors obtained form the literature should be justified by clear reference to the relevant literature and the claim of weakly informative priors should also be justified (e.g., Gelman text book cited elsewhere discusses this quite a bit in the context of the schools example).

4. Results: There are two main concerns about the results:

4.1 The comparison between the probability of differential effect on migration $\pi$ given in Equation (8) and the

outcome of a frequentist hyponthesis test using a non-parametric test. This is statistically invalid from my

perspective as Bayesian methods do not have statistical converage guarentees at all and the definition of $\pi$ is

simply not comparable to a p-value. Furthermore, a comparison of non-parametric methods with a parametric

model is also not valid. To properly evaluate the improvements of cellmig, an equivalent Bayesian model that

ignores the hierarchical structure would be needed. Such a comparison would ideally include prior sensitivity

analysis.

4.2 The experimental design results and associated claims are potentially extremely biased as the simulations are

effectively posterior predictive samples. So it is unclear how generalisable these results are. I wonder if a prior

predictive approach would be better to highlight the value of the cellmig tools.

5. Software: I was a bit surprised given the emphasis on the cellmig package that there was not clear description of it anywhere. I think the paper would benefit from a short appendix/supplementary description with example code demonstrating the main features of the software.

Minor comments:

1. The next revision should include line numbers, it is quite difficult to provide minor feedback without line numbers.

2. throughout, can Curiour New font be used for the cellmig software name.

3. The abstracts reference to the detail of the experiments are too vague. just remove them, that is the number of cells and conditions.

4. Page 3, can you provide references for the ``Diverse experimental methods...''

5. Page 3, what you you mean by high-throughput?

6. Page 4, I dont understand the point of the ``simple plots'' as being a methodological issue, this is a communication issue.

7. Generally I find the methods section to be too vague. (e.g., what is the difference between A & B in dataset 1) especially page 4 to 7.

8. Page 10 remove the $\rho = $ in the integral lower bounds in equation (8).

9. Page 15, a Wilcoxon rank-sum test and Kruskal-Wallis text etc are not state-of-the-art, they are classical and standard methods, but not state-of-the-art.

**********

**Have the authors made all data and (if applicable) computational code underlying the findings in their manuscript fully available?**

The PLOS Data policy requires authors to make all data and code underlying the findings described in their manuscript fully available without restriction, with rare exception (please refer to the Data Availability Statement in the manuscript PDF file). The data and code should be provided as part of the manuscript or its supporting information, or deposited to a public repository. For example, in addition to summary statistics, the data points behind means, medians and variance measures should be available. If there are restrictions on publicly sharing data or code —e.g. participant privacy or use of data from a third party—those must be specified.

Reviewer #1: Yes

Reviewer #2: **No:**  The software is provided but not sufficient to reproduce the analysis.

**********

PLOS authors have the option to publish the peer review history of their article (what does this mean?). If published, this will include your full peer review and any attached files.

If you choose “no”, your identity will remain anonymous but your review may still be made public.

**Do you want your identity to be public for this peer review?**  For information about this choice, including consent withdrawal, please see our Privacy Policy.

Reviewer #1: **Yes:**  Francesco Pasqualini

Reviewer #2: No

**Figure resubmission:**

While revising your submission, we strongly recommend that you use PLOS’s NAAS tool (https://ngplosjournals.pagemajik.ai/artanalysis) to test your figure files. NAAS can convert your figure files to the TIFF file type and meet basic requirements (such as print size, resolution), or provide you with a report on issues that do not meet our requirements and that NAAS cannot fix.

After uploading your figures to PLOS’s NAAS tool - https://ngplosjournals.pagemajik.ai/artanalysis, NAAS will process the files provided and display the results in the "Uploaded Files" section of the page as the processing is complete. If the uploaded figures meet our requirements (or NAAS is able to fix the files to meet our requirements), the figure will be marked as "fixed" above. If NAAS is unable to fix the files, a red "failed" label will appear above. When NAAS has confirmed that the figure files meet our requirements, please download the file via the download option, and include these NAAS processed figure files when submitting your revised manuscript.

**Reproducibility:**

To enhance the reproducibility of your results, we recommend that authors of applicable studies deposit laboratory protocols in protocols.io, where a protocol can be assigned its own identifier (DOI) such that it can be cited independently in the future. Additionally, PLOS ONE offers an option to publish peer-reviewed clinical study protocols. Read more information on sharing protocols at https://plos.org/protocols?utm_medium=editorial-email&utm_source=authorletters&utm_campaign=protocols

# Author comment: Author response to Decision Letter 1

**DOI:** 10.1371/journal.pcbi.1014472.r003

2 Jun 2026

Attachment Submitted filename: Rebuttal_Response_to_Reviewers.pdf

# Peer review: Decision Letter 1

**DOI:** 10.1371/journal.pcbi.1014472.r004

**Authors:** Dimitrios Vavylonis

17 Jun 2026

Dear Mr. Kitanovski,

We are pleased to inform you that your manuscript 'Uncertainty-aware quantitative analysis of high-throughput live cell migration data' has been provisionally accepted for publication in PLOS Computational Biology.

Before your manuscript can be formally accepted you will need to complete some formatting changes, which you will receive in a follow up email. A member of our team will be in touch with a set of requests.

Please note the reviewer's remark regarding providing a reference to the data used in the software.

Please note that your manuscript will not be scheduled for publication until you have made the required changes, so a swift response is appreciated.

IMPORTANT: The editorial review process is now complete. PLOS will only permit corrections to spelling, formatting or significant scientific errors from this point onwards. Requests for major changes, or any which affect the scientific understanding of your work, will cause delays to the publication date of your manuscript.

Should you, your institution's press office or the journal office choose to press release your paper, you will automatically be opted out of early publication. We ask that you notify us now if you or your institution is planning to press release the article. All press must be co-ordinated with PLOS.

Thank you again for supporting Open Access publishing; we are looking forward to publishing your work in PLOS Computational Biology.

Best regards,

Dimitrios Vavylonis

Section Editor

PLOS Computational Biology

***********************************************************

Reviewer's Responses to Questions

**Comments to the Authors:**

**Please note here if the review is uploaded as an attachment.**

Reviewer #1: The authors addressed all my concerns.

Reviewer #2: I thank the authors for their careful consideration of my comment. I am pleased with the revision. The paper is now much clearer in its flow, presents a better case of novelty (focusing on the computational tools, rather than the modelling contribution), presents more principled analysis, and make more appropriate statistical claims while avoiding possible miss -understandings in interpretation.

I have not additional comments to make, I think the article is now appropriate for publication.

**********

**Have the authors made all data and (if applicable) computational code underlying the findings in their manuscript fully available?**

The PLOS Data policy requires authors to make all data and code underlying the findings described in their manuscript fully available without restriction, with rare exception (please refer to the Data Availability Statement in the manuscript PDF file). The data and code should be provided as part of the manuscript or its supporting information, or deposited to a public repository. For example, in addition to summary statistics, the data points behind means, medians and variance measures should be available. If there are restrictions on publicly sharing data or code —e.g. participant privacy or use of data from a third party—those must be specified.

Reviewer #1: Yes

Reviewer #2: **No:**  Software seems to be available, but I could not find any reference to the data used.

**********

PLOS authors have the option to publish the peer review history of their article (what does this mean?). If published, this will include your full peer review and any attached files.

If you choose “no”, your identity will remain anonymous but your review may still be made public.

**Do you want your identity to be public for this peer review?**  For information about this choice, including consent withdrawal, please see our Privacy Policy.

Reviewer #1: **Yes:**  Francesco Pasqualini

Reviewer #2: No

# Editor report: Acceptance letter

**DOI:** 10.1371/journal.pcbi.1014472.r005

**Authors:** Dimitrios Vavylonis

PCOMPBIOL-D-26-00296R1

Uncertainty-aware quantitative analysis of high-throughput live cell migration data

Dear Dr Kitanovski,

I am pleased to inform you that your manuscript has been formally accepted for publication in PLOS Computational Biology. Your manuscript is now with our production department and you will be notified of the publication date in due course.

The corresponding author will soon be receiving a typeset proof for review, to ensure errors have not been introduced during production. Please review the PDF proof of your manuscript carefully, as this is the last chance to correct any errors. Please note that major changes, or those which affect the scientific understanding of the work, will likely cause delays to the publication date of your manuscript.

Soon after your final files are uploaded, unless you have opted out, the early version of your manuscript will be published online. The date of the early version will be your article's publication date. The final article will be published to the same URL, and all versions of the paper will be accessible to readers.

For Research, Software, and Methods articles, you will receive an invoice from PLOS for your publication fee after your manuscript has reached the completed accept phase. If you receive an email requesting payment before acceptance or for any other service, this may be a phishing scheme. Learn how to identify phishing emails and protect your accounts at https://explore.plos.org/phishing.

Thank you again for supporting PLOS Computational Biology and open-access publishing. We are looking forward to publishing your work!

With kind regards,

Sharmila Kamatchi

PLOS Computational Biology | Carlyle House, Carlyle Road, Cambridge CB4 3DN | United Kingdom ploscompbiol@plos.org | Phone +44 (0) 1223-442824 | ploscompbiol.org | @PLOSCompBiol
